# Unveiling epithelial plasticity regulation in lung cancer: Exploring the cross-talk among Tks4 scaffold protein partners

**DOI:** 10.1091/mbc.E24-03-0103

**Published:** 2024-07-22

**Authors:** Loretta László, Anita Kurilla, Álmos Tilajka, Rita Pancsa, Tamás Takács, Julianna Novák, László Buday, Virag Vas

**Affiliations:** a Institute of Molecular Life Sciences, HUN-REN Research Centre for Natural Sciences, 1117 Budapest, Hungary; b Doctoral School of Biology, Institute of Biology, ELTE Eötvös Loránd University, 1117 Budapest, Hungary; c Department of Molecular Biology, Semmelweis University, 1094 Budapest, Hungary; University of California, San Francisco

## Abstract

The epithelial-to-mesenchymal transition (EMT) represents a hallmark event in the evolution of lung cancer. This work aims to study a recently described EMT-regulating protein, Tks4, and to explore its potential as a prognostic biomarker in non–small cell lung cancer. In this study, we used CRISPR/Cas9 method to knockout (KO) Tks4 to study its functional roles in invadopodia formation, migration, and regulation of EMT marker expressions and we identified Tks4-interacting proteins. Tks4-KO A549 cells exhibited an EMT-like phenotype characterized by elongated morphology and increased expression of EMT markers. Furthermore, analyses of a large-scale lung cancer database and a patient-derived tissue array data revealed that the Tks4 mRNA level was decreased in more aggressive lung cancer stages. To understand the regulatory role of Tks4 in lung cancer, we performed a Tks4-interactome analysis via Tks4 immunoprecipitation-mass spectrometry on five different cell lines and identified CAPZA1 as a novel Tks4 partner protein. Thus, we propose that the absence of Tks4 leads to disruption of a connectome of multiple proteins and that the resulting undocking and likely mislocalization of signaling molecules impairs actin cytoskeleton rearrangement and activates EMT-like cell fate switches, both of which likely influence disease severity.

## Abstract (executive summary)

This study demonstrated the relevance of Tks4 in the regulation of an EMT-like process in lung cancer based on a CRISPR/Cas9 KO lung cancer model.

Mass spectrometry analysis identified CAPZA1 as a Tks4-associated novel binding partner and member of the Tks4 interactome.

Database analysis revealed that the Tks4-organized interactome impacts the regulation of epithelial plasticity in lung cancer and revealed that Tks4 might serve as a diagnostic biomarker for lung cancer.

## INTRODUCTION

Epithelial-to-mesenchymal transition (EMT) is a complex multistep process in which epithelial cells undergo phenotypic changes, thereby acquiring a more motile mesenchymal cell phenotype. During EMT in cancer, there are dynamic changes in the transcriptome and proteome of epithelial cancer cells, leading to the acquisition of migratory abilities necessary for metastasis formation. The dynamic nature of this process results in a heterogeneous cell population within the cancer tissue that comprises cancer cells at different EMT stages. This phenotypic heterogeneity, in which multiple hybrid EMT-like states coexist in the cancer cell population, is the main characteristic of epithelial plasticity; furthermore, it enables rapid adaptive responses to stress and anti-cancer drugs, thereby promoting cancer progression (Bornes *et al.*, 2021). Recent clinical evidence has also demonstrated that the coexpression of epithelial and mesenchymal markers is associated with poor patient survival in non–small cell lung cancer (NSCLC) (Zacharias *et al.*, 2018). Furthermore, it has also shown that EMT markers expression levels during the initial steps of lung cancer development (i.e., stage IA) might serve as a marker for shorter overall survival (Matsubara *et al.*, 2019). EMT is not a binary switch at the cellular or cancer population level; rather, it represents a continuum of phenotypic changes. During the EMT phase of tumor progression, both epithelial markers (such as E-cadherin) and mesenchymal markers (e.g., N-cadherin, vimentin, and fibronectin) are simultaneously present on cancer cells, gradually hindering their cell-cell contacts (Saitoh, 2018). This type of epithelial plasticity in cancer cells is associated with a higher tumor-initiating ability compared with fully epithelial or fully mesenchymal cancer cells (Jolly *et al.*, 2015; Kröger *et al.*, 2019); therefore, understanding the mechanisms regulating the spectrum of EMT states can provide insight into cancer progression and the potential clinical application of EMT inhibition.

Tks4 is a scaffold protein that contains one PX domain and four SH3 domains connected by intrinsically disordered linkers of varying lengths, and it serves as an adaptor component of the EGFR/SRC signaling pathway (Bögel *et al.*, 2012; Dülk *et al.*, 2018). The regulatory role of Tks4 has been demonstrated in noncancerous cells, such as adipocytes, osteoblasts, mesenchymal stem cells, and during embryonic stem cell differentiation (Dülk *et al.*, 2016; László *et al.*, 2022). Tks4 was originally recognized for its involvement in invadopodia/podosome formation in cancerous and in normal cell, respectively (Iizuka *et al.*, 2016). It acts as a scaffold for proteins involved in actin cytoskeleton remodeling, for example, cortactin, Grb2 (growth factor receptor-bound protein 2) and N-WASP (neural Wiskott-Aldrich syndrome protein). Invadopodia formation, which is specific to cancer cells, plays a crucial role in the metastasis by facilitating the reorganization of cytoplasmic molecules and enabling cell invasion into the surrounding stroma. Cortactin, which is often used as an invadopodia marker, directly associates with Tks4 and Tks5. Tks5, structurally homologous protein to Tks4 but has one more SH3 domain and represents also a critical component of invadopodia formation (Buschman *et al.*, 2009; Leong *et al.*, 2014; Kudlik *et al.*, 2020; Linder *et al.*, 2023).

This study focuses on a recently identified novel function of Tks4 in modulating an EMT-like process. Szeder *et al.* revealed that the lack of Tks4 induces an EMT-like process in epithelial HCT116 cancer cells (Szeder *et al.*, 2019). Further examination by Jacksi *et al.* expanded our knowledge of Tks4’s function in EMT process in colon cancer, and confirmed the notion that Tks4 is more than an invadopodia organizing scaffold protein and that regulates signaling networks during EMT (Jacksi *et al.*, 2023). Their study showed that the lack of Tks4 in carcinoma cells disturbs several signaling pathways, including PI3K/AKT, MAPK/ERK, p53) at multiple level, that is, the mRNA transcriptome and the expression levels of long noncoding RNA, the proteome and reactome (Jacksi *et al.*, 2024). Although Tks4’s involvement in EMT initiation has already been identified, further investigation is needed to shed light on the mechanisms by which complex Tks4-dependent signaling pathways exert their effects in cancer cells. We hypothesized that Tks4 likely interacts directly with EMT modulating partner molecules, in addition to the invadopodia organizing proteins, to affect EMT. Supporting this hypothesis, we previously identified CD2-associated protein (CD2AP) as a direct Tks4-interacting partner and demonstrated that Tks4 and CD2AP form a complex that potentially regulates an EMT-like process during colon cancer development (Kurilla *et al.*, 2023).

Our current understanding that Tks4 is a part of a signaling molecule complex that defines the epithelial or mesenchymal characteristics of colon cancer cells led us to study whether Tks4 also functions as an EMT regulator in lung cancer. We also hypothesized that the versatility of Tks4 in cancer cells is achieved via interaction with its numerous (known and novel) binding partners, forming a complex network that regulates actin cytoskeleton rearrangements, invadopodia formation, and the EMT process, as well. Despite the significance of this protein in colon cancer formation, its mechanisms of action have not been elucidated in other cancer types.

To address this hypothesis, we used a similar approach previously used to study colon cancer cells. We knocked down Tks4 expression in a lung adenocarcinoma cell line (A549) and then examined a canonical Tks4-related phenotype, that is, altered invadopodia formation. We also investigated the changes in the EMT-like process in the Tks4-knockout (KO) cells. To identify potential pathways related to Tks4 and EMT, we used a Tks4 immunoprecipitation (IP)-mass spectrometry (MS) method to identify novel Tks4 interactors and analyzed the Tks4-associated signaling proteins and their links to EMT regulation in three different lung cancer cell lines (A549, HOP-92, NCI-H460). Furthermore, we used NSCLC cancer patient samples and analyzed relevant data from The Cancer Genome Atlas (TCGA) database to evaluate the impact of Tks4 levels on the progression of NSCLC adenocarcinoma.

Our findings demonstrate that Tks4 and its interacting partners are integral components of the EMT-regulating signaling pathway. Additionally, the Tks4 level shows promise as a potential biomarker for cancer development, providing a novel perspective in lung cancer research.

## RESULTS

### The impact of Tks4 knockdown on invadopodia formation and cell migration

Tks4 knockdown has been shown to induce an EMT-like phenotype in colon cancer cells; however, the role of Tks4 downregulation in the EMT process in other cancer types has not been established. The A549 lung cancer cell line was selected as a model for assessing the effect of Tks4 protein depletion on EMT in lung cancer. A549 cells express measurable levels of Tks4 and exhibit an epithelial phenotype, making them widely used in lung cancer research and ideal for studying EMT (Dong *et al.* 2017; Schelch *et al.*, 2021).

**FIGURE 1:**
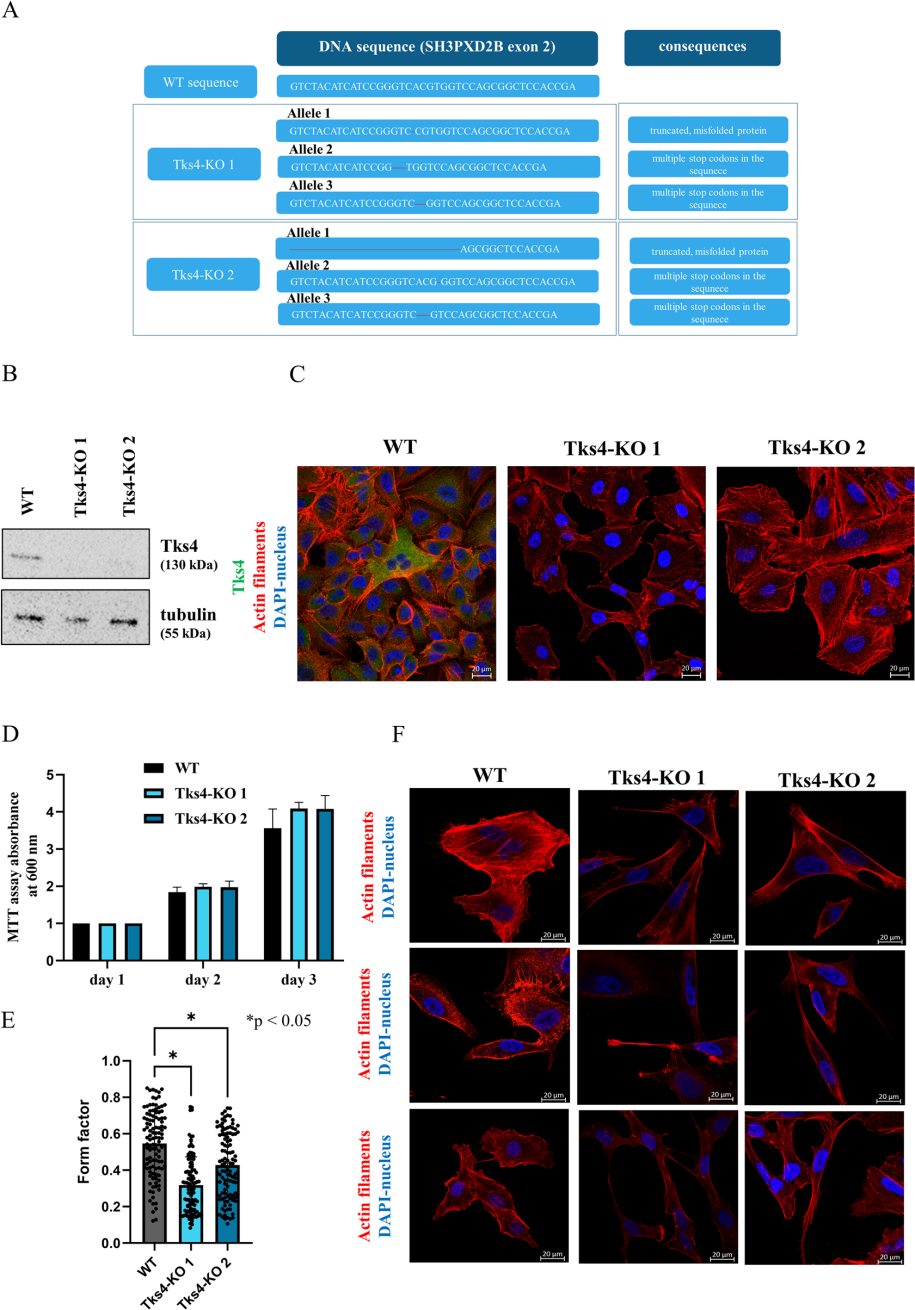
Characterization of Tks4-deficient A549 human lung adenocarcinoma cells. (A) DNA sequence changes in the Tks4-KO clones (deletion events marked in red) after CRISPR-Cas9 genome editing and their predicted consequences at the protein level. The reference (WT) Tks4 sequence (NM_001017995.3) was downloaded from NCBI GenBank. (B) Validation of the absence of Tks4 in Tks4-KO cells via WB. (C) Validation of the absence of Tks4 in Tks4-KO cells via ICC (magnification: 40x). (D) Comparison of the proliferative capacities of Tks4-KO and WT cells measured via MTT assay. (E) The form factor is reported as values between 0 and, where a circular cell shape is indicated by values closer to 1 (*n* = 110 per each cell type). (F) F-actin ICC staining to demonstrate changes in cell morphology. Representative confocal images showing the elongated morphology of Tks4-KO cells (magnification: 63x). Nuclei are stained with DAPI (blue), and actin filaments are stained with phalloidin (red). The experiment was repeated three times, and five random fields were captured per experiment.

To this end, we used the CRISPR/Cas9 system to mutate the SH3PXD2B gene, which encodes the Tks4 protein. We then characterized the A549 cells, and the karyogram showed that they contain three copies of chromosome 5 and, consequently, have three different *SH3PXD2B* alleles (Supplemental Figure S1A). Two Tks4-KO A549 cell lines were generated, each with distinct genetic alterations only in exon 2 of SH3PXD2B (Figure 1A). Sanger sequencing showed that both Tks4-KO A549 clones carried deletion mutations in all three alleles due to truncating mutations or frameshift mutations introducing multiple stop codons within the N-terminal PX domain of the Tks4 protein sequence and thereby truncating the protein. Otherwise, the cell line authentication analysis presented that the generated clones match 100% to the DNA of the wild-type (WT) A549 cells (Supplemental Figure S1B). The absence of Tks4 protein was confirmed via Western blotting (WB) and immunocytochemistry (ICC) in both clones (Figure 1, B and C). Notably, the mutations in *SH3PXD2B* did not affect the proliferative capacity of the cells, as shown by an MTT assay (Figure 1D); however, the morphology of the Tks4-KO A549 clones was altered as the cells exhibited a spindle-like shape (Figure 1F). Moreover, the F-actin stained cells were subjected to form factor quantification, where a value closer to 1 implies a more circular shape (Szebényi *et al.*, 2023). The statistical analysis revealed that the Tks4-KO clones displayed significantly lower form factor values (Figure 1E), indicating that WT cells are significantly more circular in shape than Tks4-KO cells.

The podosome/invadopodia-forming role of Tks4 has already been confirmed in many cell types but not yet in lung cancer cells. To investigate this function in WT and Tks4-KO cells, we examined the level and localization of the invadopodia marker Tks5 and cortactin.

**FIGURE 2:**
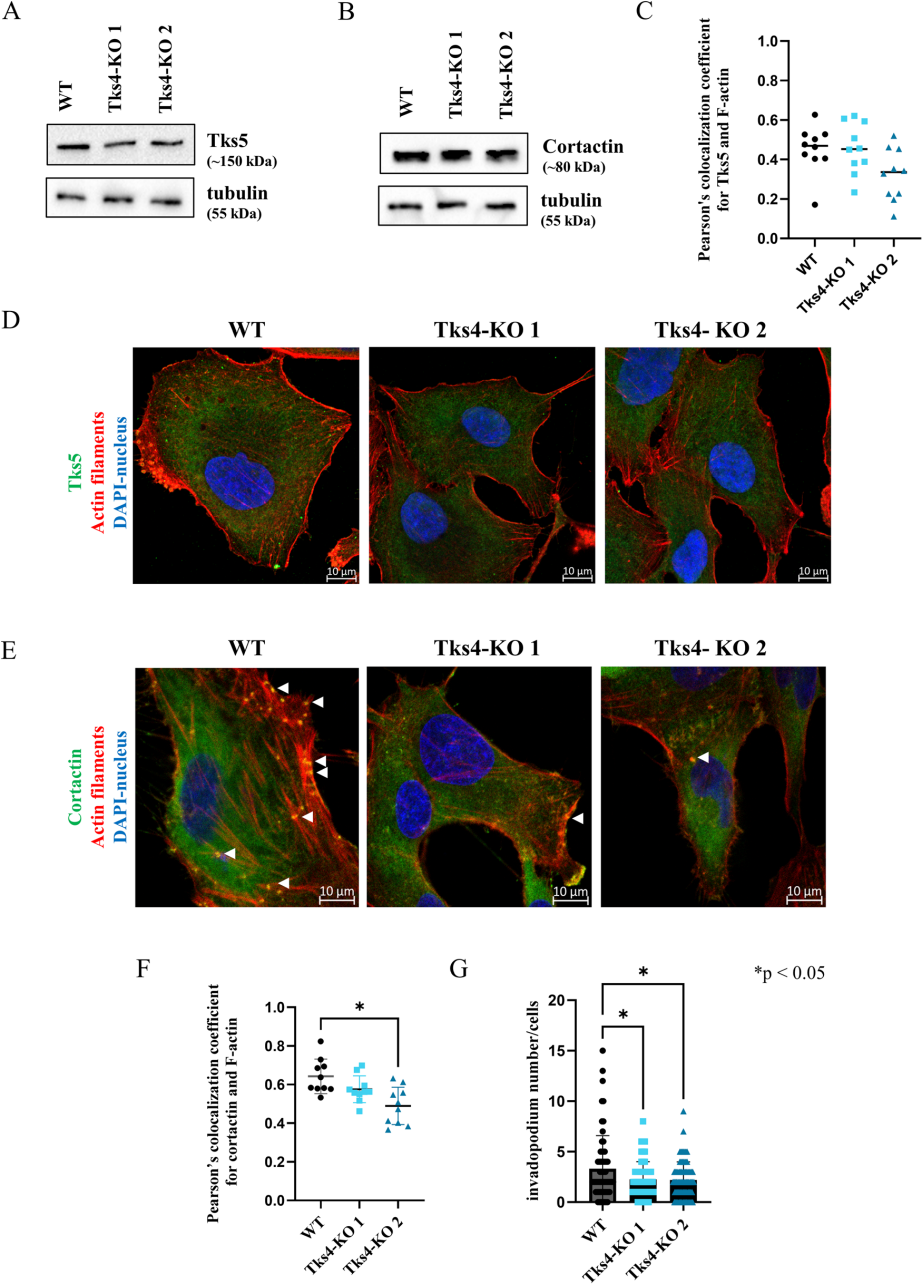
The impact of Tks4 deletion on invadopodia formation of lung cancer cells. WB analysis for Tks5 (A) and for cortactin (B). The C panel represents the calculated Pearson’s colocalization coefficient for F-actin and Tks5. (D) ICC costaining of Tks5 with F-actin (confocal images, magnification: 63x). (E) ICC costaining of key invadopodia elements, F-actin (red) and cortactin (green), revealing orange dots that mark the invadopodia-like structures (white arrows) (confocal images, magnification: 63x). (F) Overall colocalization for F-actin and cortactin based on Pearson’s coefficient quantification. (G) Quantitative analysis of invadopodia number based on cortactin and F-actin colocalizations in circular dot-like structures per cells. The diagram shows the mean number of invadopodia per cell (each point corresponds to a single cell).

WB results show that the Tks4 knockdown did not affect Tks5 and cortactin protein expression levels (Figure 2, A and B). Subsequently, cells were costained for Tks5 or cortactin with F-actin and Pearson’s coefficient was calculated (Figure 2, C–F). In case of the Tks5/F-actin staining, colocalization was relatively low in WT A549 cells, and showed no significant change in the Tks4-KO clones (Figure 2C). In contrast, the Pearson’s coefficient of cortactin/F-actin staining was high in the WT cells, and significantly reduced in Tks4-KO1 cells (Figure 2F). For these reasons and because cortactin and F-actin are widely used markers for invadopodia structures, we have used these markers to identify specifically the invadopodia structures in these cells (Murphy and Courtneidge, 2012; Wang *et al.*, 2013; Juin *et al.*, 2014; Li *et al.* 2018). The number of the cortactin/F-actin double-stained circular dot-like structures at the membrane was quantified and represented as the invadopodia number per cell. Analysis of cortactin/F-actin positive foci shows that the number of invadopodia structures is reduced in both Tks4-KO cells (Figure 2G).

Considering that invadopodia are membrane structures used by cancer cells for movement and are generally linked to the regulation of migration, we examined the intrinsic migration capacity of A549 cells via an in vitro scratch assay.

**FIGURE 3:**
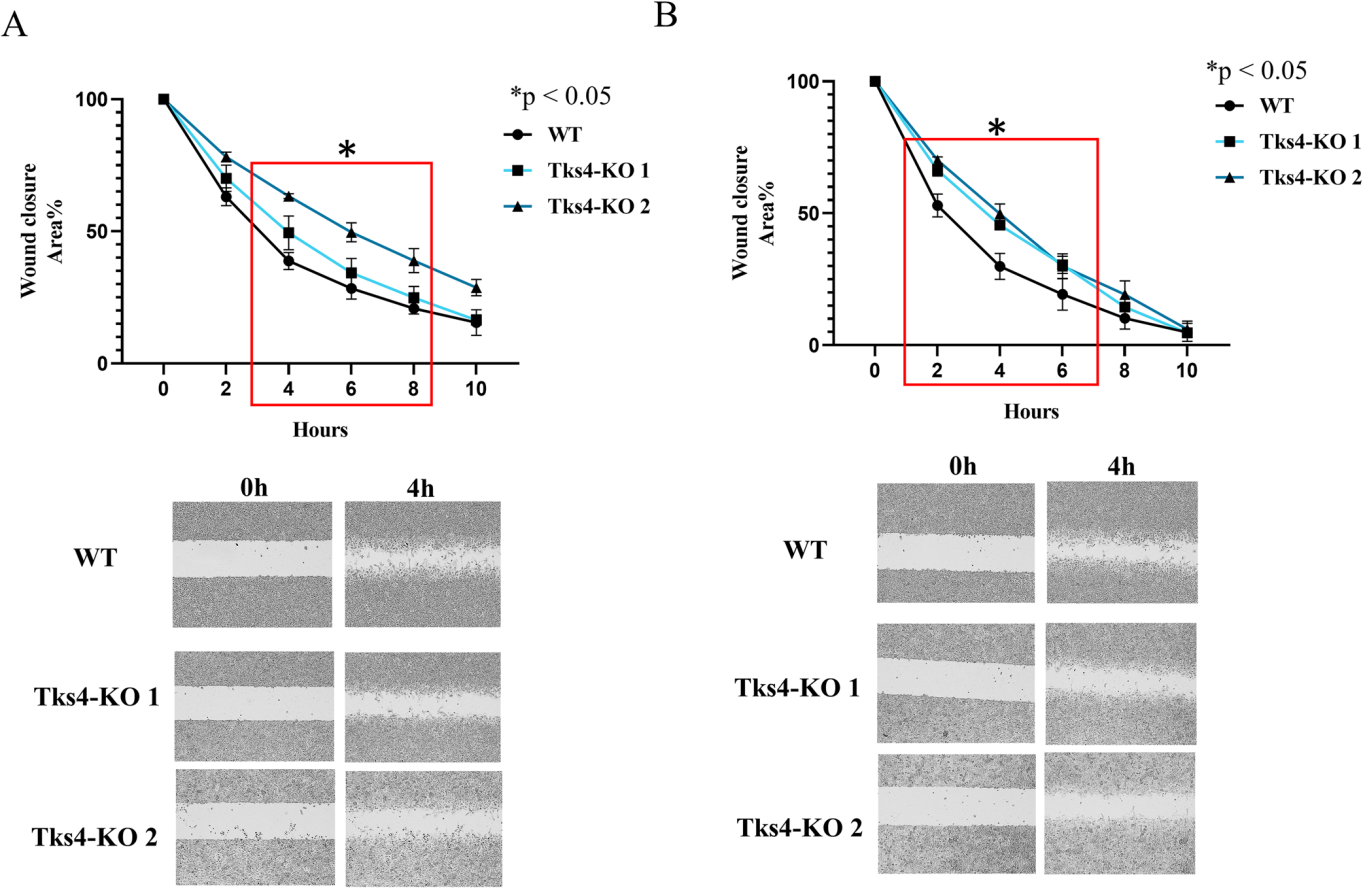
The migration ability of the Tks4-KO lung cancer cells. Results of the wound healing assay. (A) Cell migration of Tks4-KO cells in medium. (B) The effect of EGF treatment (50 ng/ml) on cell migration. The red boxes indicate statistically significant differences in both Tks4-KO cell lines. The experiments were repeated twice.

These results revealed a slight reduction in the migration capacity of Tks4-KO cells compared with WT cells (Figure 3A). Notably, previous studies have demonstrated that EGF can enhance lung cancer cell migration (Schelch *et al.*, 2021), thus, we also examined the effect of EGF treatment on the migration ability of Tks4-KO cells. The results showed that WT cells exhibited a higher migration rate upon EGF stimulation than Tks4-KO cells (Figure 3B). This observation suggests that Tks4 knockdown did not enhance the migration capacity of the cells, even in the presence of EGF treatment.

### The impact of Tks4 knockdown on the EMT-like process

**FIGURE 4:**
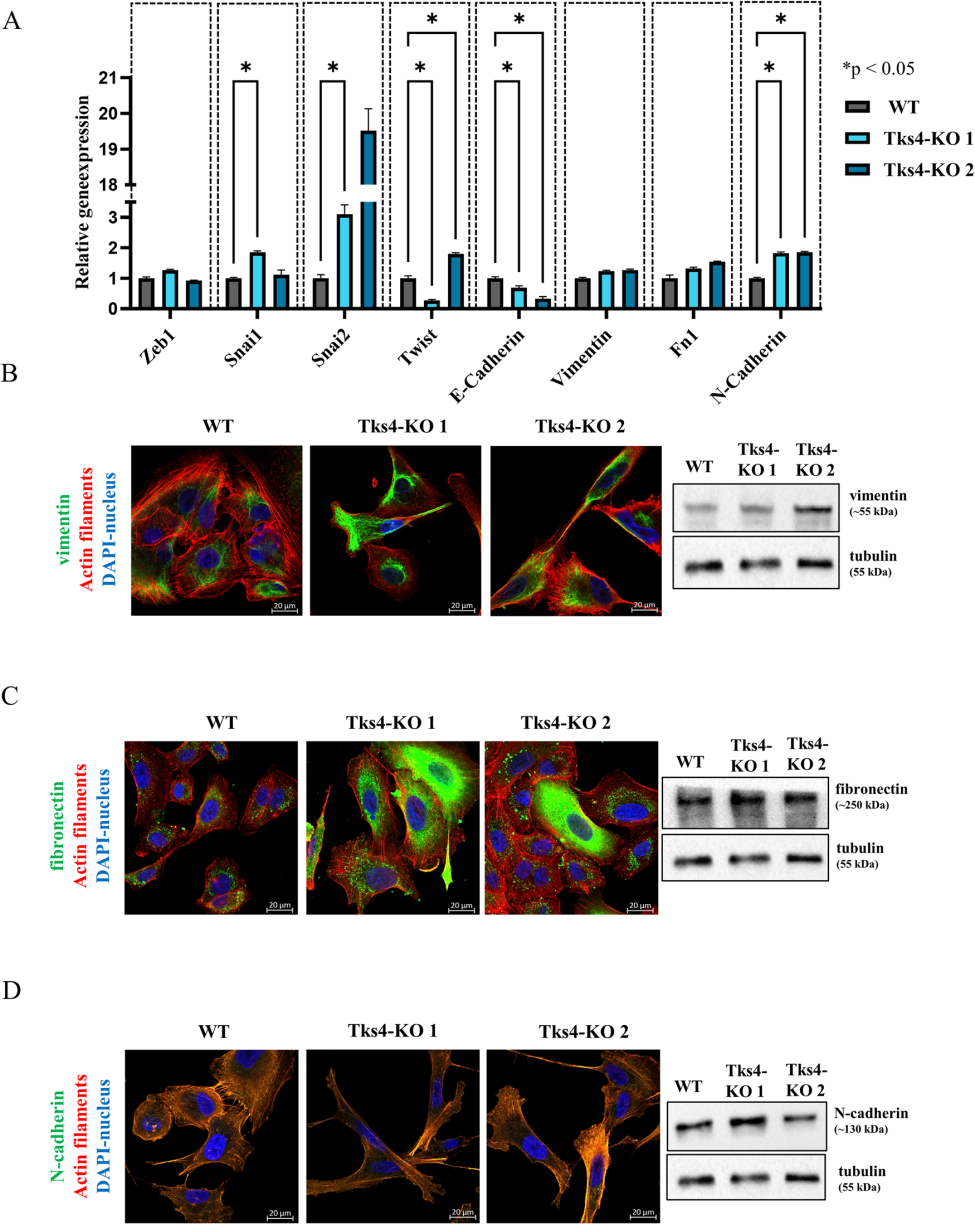
Changes in the expression levels of EMT-related markers in Tks4-KO cells measured by qRT-PCR, ICC, and WB. (A) qRT-PCR analysis of key EMT-related transcription factors (Zeb1, Snail1, Snail2, Twist) and epithelial-to-mesenchymal markers (N-cadherin, E-cadherin, Vimentin, Fibronectin [Fn1]). Gene expression levels were normalized to GAPDH. The localization of vimentin (B), fibronectin (C), N-cadherin (D) (Alexa Fluor 488-green) costained with F-actin (Phalloidin-red) were visualized via ICC (confocal images, magnification: 63x images). The level of these proteins was measured via WB (right panels).

Next, we measured the changes in EMT markers levels following Tks4 knockdown using qRT-PCR, WB, and ICC to investigate the recently discovered but less-understood effect of Tks4 on the EMT process in lung cancer cells (Figure 4).

The qPCR results revealed that EMT-related transcription factors (Zeb1, Snai1, Snai2, Twist) and mesenchymal markers (fibronectin, vimentin, N-cadherin) were expressed at a basal level in WT A549 cells; however, in both Tks4-KO clones, the expression levels of Snai2, fibronectin, vimentin, N-cadherin were elevated. Additionally, one Tks4-KO clone showed increased mRNA levels of transcription factors Snai1 and Twist (Figure 4A). Parallel with this, the epithelial marker E-cadherin mRNA level was decreased in Tks4-KO cells. N-cadherin, vimentin, and fibronectin were also analyzed by WB and ICC, and revealed that Tks4-KO cells expressed vimentin and fibronectin at higher proteins levels than WT cells but the level of N-cadherin remained unchanged (Figure 4, B–D).

The altered expression levels of these markers, particularly fibronectin, vimentin, Snai2, N-cadherin, and E-cadherin at mRNA level, along with the observed elongated morphology of Tks4-KO cells (Figure 1, E and F), imply that the Tks4-KO cells have an EMT-like phenotype with regard to this EMT marker profile.

### Downregulation of Tks4 expression accompanies NSCLC progression

**FIGURE 5:**
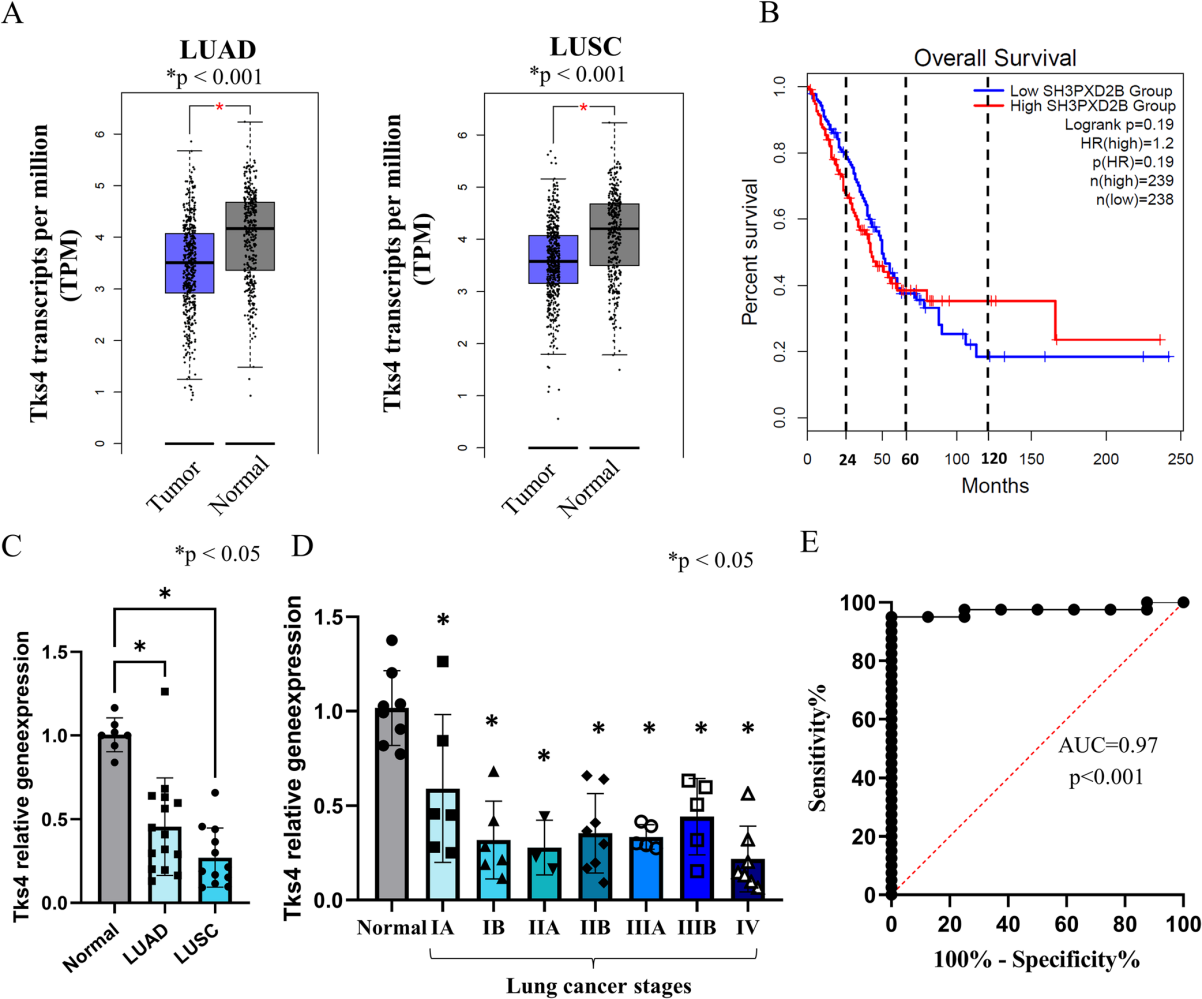
Analysis of Tks4 mRNA expression levels in human NSCLC samples using bioinformatic database tools and a patient-derived lung cancer tissue array. (A) Tks4 mRNA expression levels in LUAD and LUSC patient samples obtained from the GEPIA2 database (http://gepia2.cancer-pku.cn/#index). The expression levels are shown for normal lung tissues (*n* = 346 for LUAD, *n* = 337 for LUSC) and tumorous lung tissues (*n* = 483 for LUAD, *n* = 486 for LUSC). (B) Prognostic analysis of Tks4 gene expression on overall survival of patients with lung cancer using GEPIA2 database (http://gepia2.cancer-pku.cn/#index). The red line represents the “high Tks4 level group” (*n* = 239), while the blue line represents the “low Tks4 level group” (*n* = 238). The dashed lines indicate the survival rate at 2-year, 5-year, and 10-year of the analysis reflecting the differences in Tks4 level on survival curves. (C) Tks4 gene expression measured using a human lung cancer patients’ cDNA array. The expression levels are shown for normal lung tissue samples (*n* = 8), patients with LUAD (*n* = 15), and patients with LUSC (*n* = 12). (D) Analysis of Tks4 gene expression in lung cancer tissues from different disease stages. The array included cancer stages from I to IV. The classification of these stages is based on the size of the respective tumor, and the simultaneous involvement of lymph nodes and/or distant organs. The most aggressive stage of lung cancer is stage IV, which is metastatic lung cancer. The disease stages included in the array and the corresponding sample numbers were: IA (*n* = 6), IB (*n* = 6), IIA (*n* = 3), IIB (*n* = 8), IIIA (*n* = 5), IIIB (*n* = 5), and IV (*n* = 7). Expression levels were normalized to GAPDH. *p*-values were calculated using Student’s *t* test or ANOVA test with a Tukey post hoc test to compare the disease stages to the normal tissues. (E) ROC analysis of the results of the tissue cDNA. AUC: 0.97.

To further investigate the impact of Tks4 protein on human lung cancers and to assess whether Tks4 expression levels change during NSCLC development, we analyzed the levels of Tks4 mRNA in samples from patients with lung cancer using the GEPIA2 (Gene Expression Profiling Interactive Analysis) database, which is based on data from TCGA and GTEx (Genotype-Tissue Expression) (Tang *et al.*, 2019). This analysis revealed a significant decrease in Tks4 mRNA expression in tumorous lung tissues compared with normal lung tissue in both lung adenocarcinomas and lung squamous cell carcinomas (Figure 5A). Furthermore, the decreased level of Tks4 mRNA level was associated with poorer long-term overall survival in patients with lung cancer (Figure 5B).

To validate these in silico results, we performed a human cDNA array to measure the Tks4 mRNA levels in 48 human lung tissue samples, including healthy and NSCLC samples with documented cancer stages. The results from the human lung cancer array supported the database analysis findings, showing a decrease in Tks4 expression in lung cancer tissue compared with normal tissue (Figure 5C). Additionally, analysis of the lung cancer stages revealed a trend in which Tks4 levels were highest in normal tissue and lowest in more aggressive stage IV lung cancers (Figure 5D).

The low expression level of Tks4 observed in metastatic cancer patients further validated our Tks4-deficient lung cancer cell model for studying the role of Tks4 in EMT regulation.

To assess the potential usefulness of reduced Tks4 mRNA level as a biomarker, we performed receiver operating characteristic (ROC) curve analyses, which reveal the sensitivity and specificity of Tks4 expression levels in distinguishing normal and lung cancer tissue (Figure 5E). This analysis, performed on data from our lung cancer tissue cDNA array demonstrated that the Tks4 expression level serves as a highly specific and sensitive marker, as indicated by the area under the curve (AUC) value of 0.97 (Figure 5E).

### The link between the Tks4 interactome and regulation of epithelial plasticity in lung cancer cells

Our next goal was to uncover the Tks4-mediated molecular events involved in regulating the EMT-like process. Previous studies demonstrated that Tks4 is anchored to the plasma membrane through its PX domain and that it interacts with signaling molecules via its SH3 domain, establishing coordinated connections with proline-rich regions. We hypothesized that the multivalent properties of Tks4’s domain structure and its ability to bind various types of signaling proteins might enable the recruitment of not only invadopodia-regulating proteins but also potential EMT-regulating proteins.

**FIGURE 6:**
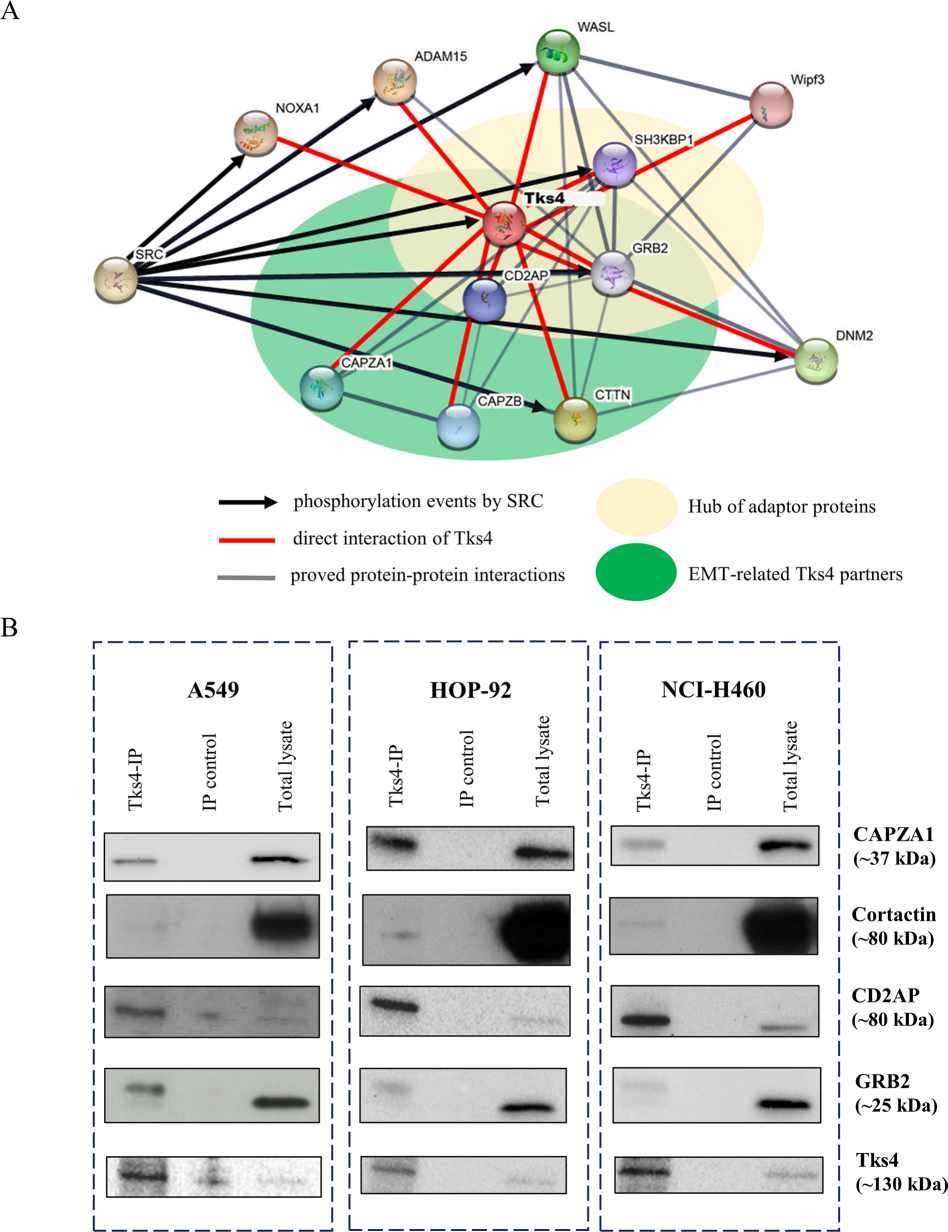
Protein–protein interaction analysis of Tks4 partner molecules: A Tks4 interaction network. (A) String based protein–protein interaction network analysis: Red lines indicate direct interactions of Tks4. Black arrows represent phosphorylation events on Tks4 interactors by SRC. Gray lines depict the interactions between other proteins in the network. Yellow ellipse represents a hub of adaptor proteins, while green ellipse represents EMT-related Tks4 partners. The interaction analysis was visualized using STRING (https://string-db.org/), incorporating our experimental data and literature search. (B) Tks4 interactions validation via Tks4-IP followed by WB in three lung cancer cell lines (A549: Lung adenocarcinoma; HOP-92: Lung non–small cell carcinoma; NCI-H460: Lung large cell carcinoma).

To gain a deeper insight into the diverse outcomes of Tks4-dependent signaling pathways, we surveyed the Tks4-binding partners described in the literature and performed Tks4 immunoprecipitation followed by mass spectrometry (Tks4-IP-MS) in five cancer cell lines (including A549 lung adenocarcinoma cells) to identify additional possible Tks4-associated EMT-related proteins. The results of the Tks4-IP-MS analysis are shown in Supplemental Table S4. In this analysis, we first listed all of the previously described and validated Tks4-binding molecules (Supplemental Table S5), including those identified in this study. Next, the Tks4 partner proteins were further analyzed via STRING to describe the Tks4-based signal interaction network (Figure 6A). Next, we confirmed the involvement of multiple proteins in the Tks4 interactome by immunoprecipitating endogenous Tks4 protein from different NSCLC lung cancer cell lysates (A549: human lung adenocarcinoma, HOP-92: human lung non–small cell carcinoma, NCI-H460: human lung large cell carcinoma) and performing WBs (Figure 6B). We discovered that while Grb2 and cortactin are present to a lesser degree in the Tks4-formed complex, CD2AP and CAPZA1 are abundant in the tested three lung cancer cell lines.

We then focused on the signaling proteins known to have documented effects on EMT events (Table 1). The protein–protein interaction analysis (Figure 6) revealed that Tks4 could form a protein complex with three adaptor proteins: SH3 domain-containing kinase-binding protein 1 (SH3KBP1/Cin85), CD2AP, and growth factor receptor bound protein 2 (Grb2). This scaffolding hub could serve as a docking platform for several EMT-related signal transducers (Table 1).

**TABLE 1:** List of Tks4-partner proteins involved in EMT regulation.

Tks4 partner protein	Protein type and function	EMT-related function
**CD2AP (CD2-associated protein)**	*Adaptor protein,* localizes cortactin and capping proteins at the cell periphery/membrane (Zhao *et al.*, 2013).	The CD2AP-Tks4 ratio drives the EMT process in colon cancer and HCT116 cells (Kurilla *et al.*, 2023).
**Grb2 (Growth factor receptor-bound protein 2)**	*Adaptor protein*, has a role in RAS pathway and actin-cytoskeleton rearrangement via the N-WASP–Arp 2/3 pathway (Carlier *et al.*, 2000).	GRB2 upregulation induces EMT in A549 cells by decreasing the expression of E-cadherin and increasing the expression of SNAIL (Mitra *et al.*, 2018).
**CAPZA1 (F-actin capping protein subunit alpha-1)**	*F-actin capping protein,* it forms a heterodimer with CAPZB, this dimer could bind to the barbed-ends of F-actin filaments to inhibit further polymerization (Zhao *et al.*, 2013).	Decreased expression of CAPZA1 protein promotes EMT and blocks the actin cytoskeleton rearrangement in hepatocellular carcinoma. The binding affinity of CAPZA1 to F-actin decreases in EMT-inducing hypoxic environments (Huang *et al.*, 2019).
**Cortactin/CTTN (Src substrate cortactin)**	*F-actin binding protein*, organized invadopodia/podosome formation and regulates actin filaments via Arp2/3 interaction (Weaver *et al.*, 2001).	Cortactin promotes EMT in melanoma, gastric cancer, and oral squamous cell carcinoma (Ji *et al.*, 2020).
**Src (Proto-oncogene tyrosine-protein kinase Src)**	*Tyrosine kinase*, which can phosphorylates several signaling proteins, e.g.,: cortactin (Huang *et al.*, 1998; Kourtidis *et al.*, 2017; Han *et al.*, 2019), Tks4 (Buschman *et al.*, 2009; Bögel *et al.*, 2012; Dülk *et al.*, 2018), Grb2 (Jones and Benjamin, 1997; Saci *et al.*, 2002; Wan *et al.*, 2003), SH3KBP1 (Narita *et al.*, 2005; Schroeder *et al.*, 2012; Kourtidis *et al.*, 2017), NOXA1 (Emerman *et al.*, 2010), ADAM15 (Poghosyan *et al.*, 2002; Kärkkäinen *et al.*, 2006; Zhong *et al.*, 2008).	Src promotes EMT by activating signaling pathways via phosphorylation events, leading to decreased E-cadherin expression, weakened cell-cell connection and by increasing mesenchymal marker expression, via phosphorylation events, e.g., in prostate cancer, breast cancer, pancreatic ductal carcinoma (Tatarov *et al.*, 2009; Liu and Feng, 2010; Drake *et al.*, 2012; Patel *et al.*, 2016; Nagathihalli *et al.*, 2012).

In addition to the scaffolding proteins, SRC kinase was also present in this protein interaction network. SRC kinase phosphorylates several Tks4-associated proteins, including cortactin, dynamin2, WASL, Grb2, SH3KPB1, NOXA1, and ADAM15 (Supplemental Table S5). It is known that tyrosine phosphorylation events by SRC activate signaling cascades that can inhibit or enhance the emergence of an EMT-like phenotype (Patel *et al.*, 2016).

Among the novel potential partner proteins identified via our MS analysis, CAPZA1 and CAPZB caught our attention due to the previously unexplored Tks4–CAPZ interaction. CAPZA1 and CAPZB form a heterodimer known as CAPZ that regulates the capping of the barbed end of actin filaments. Recent findings have revealed that CAPZ has a regulatory role in EMT-related events. Huang *et al.* demonstrated that CAPZA1 inhibits EMT by regulating actin cytoskeleton remodelling, and low levels of CAPZA1 promote EMT under low oxygen conditions in hepatocellular carcinoma cells (Huang *et al.*, 2019). Based on this knowledge and the fact that CAPZA1 was present in all five analyzed cell lines as a Tks4-interacting partner, we decided to study the potential interaction between Tks4 and CAPZA1 by validating the MS results (Supplemental Table S4.).

When attempting to identify possible interaction sites between the two proteins, we were primarily searched for short linear motif (SLiM)-mediated interactions. Although Tks4 has four SH3 domains through which most of its known interactions are mediated, the CAPZ heterodimer has no proline-rich disordered regions that could serve as ideal binding sites for SH3 domains; therefore, an SH3-mediated interaction mechanism is highly unlikely. However, we discovered that the long disordered region between the third and fourth SH3 domains of Tks4 contains a highly conserved region that is similar to the capping protein-interacting short linear motif (CPI; see http://elm.eu.org/elms/LIG_ActinCP_CPI_1). This motif is relatively long and has some generally conserved core positions as well as some protein family–specific positions that show considerable variability even among the validated partners of CAPZ. By performing a proteome-wide search for potential CAPZ interactors using only the core positions of the motif, the identified region (residues 636-654) of Tks4 scored very high (comparable to known CAPZ interactors), making Tks4 a very promising CAPZ interactor.

**FIGURE 7:**
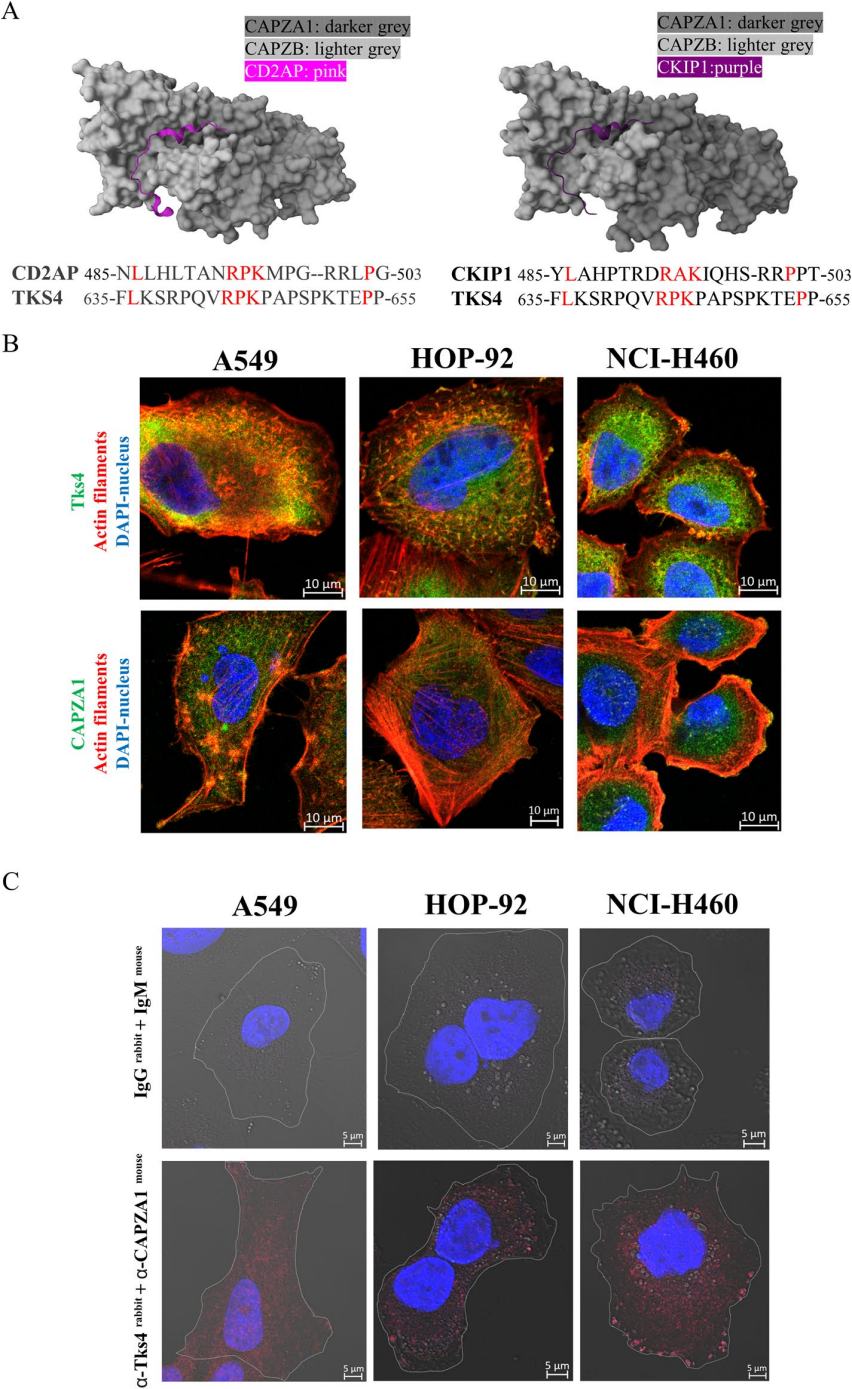
Analysis of the CAPZA1–Tks4 interaction. (A) Prediction of the CAP protein interaction motif in Tks4. Visualization of the CAP interaction motif in CD2AP cocrystalized with the CAPZ heterodimer (PDB ID:3AA6) and the CAP interaction motif in CKIP crystalized together with the CAPZ heterodimer (PDB ID:3AA1), as found in the PDB RCSB.org database (*https://www.rcsb.org/*). Alignment of the CAP interaction motif of CD2AP and CKIP to the predicted CAP interaction motif of Tks4. Conserved core residues of the motif are highlighted in red. (B) Visualization of Tks4 and CAPZA1 localization in three lung cancer cell lines. Tks4 or CAPZA1 (Alexa Fluor 488-green) were costained with F-actin (phalloidin-red) and captured via microscopy (confocal imaging; magnification: 63x). (C) Confirmation of Tks4-CAPZA1 binding in the three lung cancer cells using the Duolink PLA staining. The top row shows control samples stained only with secondary antibodies (anti-rabbit and anti-mouse). In the bottom row, anti-Tks4 (rabbit) and anti-CAPZA1 (mouse) antibodies were used to visualize the binding between the two proteins. Red dots indicate the interaction between the two proteins. Cell borders were visualized with white lines in the phase contrast images (63x).

It has long been known that capping protein regulators, for example, casein kinase 2-interacting protein-1 (CKIP1), CD2AP, capping protein Arp2/3 myosin I linker (CARMIL), and SH3KBP1/Cin85, bind to the stalk region of the CAPZ heterodimer through this motif (Figure 7A) (Drake *et al.*, 2012). The crystal structure of the CPI-like motifs of CD2AP and CKIP1 with the CAP heterodimer (Figure 7A) demonstrates these interactions. We aligned the sequence of the CPI-like motif derived from Tks4 with the CPI-like motifs of CD2AP and CKIP1. The alignments (Figure 7A) reveal that Tks4 contains the same or similar residues as the known interactors in the core positions of the CPI motif (positions in red in Figure 7A), while the flanking/intervening residues that would not be constrained by the functionality of the motif show low similarity. This island-like conservation pattern is a characteristic feature of linear motifs that independently appeared in unrelated proteins through evolution (Davey *et al.*, 2015), therefore, these findings strongly support that the identified motif in Tks4 is a true functional module enabling CAPZ binding. Based on this analysis, we propose that the CPI-like motif in Tks4 represents the primary interaction site and could potentially bind to the stalk region of the CAPZ heterodimer, similar to the binding of other CPI motifs identified in capping protein regulators (Figure 7A).

Next, we validated and visualized the Tks4-CAPZA1 interaction in A549, HOP-92, and NCI-H460 lung cancer cell lines cells (Figure 7B). First, we stained Tks4 or CAPZA1 with F-actin, which showed in general that both Tks4 and CAPZA1 are present at the membrane and the cytoplasm. Particularly for Tks4 detection, we found abundant expression and its colocalization with membrane structures resembling actin-rich invadopodia. When CAPZA1 localization was studied, the CAPZA1 staining pattern showed a typical small punctate distribution in the cells. This suggests that CAPZA1 attaches to the barbed ends of actin that are dispersed throughout the cytoplasm. Proximity ligation assay (PLA) was also performed to confirm that the two proteins are associated in cells, not only in cell lysates. The result of Tks4 and CAPZA1 double staining in fixed cells was shown on Figure 7C, demonstrating the close proximity and binding of the two proteins in the cytoplasm of A549, HOP-92, and NCI-H460 lung cancer cell lines cells.

**FIGURE 8:**
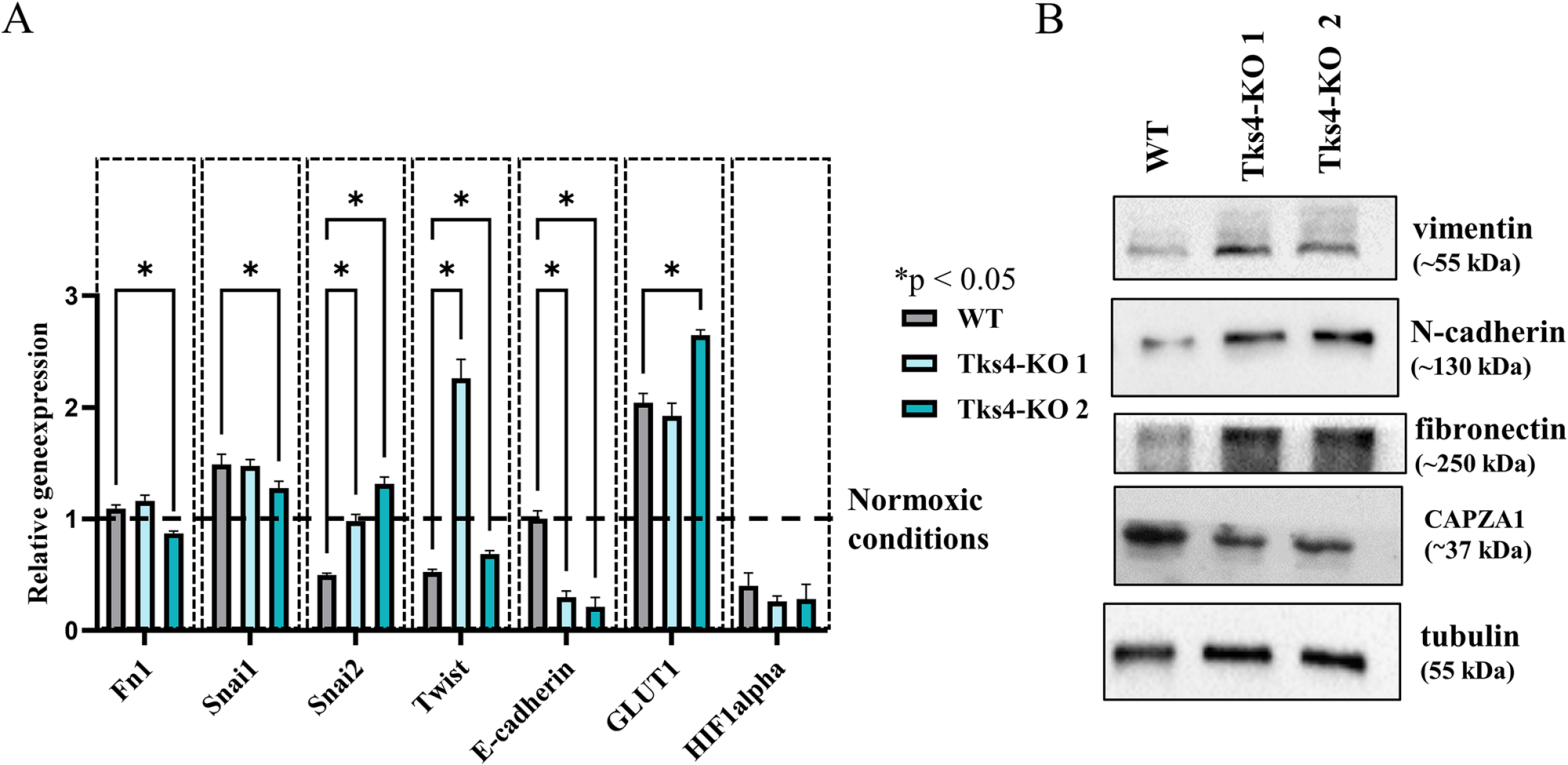
Effect of low oxygen concentration treatment/condition on the EMT-like phenotype in lung cancer cells. (A) Measurement of EMT markers (Fn1 = fibronectin, Snai1, Snai2, Twist) and the low oxygen condition indicator markers GLUT1 and HIF1alpha expression via qRT-PCR after low oxygen treatment (3% oxygen) for 3 d. Data were normalized to the normoxic control samples, indicated with dashed line at the level of 1. (B) Measurement of EMT markers (vimentin, N-cadherin, fibronectin) and CAPZA1 expression levels by WB. Tubulin was used as a loading control.

To further investigate the effect of Tks4 knockdown on CAPZA1 and its role in EMT induction, we employed a similar experimental setup as was used to study the role of CAPZA1 in hepatocellular carcinoma cells (Huang *et al.*, 2019). Accordingly, lung cancer cells were subjected to low oxygen (3%) for 3 d to create an EMT-supporting environment. The overall effect of the low oxygen condition was validated by measuring the increased expression of GLUT1 (glucose transporter 1) and the decreased expression of HIF1alpha in the samples subjected to low oxygen (Figure 8A; Supplemental Figure S3) (Zhang *et al.*, 1999; Cavadas *et al.*, 2015). We observed that the low oxygen level led to a higher level of Snai2 mRNA (Figure 8A). At the protein level, we examined changes in vimentin, N-cadherin, and fibronectin expression and observed increases in the Tks4-KO cell lines compared with the WT cells (Figure 8B). These findings indicated that the Tks4-KO cell lines exhibited a more pronounced EMT-like state than did the WT cells and displayed a faster response to the EMT-inducing low oxygen condition (Liu *et al.*, 2018). Furthermore, the CAPZA1 level is also downregulated after Tks4 knockdown during the EMT process under low oxygen conditions (Figure 8B), suggesting potential coregulation between CAPZA1 and Tks4 during EMT.

In this study, we identified a novel Tks4-interacting protein that has a documented effect on an EMT-like process and is part of the extensive network of Tks4-associated proteins outlined in Figure 5.

## DISCUSSION

The initiation of the EMT process within a population of cancer cells is a landmark event in the progression towards metastatic and aggressive tumors. It involves the activation of EMT-related transcription factors and the redistribution and accumulation of cytoskeletal remodeling-associated proteins in a complex beneath the plasma membrane. The EMT process occurs to varying degrees within individual cancer cells, resulting in a heterogeneous population that coexpresses epithelial and mesenchymal markers. As a result, EMT represents a wide range of stages in which the epithelial characteristics of certain adenocarcinoma cells are gradually transitions into a mesenchymal-like phenotype.

In this study, our focus was on investigating the role of Tks4 in regulating EMT in lung cancer. The notion that Tks4 influences the EMT-like process emerged from recent publications. For instance, it was demonstrated that the lack of Tks4 induces an EMT-like phenotype in colon cancer cells (Szeder *et al.*, 2019; Jacksi *et al.*, 2024). Furthermore, Kui *et al.* explored the prognostic value of Tks4 in hepatocellular carcinoma via a combined multiomics and experimental study (Kui *et al.*, 2021).The potential involvement of Tks4 in metastasis was also reported in a study on lung metastasis-forming melanoma cells in mice (Iizuka *et al.*, 2016). Given these findings, we aimed to explore the effect of Tks4 in a cancer type that has not been extensively studied in this context. We selected primary lung adenocarcinoma cells to extend our research on Tks4 by investigating the impact of Tks4 KO as well as the prognostic value of its expression level in NSCLC. To this end, we chose the A549 cell line, a well-established in vitro lung cancer model, and used CRISPR/Cas9 to mutate the Tks4-encoding gene in this cell line. We successfully generated two Tks4-KO clones and assessed the effects of Tks4 KO on EMT-related events.

The lack of Tks4 resulted in an elongated mesenchymal-like phenotype in lung cancer cells, similar to that observed in Tks4-KO colon cancer cells, which exhibit elongated soma and decreased circularity (Szeder *et al.*, 2019). Furthermore, our findings revealed an epithelial/mesenchymal hybrid phenotype in Tks4-KO lung cancer cells characterized by increased gene expression levels of mesenchymal markers, for example, fibronectin, N-cadherin, and Snail2 transcription factor, while the expression of the epithelial marker E-cadherin was decreased in the Tks4-KO clones (Figure 4A). It is important to note that not all EMT-associated protein levels were uniformly altered, as N-cadherin remained at the WT level, as observed via ICC and WB experiments.

In general, the migration and invadopodia formation ability are the hallmarks of invasive cancer phenomena. Interestingly, the migration ability of the Tks4-KO cancer cells was slightly reduced both in nontreated and EGF-treated cells. This finding can be explained by the fact that Tks4 has a well-defined role in the EGFR/SRC pathway; therefore, the lack of this protein can lead to impaired EGFR signaling and consequential reduced cell motility. As previously mentioned, Tks4 is a key factor in invadopodia formation, therefore it is not surprising that in the absence of Tks4 the invadopodia formation of the cells was impaired. Based on the findings that mesenchymal phenotypic and EMT marker alterations are associated with reduced migration and invadopodia formation in Tks4-KO cells, we conclude that Tks4 may control the gradual shifts of the EMT process where mesenchymal and epithelial characteristics coexist.

It was previously shown that under hypoxic conditions, the EMT shift can be accelerated in WT A549 cells (Liu *et al.*, 2018). In our experiments, the low oxygen concentration had a more robust EMT-enhancing effect on Tks4-KO cells than on WT cells, as indicated by increased expression levels of mesenchymal markers, including vimentin, N-cadherin, fibronectin, and Snai2 compared with the WT cells. As the Tks4-KO cells responded to the low oxygen stress with an enhanced EMT-like phenotypic change than WT A549 cells, these results also show that the lack of Tks4 resulted in an EMT-prone stage, and Tks4 has a potential role in regulating the initial steps of EMT.

In addition to the cancer cell line study, we also wanted to measure the expressional changes of Tks4 during human lung cancer development. Furthermore, although Tks4’s applicability as cancer biomarker in stomach and hepatocellular cancer has already been shown, the relevance of Tks4 in lung cancer has not been explored. Thus, database analysis and a human lung cancer tissue array measurement were also applied in addition to the wet-lab in vitro experiments. The results provided further details on changes in Tks4 expression level during NSCLC progression, as we generally observed that the Tks4 mRNA level is lower in lung cancer tissues compared with healthy tissues (Figure 5, A and C), suggesting its potential as a biomarker for distinguishing between healthy and cancerous lung tissues in patients (Figure 5E). Furthermore, the lowest expression level of Tks4 was measured in the most advanced stage of NSCLC (Figure 5D). Accordingly, the analysis of lung cancer patient survival analysis data showed that a decrease in Tks4 level indicates poorer long-term overall survival after approximately 60 mo (Figure 5B). Therefore, measuring the Tks4 mRNA expression level in surgically removed lung tissue samples via qPCR may help to determine the possible prognosis of the disease. Importantly, the effect of high or low Tks4 expression levels on cancer patient survival is cancer-type dependent. In case of hepatocellular carcinoma the higher levels of Tks4 mRNA expression are associated with shorter survival periods in contrast to lung cancer (Kui *et al.*, 2021). We assume that Tks4 might have a different role depending on the cancer cell type or the cancer stage in tumor progression due to its involvement in multiple signaling pathways ranging from actin rearrangement and EMT-like process regulation. Considering that EMT is a hallmark of lung cancer progression (Legras *et al.*, 2017), we concluded that the reduced Tks4 level in NSCLC samples possibly supports the importance of EMT-promoting signalisation in patients, most likely in a manner similar to that of in vitro the Tks4-KO lung cancer cell line.

The observed EMT-like phenotype in the Tks4-KO lung cancer cells piqued our interest, as the well-studied role of Tks4 in podosomes/invadopodia formation suggests the opposite outcome in the EMT process in the absence of Tks4. The mechanism behind these different effects in the absence of Tks4 could reflect the fact that its partners also have multiple effects on EMT. For instance, it is widely documented that the Tks4 partner molecule cortactin is also involved not only in organizing invadopodia (Lányi *et al.*, 2011), but it can promote EMT (Ji *et al.*, 2020); however, Lee *et al.* presented evidence that the EMT-promoting transcription factor Snail1 could repress cortactin expression and impair its actin reorganizing function (Lee *et al.*, 2014). Consistent with this, Tks4 KO resulted in decreased cortactin/actin colocalization, leading to reduced invadopodia formation (Figure 2, A and B). Nonetheless, Tks4 loss concurrently produced supporting effects on other EMT-like changes.

Multiple pieces of evidence indicate the involvement of other Tks4 partner molecules in the regulation of the EMT-like process. Importantly, in addition to the Tks4–cortactin interaction, the Tks4-CD2AP complex is also relevant in the regulation of the EMT-like process. CD2AP is an adaptor protein that is mainly involved in dynamic actin assembly-related pathways (Mustonen *et al.*, 2005). In Kurilla *et al.* we demonstrated that the expression ratio of Tks4 to CD2AP in human colon cancer cells determines the outcome of EMT upregulating or downregulating signals and has a diagnostic role in colon cancer (Kurilla *et al.*, 2023).

Nevertheless, our protein–protein interaction analysis also revealed that the Grb2 adaptor protein is also linked to the Tks4-recruited protein complex (Figure 6). Previous studies have shown that Grb2 can modulate EMT via the Snail transcription factor (Mitra *et al.*, 2018) and via the SHP2/Grb2/PI3K/AKT signaling cascade in lung cancer (Wang *et al.*, 2022). The involvement of Grb2 in EMT modulation represents another player in the Tks4-organized network that regulates EMT.

As we explored Tks4’s interactions with adaptor proteins (i.e., SH3KBP1, CD2AP, Grb2) and effectors involved in EMT regulation, it became clear that the SRC tyrosine kinase is also part of the Tks4 signalome. SRC belongs to the EGFR signaling pathway and also regulates EMT events via phosphorylation (Patel *et al.*, 2016). Studies on the interaction between Tks4 and SRC have shown that Tks4 anchors SRC to the plasma membrane and serves as a docking site for EGFR and SRC (Bögel *et al.*, 2012; Dülk *et al.*, 2018). Moreover, several molecules that bind to Tks4 (Supplemental Table S5) are SRC kinase substrates, further increasing the complexity of the regulation within this protein network (depicted by the black arrows in Figure 6).

Grb2 is also a member of the EGFR/SRC pathway (Wee and Wang, 2017). As Grb2 recruits proteins to the EGFR and AKT pathways and simultaneously modulates cell motility and EMT-related events, it is possible Tks4 could also affect signaling via Grb2 and SRC, thereby regulating various cellular processes with different outcomes. Therefore, we propose that the reduced Tks4 expression in lung cancer cells might disrupt the interactions or cellular localization of specific partner molecules, potentially leading to enhanced EMT initiation.

In addition to analyzing Tks4-related EMT regulatory molecules individually, it is apparent that the protein partners of Tks4 also directly interact with each other, forming an interconnected network (Figure 6). Among these complex relationships, the binding between cortactin and CD2AP serves as a key mediator of actin cytoskeleton assembly during cell movement by transmitting signals to WASP and dynamin2 (Bandela *et al.*, 2022). Additionally, the adapter proteins involved in this complex exhibit similar binding partners. For example, CAPZA1 and cortactin can directly bind to both Tks4 and CD2AP molecules (Hutchingst *et al.*, 2003; Lynch *et al.*, 2003).

The notion that Tks4 recruits proteins with a dual role in cytoskeleton rearrangement and the EMT-like process was further supported by the identification of CAPZ proteins as novel Tks4 interactors via our Tks4-IP-MS analysis (Supplemental Table S4). The association of these proteins is detected in three lung cancer cell lines using the Tks4-IP-WB and PLA method, increasing the confidence of the MS-based presence of Tks4-CAPZA1 binding (Figures 6B and 7C). The predicted binding sites for this interaction are the capping protein-interacting motif in the disordered region of Tks4 and the stalk region of the CAPZ heterodimer (Figure 7A). Further structural analysis using designed Tks4 mutants is required to study the specific structural requirements for this interaction and its functional implications. Based on our results, we conclude that these proteins interact A549 cells (Figure 5) and potentially exert EMT-regulating effects.

The CAP heterodimer, composed of CAPZB and CAPZA1 proteins, functions in actin filament elongation by capping the barbed ends of growing actin filaments (Santio *et al.*, 2020). Low CAPZA1 expression levels have been associated with increased invasive phenotypes in gastric cancer (Lee *et al.*, 2013) and lung cancer (Wei *et al.*, 2023). Consistent with this, CAPZA1 overexpression inhibited the EMT process in hepatocellular carcinoma (Huang *et al.,* 2017). Moreover, in hepatocellular carcinoma cells, it was demonstrated that decreased expression of CAPZA1 drives the low oxygen-induced EMT process (Huang *et al.*, 2019). Our study further supports these observations as we observed an EMT-like phenotype in Tks4-KO A549 cells under low oxygen conditions (Figure 8, A and B), accompanied by lower CAPZA1 expression (Figure 8B) compared with WT cells. One possible explanation for this phenomenon is that the lack of interplay between Tks4 and CAPZ proteins in Tks4-KO cells modulates EMT-inducing signaling pathways under low oxygen conditions (Figure 8). However, further detailed analysis is needed to explore this notion.

Literature analysis of the Tks4-signalome revealed that CAPZ is also bound and regulated by the CD2AP adaptor protein, which is a direct interactor of Tks4 (Figure 6), further contributing to the complexity of EMT regulatory signaling within this protein network (Xie *et al.*, 2020). Intriguingly, CD2AP is one of the highest affinity interactors of CAPZ among its known regulators (McConnell *et al.*, 2020). If there is any competition or cooperativity between CD2AP and Tks4 binding to CAPZ and how it might influence EMT, and cell motility is yet to be elucidated.

The results discussed above suggest that the assembly of the Tks4 signalome (Figure 6) and the actual presence of the participant molecules within the Tks4-organized network cooperate to modulate the EMT-like process and the invadopodia formation. We suspect that within the Tks4 interactome, the signaling molecules can exert different effects on EMT regulation and also on controlling actin rearrangements. Therefore, the final outcome of the entire pathway is highly context dependent. In the absence of Tks4, we have observed EMT-like events but at the same time the deregulation of invadopodia formation, as two seemingly opposing events. Furthermore, the low oxygen experiments demonstrated that when Tks4 was absent, the level of the interacting protein CAPZA1 was also reduced, which in turn leads to enhanced EMT-like state.

We consider that the loss of Tks4 disrupts several cellular processes, this is because Tks4 can influence EMT processes, migration, as well as invadopodia formation, depending on the actual combination of their effector binding partners. We assume that Tks4 might have multiple effects on tumor progression due to its position as a central scaffold protein, and that depending on the actual assembly of the Tks4-interactome members can either fuel or repress these pathways.

Alternatively, considering the complex relationship between the tumor cells and the microenvironment, the controversies of the outcome of Tks4 deregulation will likely be resolved by separate biological analysis of the lung cancer cells with different cancer stage background from various other components of the tumor microenvironment.

Taken together, this paper significantly advances our knowledge on the Tks4 interactome itself and its role in regulating the EMT-like process in lung cancer cells. Our findings suggest that the expression level and protein interactions of Tks4 are important parameters controlling EMT.

## MATERIALS AND METHODS

Request a protocol through *Bio-protocol*.

### Cell line and cell culture

The A549 cell line was generously provided by Dávid Szüts’ research group. The cells were cultured in a humidified chamber at 37°C with 5% CO_2_. F12K medium (Thermo Fisher Scientific, Life Technologies, catalog no.: 21127022) supplemented with 10% FBS (Thermo Fisher Scientific, Life Technologies, catalog no.: 16000044) and 0.5% Penicillin (10,000 units)/Streptomycin (10 mg) (Merck, Sigma-Aldrich, catalog no.: P0781) was used for cell culture. Prior to the experiments, karyotyping analyses were performed by UD-GenoMed Medical (Supplemental Figure S1A). Subsequently, the A549 cells were used to generate a Tks4-KO cell line using the CRISPR-Cas9 genome editing method. The authenticity of the WT A549 cells and Tks4-KO cells was confirmed using DNA profiling with specific short tandem repeats markers (Supplemental Figure S1B), and the cells were tested for *Mycoplasma* contamination (Microsynth AG) (Supplemental Figure S1A).

For the low oxygen concentration experiment, the cells were placed in a hypoxic chamber with 3% O_2_ for 3 d. Following the exposure to low oxygen, RNA and protein were isolated from the cells for subsequent qPCR and WB analyses.

For the MS interactome analyses, we used the A549 and four other cell lines (HCT116, MCF7, HPAC, N87) to validate the CAPZA1–Tks4 interaction. All cell lines were handled for a short period of the MS measurement according to the manufactural instruction as indicated by A549 cell line culturing procedure. For HCT116 McCoy’s 5A medium (Thermo Fisher Scientific, Life Technologies, catalog no.: 26600023), for MCF7 DMEM (Thermo Fisher Scientific, Life Technologies, catalog no.: 11960085), for HPAC DMEM-F12 medium (Thermo Fisher Scientific, Life Technologies, catalog no.:31330095) and for N87 RPMI 1640 medium (Thermo Fisher Scientific, Life Technologies, catalog no.:72400054) was used and all medium was supplemented with 10% FBS (Thermo Fisher Scientific, Life Technologies, catalog no.: 16000044) and 0.5% Penicillin (10,000 units)/Streptomycin (10 mg) (Merck, Sigma-Aldrich, catalog no.: P0781).

For the IP-WB, ICC, and PLA measurements, we have used lung cancer cell lines (HOP-92: human lung non–small cell carcinoma, NCI-H460: human lung large cell carcinoma).The cell lines, generously provided by András Füredi’s research group and handled for a short period of these measurements according to the manufactural instruction as indicated by HOP-92, NCI-460 cell line culturing procedure. The cells were cultured in a humidified chamber at 37°C with 5% CO_2_. RPMI 1640 medium (Thermo Fisher Scientific, Life Technologies, catalog no.:72400054) supplemented with 10% FBS (Thermo Fisher Scientific, Life Technologies, catalog no.: 16000044) and 0.5% Penicillin (10,000 units)/Streptomycin (10 mg) (Merck, Sigma-Aldrich, catalog no.: P0781) was used for cell culture.

### CRISPR-Cas9 genome editing to create Tks4-KO A549 cell line

A549 cells were transfected with pCMV-Cas9-GFP_SH3PXD2B (Merck, Sigma-Aldrich) using FUGENE transfection reagent (Promega, catalog no.: E2311). After 24 h, GFP-expressing cells were sorted by FACS (Attune FACSARIA III sorter) at a density of 1 cell per well onto a 96-well tissue culture plate to allow for clonal expansion. A total of 24 clones were recovered for screening for Tks4 mutations. The potential Tks4-KO clones were genotyped using the method described in our previous publication (László *et al.*, 2022). Sanger sequencing, ICC, and WB analysis were performed to confirm the absence of Tks4 in the selected clones. The reference (WT) Tks4 sequence was downloaded from NCBI GenBank: https://www.ncbi.nlm.nih.gov/genbank/ (Homo sapiens SH3 and PX domains 2B [SH3PXD2B], transcript variant 1, mRNA: NCBI Reference Sequence: NM_001017995.3).

### Cell viability and proliferation assay

In triplicate, 2500 cells were seeded into each well of a 96-well plate. Cell proliferation of the WT and Tks4-KO cells was assessed using the Cell Proliferation Kit I (MTT: (3-(4,5-DIMETHYLTHIAZOL-2-YL)-2,5-DIPHENYLTETRAZOLIUM BROMIDE; Merck, Roche, catalog no.: 11465007001). First, MTT labelling reagent was added to each well containing the cells, and the plate was incubated for 4 h at 37°C in a humidified chamber with 5% CO_2_. After the incubation period, solubilization buffer was added to the cells and the plate was incubated overnight. The next day, the absorbance at 600 nm was measured using a microplate reader. A reference wavelength at 700 nm was also measured and the corresponding values were subtracted. Cell numbers were determined at 24 h, 48 h, and 72 h based on the absorbance measurements.

### Cell shape quantification

For cell shape quantification and statistic measurement, we used the form factor analysis on the WT and the two Tks4-KO cell lines. The following equation defines Form factor (FF): By using FF = 4π(area)/(perimeter)^2^, a value between 0 and 1 can be generated, where a circular cell shape is indicated by values closer to 1. To see alterations in shape and the cytoarchitecture, phalloidin, an F-actin dye, was utilized. The cells’ area and perimeter length were then measured, and the ratio between the two values was determined (Szebényi *et al.*, 2023). To do this, we analyzed 55 images (110 cells) per each cell type generated from eight repeated experiment using Image J.

### RNA extraction and qRT-PCR

Total RNA was isolated from the cells using TRIzol Reagent (Life Technologies, Ambion, catalog no.:15596026) and the Direct-zol RNA Miniprep Kit (Zymo Research, catalog no.: R2052). The concentration of RNA was determined using a Nanodrop spectrophotometer. For cDNA synthesis, 300 ng of total RNA was reverse transcribed using the First Strand cDNA synthesis kit for RT-PCR (Merck, Roche, catalog no.: 11483188001). For qPCR analysis, Taqman hydrolysis probes (Thermo Fisher Scientific) were used for the following genes: Fn1, Snai1, Snai2, Twist, E-cadherin, GAPDH, GLUT1/SLC2A1 (Supplemental Table S1). The TaqMan Fast Advanced Master Mix (Thermo Fisher Scientific, catalog no.: 4444557) was used for qPCR amplification. Alternatively, gene-specific primers and SyberGreen master mix (Thermo Fisher Scientific, catalog no.: 4368577) were used for E-cadherin, Zeb1, Vimentin, N-Cadherin, and GAPDH. The qPCR reactions were performed on an Applied Biosystem Quantstudio 6 pro instrument to measure the gene expression levels.

In each qPCR measurement, GAPDH was used as a housekeeping gene for normalization, and the 2^–ΔΔCt^ method was used to calculate the relative expression levels.

### Cell lysis and WB

A549 cells were cultured in a 100 mm Petri dish and harvested to extract total proteins by cell scraping using lysis buffer. The lysis buffer contained 50 mM HEPES buffer (pH 7.4), 100 mM NaCl, 1% Triton X-100, 20 mM NaF, 1 mM EGTA, 1 mM Na_3_VO_4_, 1 mM p-nitrophenyl-phosphate, 10 mM benzamidine, 1 mM phenylmethylsulfonyl fluoride, and 25 μg/ml each of leupeptin, soybean trypsin inhibitor, and aprotinin. Cell debris was removed from the protein lysates by centrifugation at 20,800 rpm for 10 min at 4°C. The protein concentration was determined using Lowry reagents (Bio-Rad, DC Protein Assay Kit I catalog no.: #5000111) and a microplate reader.

To prepare the protein lysates for gel electrophoresis (SDS–PAGE) (and later on for the Tks4-IP-MS measurement, as well), 4x sample loading dye (composed of water, Tris pH 6.8, glycerin, SDS, β-mercaptoethanol, and bromophenol blue) was added to the lysates, followed by incubation at 100°C for 5 min. Equal amounts of protein (20 μg) from each sample were loaded into the wells of 10% stain-free gels (Bio-Rad, TGX Stain Free FastCast Acrylamid Kit, catalog no.: 1610183). SDS–PAGE was performed at 160 V for 45 min at room temperature (RT). The stain-free blots were imaged using the Chemidoc Stain-Free Blot (Bio-Rad) function to verify protein concentrations.

The protein samples were then transferred to nitrocellulose membranes using either a 1-h, 300 mA transfer at 4°C or an overnight, 25 V transfer at 4°C. The membranes were blocked for 60 min with 5% skimmed milk in PBS and then incubated overnight at 4°C with the appropriate primary antibodies: vimentin (rabbit polyclonal, Abcam: ab137321), E-cadherin (rabbit polyclonal, Abcam: ab15148), N-cadherin (mouse monoclonal, Thermo Fisher Scientific: #33-3900), fibronectin (rabbit polyclonal, Abcam: ab45688), Tks4 (rabbit polyclonal [Lányi *et al.*, 2011]), and CAPZA1 (mouse monoclonal, Santa Cruz Biotechnology: sc-374302, mouse monoclonal, Invitrogen: MA5-36109), HIF-1-ALPHA (rabbit monoclonal, Abcam: ab179483), Cortactin (mouse monoclonal, Santa Cruz Biotechnology: sc-55579), GRB2 (mouse monoclonal, Sigma-Aldrich: 05-372), Tks5 (mouse monoclonal, proteintech: 18976-1-AP), CD2AP (mouse monoclonal, Invitrogen: MA5-33009) (dilutions in Supplemental Table S2). Most of the membranes were cut before antibody hybridization to allow multiple proteins to be tested simultaneously due to the small sample size. Same blots were probed for multiple proteins, full-length blots are presented in Supplemental Figure S2. The primary antibodies were diluted in 1% skimmed milk with Tween 20 in PBS.

After several washing steps with 1% milk with Tween 20 in PBS (5 min per wash, RT), HRP-conjugated secondary antibodies (either mouse: Merck, Sigma-Aldrich: A6782 or rabbit: avantor by vwr: NA934) were added to the membranes and incubated for 1 h at RT. The blots were then washed three times for 10 min with 1% milk with Tween 20 in PBS at RT, followed by a final wash in PBS for 5 min at RT. The blots were detected using enhanced chemiluminescence (ECL) reagents (Amersham, catalog no.: RPN2134) and visualized using either the ChemiDoc MP system (Bio-Rad) or an x-ray detection device. The loading control used in each experiment was alpha-Tubulin (mouse monoclonal, Merck, Sigma-Aldrich: T6199).

### ICC and confocal microscopy

A549 cells were cultured in a 12-well removable ibidi chamber (ibidi, catalog no.: 81201) at a density of 5000 cells per well. The cells were then fixed with 4% paraformaldehyde (PFA) for 15 min at RT. After washing with Dulbecco’s phosphate-buffered saline (DPBS, Merck, Sigma-Aldrich, catalog no.: D8537) the cells were permeabilized with 0.1% Triton X-100 in sterile DPBS for 10 min at RT. Next, the cells were blocked with 5% BSA in sterile DPBS for 1 h at RT to reduce nonspecific binding. The appropriate primary antibody (vimentin [rabbit polyclonal, Abcam: ab137321], N-cadherin [mouse monoclonal, Thermo Fisher Scientific: #33-3900], fibronectin [rabbit polyclonal, Abcam: ab45688], Tks4 [rabbit polyclonal (Lányi *et al.*, 2011)], CAPZA1 [mouse monoclonal, Santa Cruz Biotechnology: sc-374302], Cortactin [rabbit polyclonal: Santa Cruz Biotechnology: sc-11408], Tks5 [mouse monoclonal, proteintech: 18976-1-AP] dilutions in Supplemental Table S2), was then added to the cells and incubated overnight at 4°C. After several washing steps with DPBS, the cells were incubated with mouse (Alexa488, catalog no.: A-11029) or rabbit (Alexa488, catalog no.: A-11008/Alexa546, catalog no.: A11035) Alexa Fluor-labeled secondary antibodies (Thermo Fisher Scientific) and CF543 Phalloidin (Biotium, catalog no.: 00043) to detect F-actin (dilutions in Supplemental Table S2). The cells were incubated with these secondary reagents for 1 h at RT. To visualize the cell nuclei, DAPI (Thermo Fisher Scientific, catalog no.: 62248) was added and incubated for 10 min at RT. Following the final washing step with DPBS (3 min, RT), the silicone chamber was removed, and the slide was mounted with FluorSave Mounting medium (Merck, Millipore, catalog no.: 345789) using a coverslip. Then the edges of the slides were sealed with nail polish. Imaging was performed using a Zeiss LSM-710 confocal microscopy system with a 20x–63x objective. The acquired confocal images were analyzed using either ZEN software or ImageJ software for further analysis.

### Wound healing assay

For this experiment, a culture insert 24-well plate (ibidi, catalog no.: 80242) was utilized. Each well contained a silicone gasket with two 70 µl compartments, separated by a silicone wall. The silicone wall created a wound area after the gasket was removed. To begin, 66,000 cells were seeded into each 70 µl compartment. After an overnight incubation, the medium was replaced with serum-free medium and incubated overnight again. Once the wells reached confluence the following day, the silicone gasket was carefully removed using sterile forceps. The wells were then washed twice with sterile DPBS. Each cell type (WT, Tks4-KO1, Tks4-KO2) had four wells assigned to it. Two of the wells were incubated in 2% FBS F12K medium, while the other two wells were treated with 50 ng/ml EGF. Cell migration was recorded for 24 h at 15-minute intervals using a JuLI stage real-time live cell imaging system. Two fields were captured from each well using a 4x objective. The obtained images were analysed using Image J software, specifically utilizing the Wound Healing Size Tool plugin, to measure the wound healing area over time.

### Tissue cDNA array

To investigate the expression level of the Tks4 gene in NSCLC (specifically, lung adenocarcinoma [LUAD] and lung squamous cell carcinoma [LUSC]), the GEPIA2 database (http://gepia2.cancer-pku.cn/#index) was utilized. The cut-off value was determined based on median levels, and the setup parameters were a *p*-value cutoff of 0.001 and a log_2_FC cutoff of 0.6. Additionally, survival analysis was performed using GEPIA2.

The gene expression levels of Tks4 and GAPDH (used as a housekeeping control) (Thermo Fisher Scientific, Taqman probes: Supplemental Table S1) were measured using the TissueScan Lung Cancer cDNA Array III (Origene, catalog no.: HLRT103) as in the following articles (Jiang *et al.*, 2014; Wu *et al.*, 2019). The Lung Cancer cDNA Array is commercially available in a ready-to-use array format, all the (normal and cancerous) human samples on the array were collected, processed, and sold by Origene.

The array consisted of a 96-well plate with 48 samples, including eight normal samples and various stages of lung cancer (stage IA *n* = 6, IB *n* = 6, IIA *n* = 3, IIB = 8, IIIA = 5, IIIB = 5, IV = 7). The 2^–∆∆Ct^ method, as previously mentioned in the qRT-PCR section, was utilized to calculate the relative expression levels (Supplemental Table S3).

### Interactome analyses

The protein–protein interaction analysis was conducted using the STRING database (https://string-db.org/). Interactions with an interaction score above 0.40 were considered, excluding text mining results. This analysis was supplemented with literature search and our experimental data to identify relevant protein interactions associated with Tks4.

### IP

The cell lysates from different cell lines (HCT116, MCF7, A549, HPAC, N87, HOP-92, NCI-H460) were prepared using the same protocol as described by the first steps of WB. Then Protein Sepharose A beads (Sigma-Aldrich, catalog no.: P9424) were placed to 1.5 ml tubes and the beads were then centrifuged and washed three times with cold DPBS (4°C, 20,800 × *g*, 10 min). Following the washes, Tks4 antibody and the protein lysates were added separately to the beads. The solutions were then rotated for 1 h at 4°C to allow for antibody-protein binding. After the rotation step, the beads were washed four times with 0.1% Triton X PBS solution (1 min, 17,800 × *g*). Following the final wash step, the beads were resuspended in sample loading dye (4x), and the tubes were boiled for 5 min at 100°C to denature the proteins. Subsequently, either WB analysis or SDS–PAGE followed by MS was performed to detect and analyse the proteins bound to endogenous Tks4.

### MS

After the IP and SDS–PAGE, the gel was stained with Coomassie blue to visualize the proteins. The bands of interest were then excised from the gel based on their molecular weight ranges (10–25 kDa, 25–50 kDa, 50–100 kDa, 100–300 kDa). These gel slices were sent to UD-GenoMed Medical Genomic Technologies Kft (Debrecen, Hungary) for MS analysis. As a negative control, IP beads were incubated with protein lysates without adding the primary antibody. This control was used to ensure that any proteins identified in the IP samples were specifically bound to the primary antibody.

The results obtained from the MS measurements from five cell lines were analyzed using Scaffold viewer software. Protein threshold was set to 99%, minimum peptide count was set to 3, and the false discovery rate was set to 0.1% to filter and identify the proteins present in the IP samples.

### Identification of potential binding sites between Tks4 and CAPZ

We looked for a possible SLiM-mediated interaction between Tks4 and the CAPZ heterodimer. Although Tks4 has four SH3 domains, their specific recognition motifs have not yet been described. However, SH3 domains usually bind proline-rich motifs within disordered regions and CAPZ does not have disordered regions and has not yet been described to interact with SH3 domains. On the other hand, CAPZ regulators are known to interact with the stalk region of CAPZ via the capping protein interacting motif, CPI, that is annotated by the ELM database (Kumar *et al.*, 2022) (http://elm.eu.org/elms/LIG_ActinCP_CPI_1). The regular expression describing the respective motif is rather long and complex, it has been built based on the alignments of known CAPZ-interacting partners (see alignment in [McConnell *et al.*, 2020] Figure 1) and contains both universally conserved and protein family–specific residues in a position-specific manner. Although Tks4 does not contain the annotated motif pattern, when only the universally conserved residues were retained and the family-specific positions were omitted from the regular expression, the resulting motif pattern (L……R[PAVT][KRH][^DEN].{1,5}((R.)|(.[RK]))..[PAS] where. stands for any amino acid, amino acids in a square bracket can all be accepted in a given position, amino acids preceded by ^ in a square bracket are not accepted in that position and numbers in the curly bracket indicate a range of how many times the residue in the preceding position can occur) could be found in human Tks4 using the SLiMSearch4 webserver (Krystkowiak and Davey, 2017) with default settings that searches, scores and ranks a given motif pattern (provided as regular expression) in the proteins of a selected proteome. Tks4 scored equally high as known CAPZ interactors; the CPI-like motif starting at residue L636 of Tks4 was ranked 11th in the human proteome, making it a very promising candidate to bind CAPZ.

### PLA

To confirm the interaction between CAPZA1 and Tks4 in different lung cancer cell lines (A549, HOP-92, NCI-H460), the Duolink Proximity Ligation Assay (Merck, Sigma-Aldrich, catalog no.: DUO92008, DUO92004, DUO92002, DUO82049) was performed. The assay was carried out according to the manufacturer’s instructions. First, cells were seeded into a gelatin-coated 12-well removable ibidi chamber with 5000 cells per well. The cells were fixed with 4% PFA for 15 min at RT. After a washing step with DPBS, the cells were permeabilized using 0.1% Triton X-100 in sterile DPBS for 10 min at RT. Following permeabilization, the cells were blocked with a blocking solution for 1 h at RT. Primary antibodies against mouse CAPZA1 (Santa Cruz Biotechnology: sc-374302) and rabbit Tks4 (Lányi *et al.*, 2011) were added to the wells, and the cells were incubated overnight at 4°C. Negative control wells without primary antibodies were also included. The next day, PLA probes were added to the wells and incubated for 1 h at 37°C. Subsequently, a ligation step was performed at 37°C for 30 min. This was followed by an amplification step conducted at 37°C for 100 min. After the final washes, the slide was counterstained with DAPI (Thermo Fisher Scientific, catalog no.: 62248) and mounted using FluorSave medium (Merck, Millipore, catalog no.: 345789). Images were captured using a Zeiss LSM-710 confocal microscope with a 63x objective. Randomly selected 10 images were captured for analysis from each sample type. The Duolink PLA allowed visualization of the red dots indicating the interaction between CAPZA1 and Tks4 at a proximity level (40 nm), providing evidence for their physical association.

### Availability of data and materials

In this study, open-access datasets were evaluated. The reference (WT) Tks4 sequence were downloaded from NCBI GenBank: https://www.ncbi.nlm.nih.gov/genbank/ (Homo sapiens SH3 and PX domains 2B [SH3PXD2B], transcript variant 1, mRNA: NCBI Reference Sequence: NM_001017995.3).The data of the protein–protein interaction analysis of Tks4 partner molecules were derived from the String database (https://string-db.org/). The dataset of Tks4-IP-MS analysis can be found in Supplemental Table S4. Initial version of Supplemental Table S4 raw dataset has been deposited in preprint repository bioRxiv, accession number:10.1101/2023.01.13.523903) (Kurilla *et al.*, 2023). Tks4 (SH3PXD2B) mRNA expression levels in patients with LUAD and LUSC obtained from the GEPIA2 database (http://gepia2.cancer-pku.cn/#index). Prognostic analysis of Tks4 gene expression on overall survival of patients with lung cancer was investigated using GEPIA2 database (http://gepia2.cancer-pku.cn/#index).

The crystal structure of the CD2AP with the CAPZ heterodimer (PDB ID: 3AA6, https://www.rcsb.org/structure/3AA6) and the CKIP with the CAPZ heterodimer (PDB ID: 3AA1, https://www.rcsb.org/structure/3AA1), were obtained from the Protein Data Bank (PDB) RCSB.org database (https://www.rcsb.org/). Identification of potential binding sites between Tks4 and CAPZ were analysed using the SLiMSearch 4 (http://slim.icr.ac.uk/slimsearch/index.php). The TissueScan Lung Cancer cDNA Array III datasets can be found in Supplemental Table S3.

### Statistical analyses

Statistical analysis was performed using GraphPad Prism 10.2.3 software. Student’s unpaired *t* test or ANOVA with Tukey’s post hoc test was used to determine statistical significance.

To test the power of the Tks4 expression level as a biomarker, ROC curve analyses were performed based on our lung cancer tissue cDNA array data and the clinical sensitivity and specificity of TKS4 expression were compare in distinguishing normal and tumorous tissue. ROC curve analyses were performed using GraphPad Prism 8.0.1. Additionally, the colocalization coefficient of cortactin and F-actin proteins was calculated using the EzColocalization plugin in Image J. The statistical significance was defined at *p* < 0.05 or *p* < 0.001 and the *p*-values were indicated on the figures with asterisk.

## Supplementary Material



## Abbreviations used:

ECM: extracellular matrix
EMT: epithelial-to-mesenchymal transition
ICC: immunocytochemistry
IP: immunoprecipitation
LUAD: lung adenocarcinoma
LUSC: lung squamous cell carcinoma
MS: *mass spectrometry*
PLA: proximity ligation assay
ROC: receiver operating characteristic curve
TCGA: The Cancer Genome Atlas
WB: Western blot
