# Supplementary material for: Unveiling epithelial plasticity regulation in lung cancer: Exploring the cross-talk among Tks4 scaffold protein partners
Source: Mol Biol Cell. 2024 Jul 22;35(8):ar111. doi: 10.1091/mbc.E24-03-0103 (PMC11321040; doi:10.1091/mbc.E24-03-0103)
Supplement: Supplementary file 1 [file mbc-35-ar111-s001.pdf]

Supplemental Materials

Molecular Biology of the Cell

László *et al*.

Supplementary Figure 1 A

Karyotyping of A549 WT cells by UDGenomed, Debrecen, Hungary

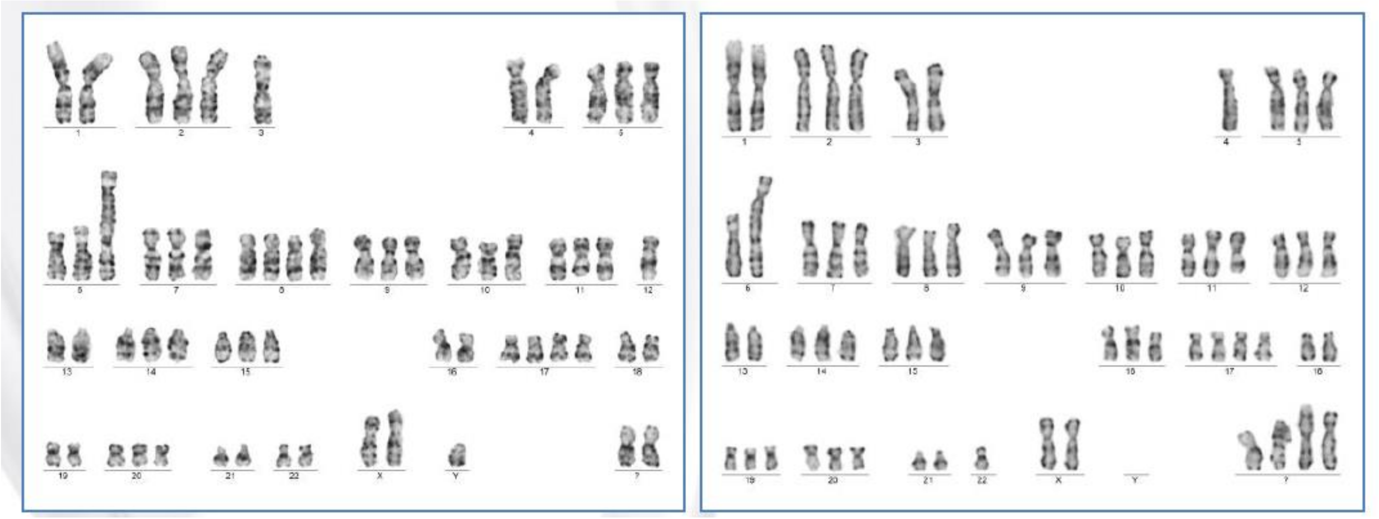

Mycoplasma detection by Microsynth AG

4.1. Summary Table

Your sample-ID	Microsynth-ID	check for PCR inhibition	contaminated
A549 WT	011352	no inhibition	no
A549 Clone 1	011353	no inhibition	no
A549 Clone 5	011354	no inhibition	no

Supplemetary Figure 1 B
Cell line authentification by Microsynth AG

A549 WT: The analyzed data of the submitted sample match 100 % to the DNA profile of the cell line A-549 (Cellosaurus, RRID:CVCL_0023).

4.2. Electropherogram

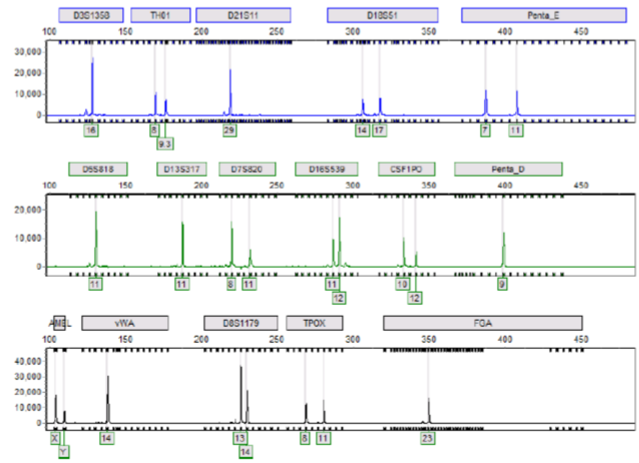

4. Analysis Results

4.1. Summary Table of the STR Profile

Locus	Chromosomal Location	Core STR Marker	Customer Sample Typed Alleles	Database Alleles	Comments
D3S1358	Chr03		16	16	
TH01	Chr11	Yes	8/9.3	8/9.3	
D21S11	Chr21		29	29	
D18S51	Chr18		14/17	14/17	
Penta_E	Chr15		7/11	7/11	
D5S818	Chr05	Yes	11	11	
D13S317	Chr13	Yes	11	11	
D7S820	Chr07	Yes	8/11	8/11	
D16S539	Chr16	Yes	11/12	11/12	
CSF1PO	Chr05	Yes	10/12	10/12	
Penta_D	Chr21		9	9	
AMEL	X/Y	Yes	X/Y	X/Y	
vWA	Chr12	Yes	14	14	
D8S1179	Chr08		13/14	13/14	
TPOX	Chr2	Yes	8/11	8/11	
FGA	Chr04		23	23	

A549 Tks4-KO 1: The analyzed data of the submitted sample match 100 % to the DNA profile of the cell line A-549 (Cellosaurus, RRID:CVCL_0023).

4.2. Electropherogram

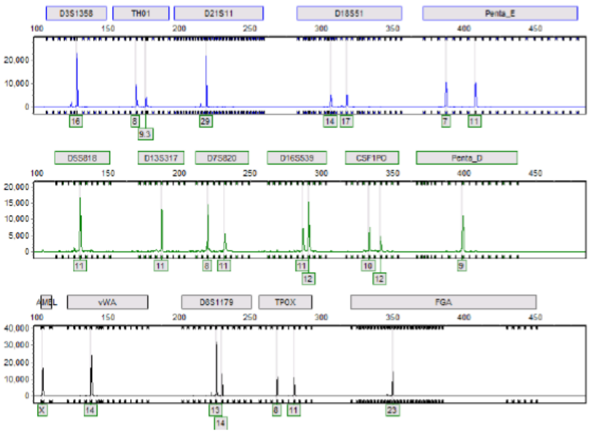

4. Analysis Results

4.1. Summary Table of the STR Profile

Locus	Chromosomal Location	Core STR Marker	Customer Sample Typed Alleles	Database Alleles	Comments
D3S1358	Chr03		16	16	
TH01	Chr11	Yes	8/9.3	8/9.3	
D21S11	Chr21		29	29	
D18S51	Chr18		14/17	14/17	
Penta_E	Chr15		7/11	7/11	
D5S818	Chr05	Yes	11	11	
D13S317	Chr13	Yes	11	11	
D7S820	Chr07	Yes	8/11	8/11	
D16S539	Chr16	Yes	11/12	11/12	
CSF1PO	Chr05	Yes	10/12	10/12	
Penta_D	Chr21		9	9	
AMEL	X/Y	Yes	X	X/Y	
vWA	Chr12	Yes	14	14	
D8S1179	Chr08		13/14	13/14	
TPOX	Chr2	Yes	8/11	8/11	
FGA	Chr04		23	23	

A549 Tks4-KO 2: The analyzed data of the submitted sample match 100 % to the DNA profile of the cell line A-549 (Cellosaurus, RRID:CVCL_0023).

4.2. Electropherogram

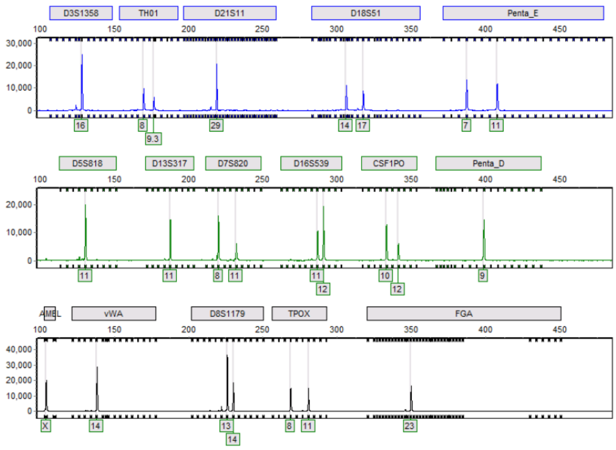

4. Analysis Results

4.1. Summary Table of the STR Profile

Locus	Chromosomal Location	Core STR Marker	Customer Sample Typed Alleles	Database Alleles	Comments
D3S1358	Chr03		16	16	
TH01	Chr11	Yes	8/9.3	8/9.3	
D21S11	Chr21		29	29	
D18S51	Chr18		14/17	14/17	
Penta_E	Chr15		7/11	7/11	
D5S818	Chr05	Yes	11	11	
D13S317	Chr13	Yes	11	11	
D7S820	Chr07	Yes	8/11	8/11	
D16S539	Chr16	Yes	11/12	11/12	
CSF1PO	Chr05	Yes	10/12	10/12	
Penta_D	Chr21		9	9	
AMEL	X/Y	Yes	X	X/Y	
vWA	Chr12	Yes	14	14	
D8S1179	Chr08		13/14	13/14	
TPOX	Chr2	Yes	8/11	8/11	
FGA	Chr04		23	23	

Supplementary Figure 2 A

Full lenght blots for Figure 1 B

Stain-free gel

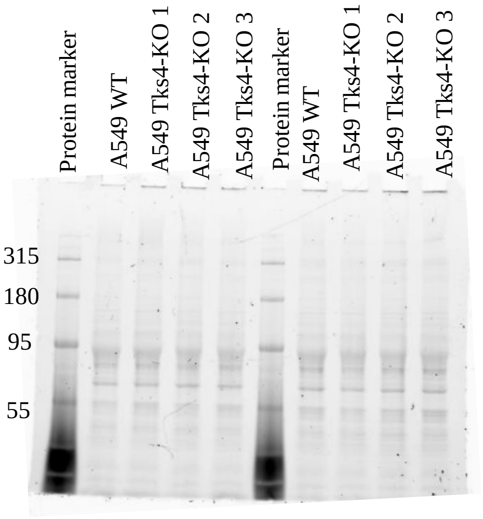

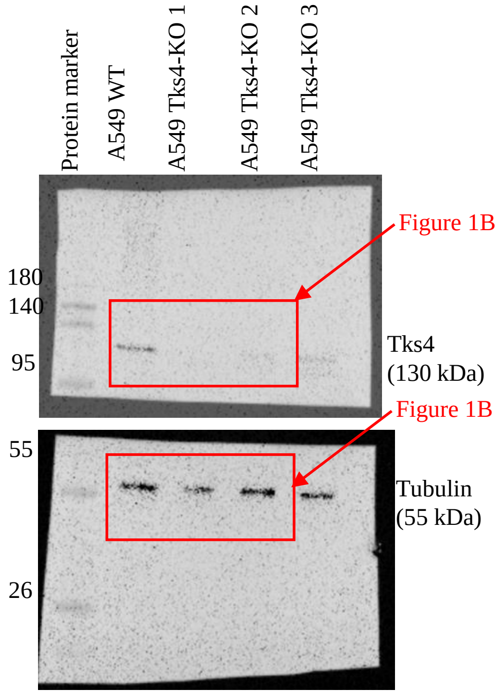

Full lenght blots for Figure 2 A

Stain-free gel

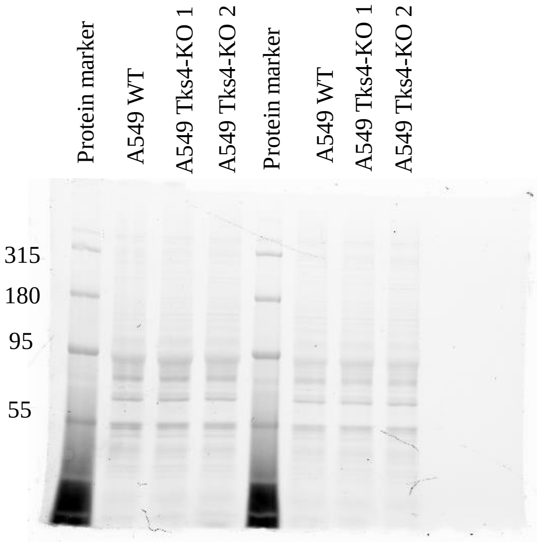

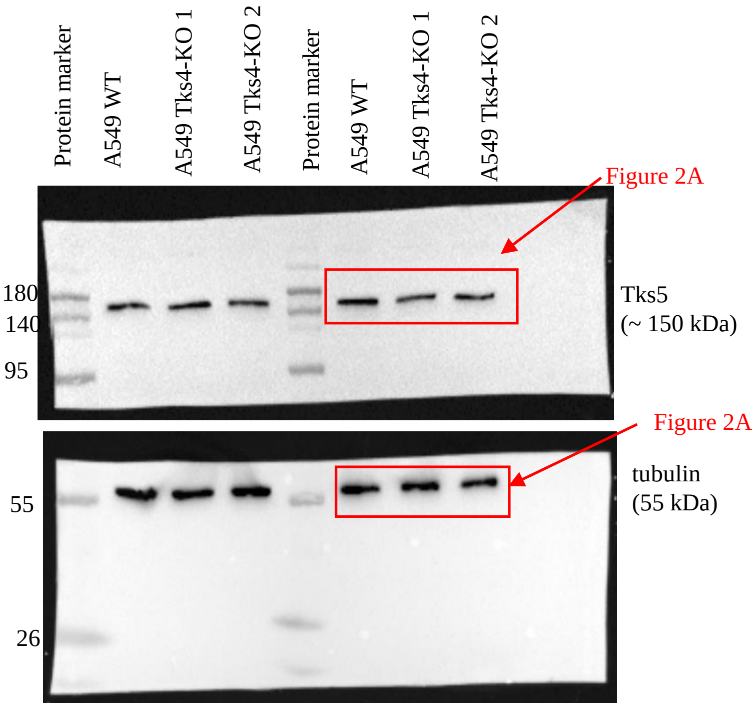

Full lenght blots for Figure 2 B

Stain-free gel

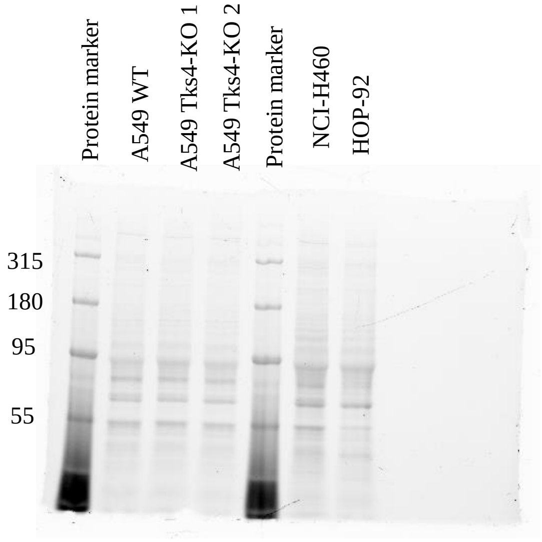

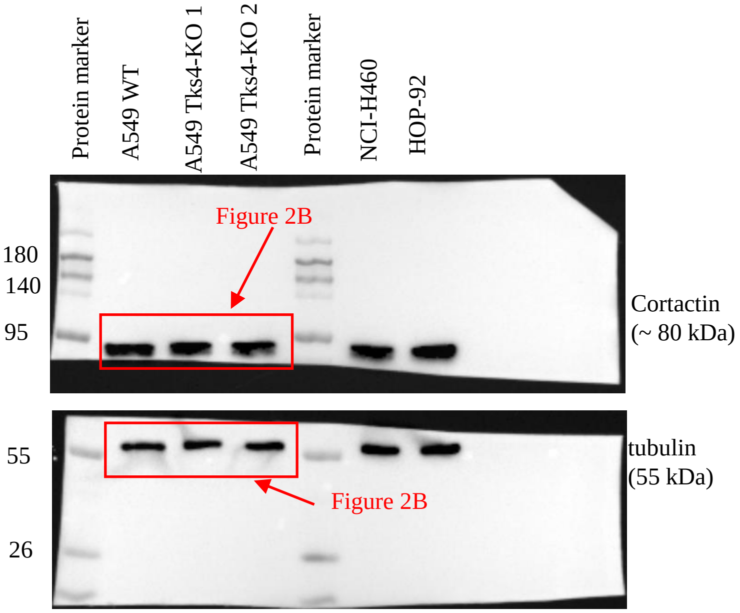

Supplementary Figure 2 B

Full lenght blots for Figure 4 B-C-D and 8B

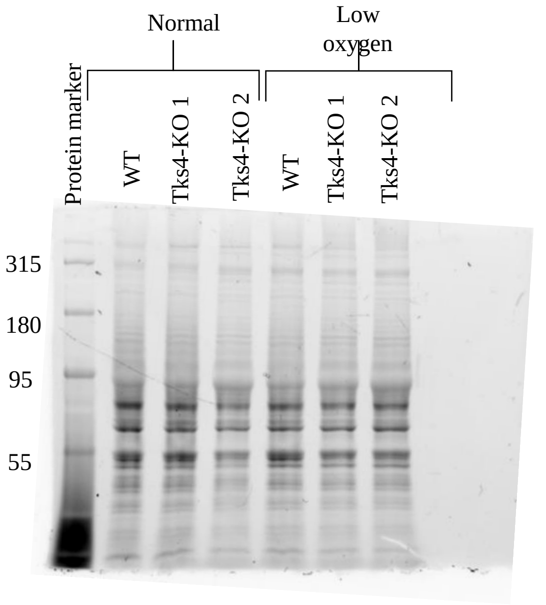

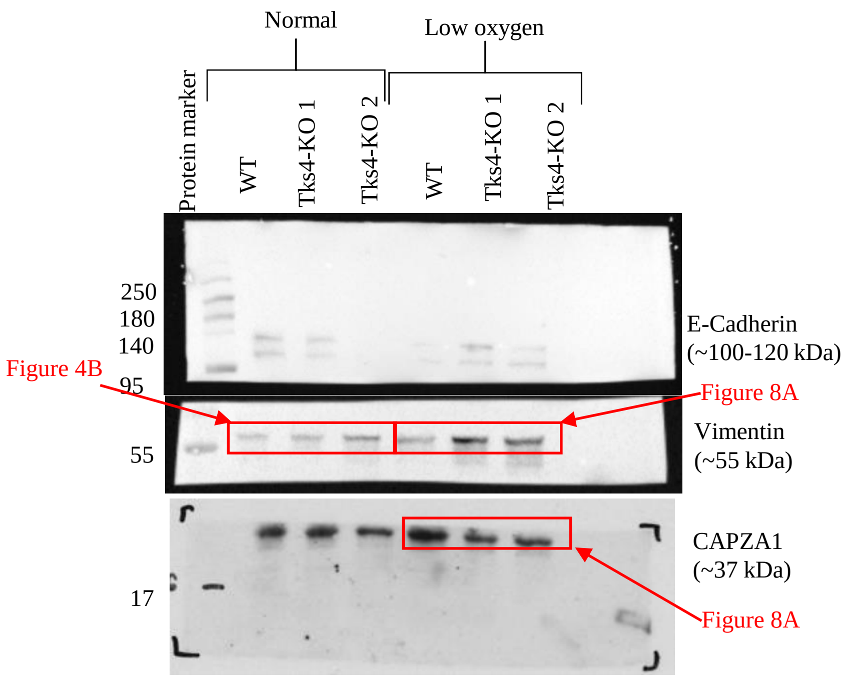

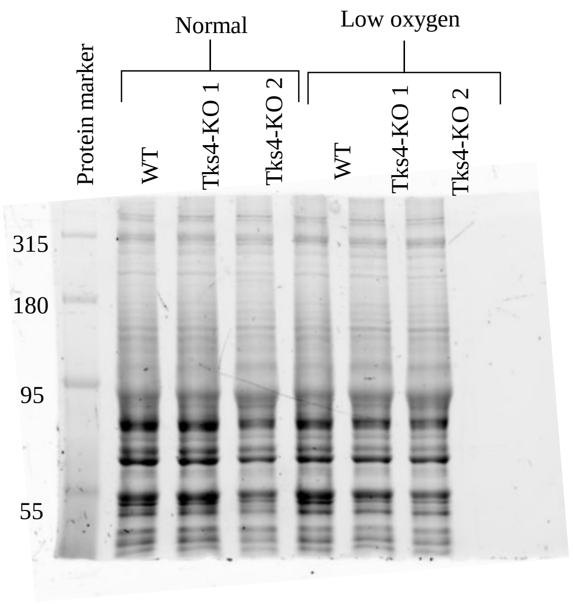

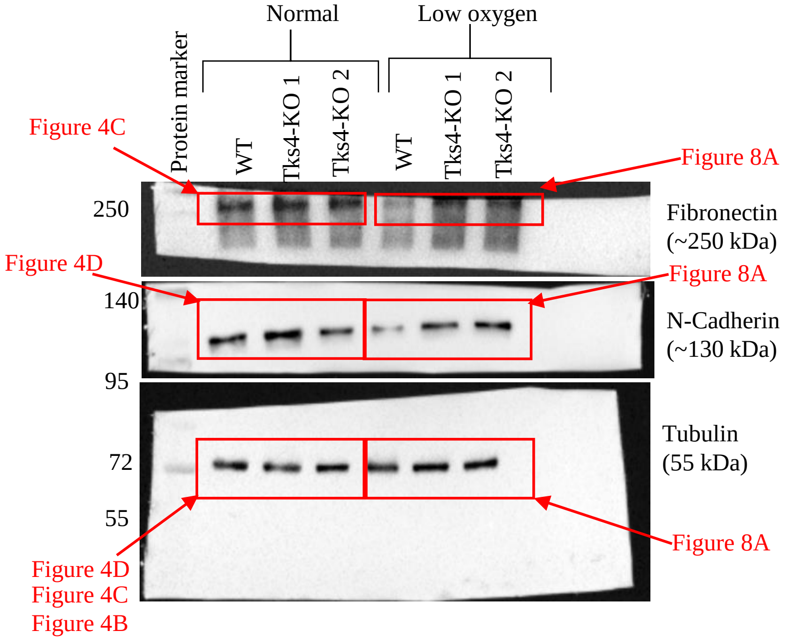

Supplementary Figure 2 C

Full lenght blots for Figure 6 B: Tks4-IP in A549 WT – Grb2, Cortactin, CD2AP, CAPZA1 WB

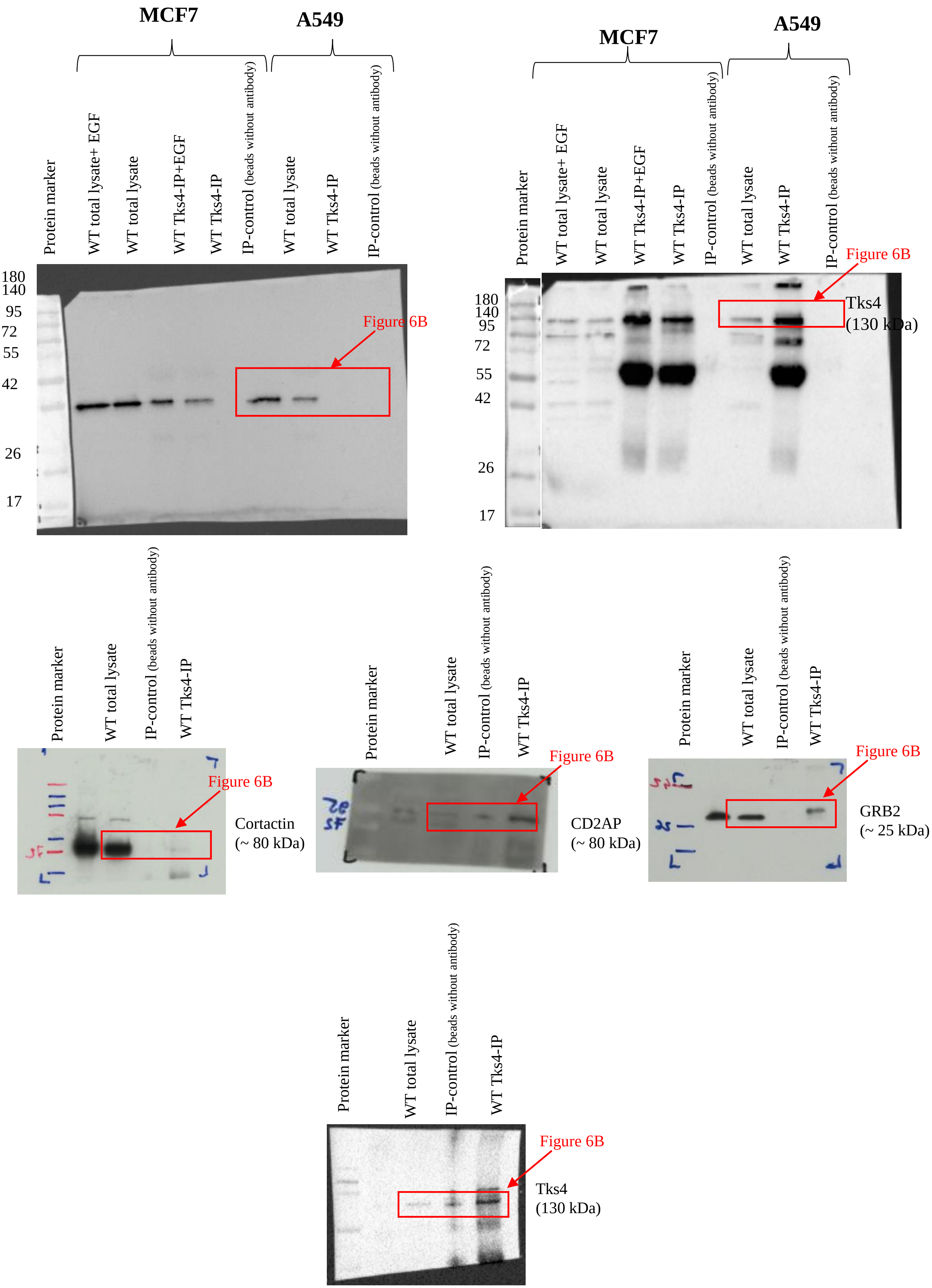

Supplementary Figure 2 D

Full lenght blots for Figure 6 B : Tks4-IP in NCI-H460 – Grb2, Cortactin, CD2AP, CAPZA1 WB

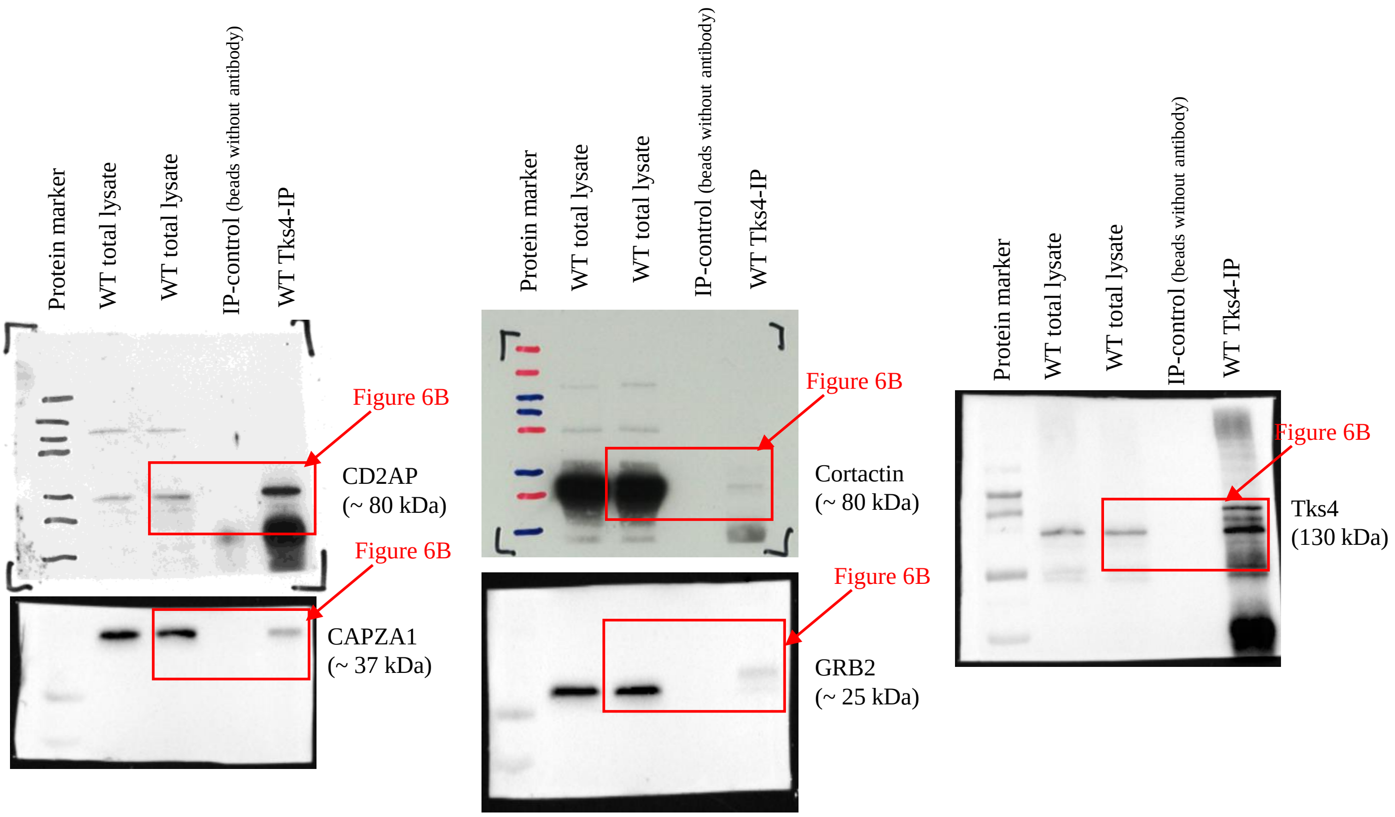

Full lenght blots for Figure 6 B : Tks4-IP in HOP-92 – Grb2, Cortactin, CD2AP, CAPZA1 WB

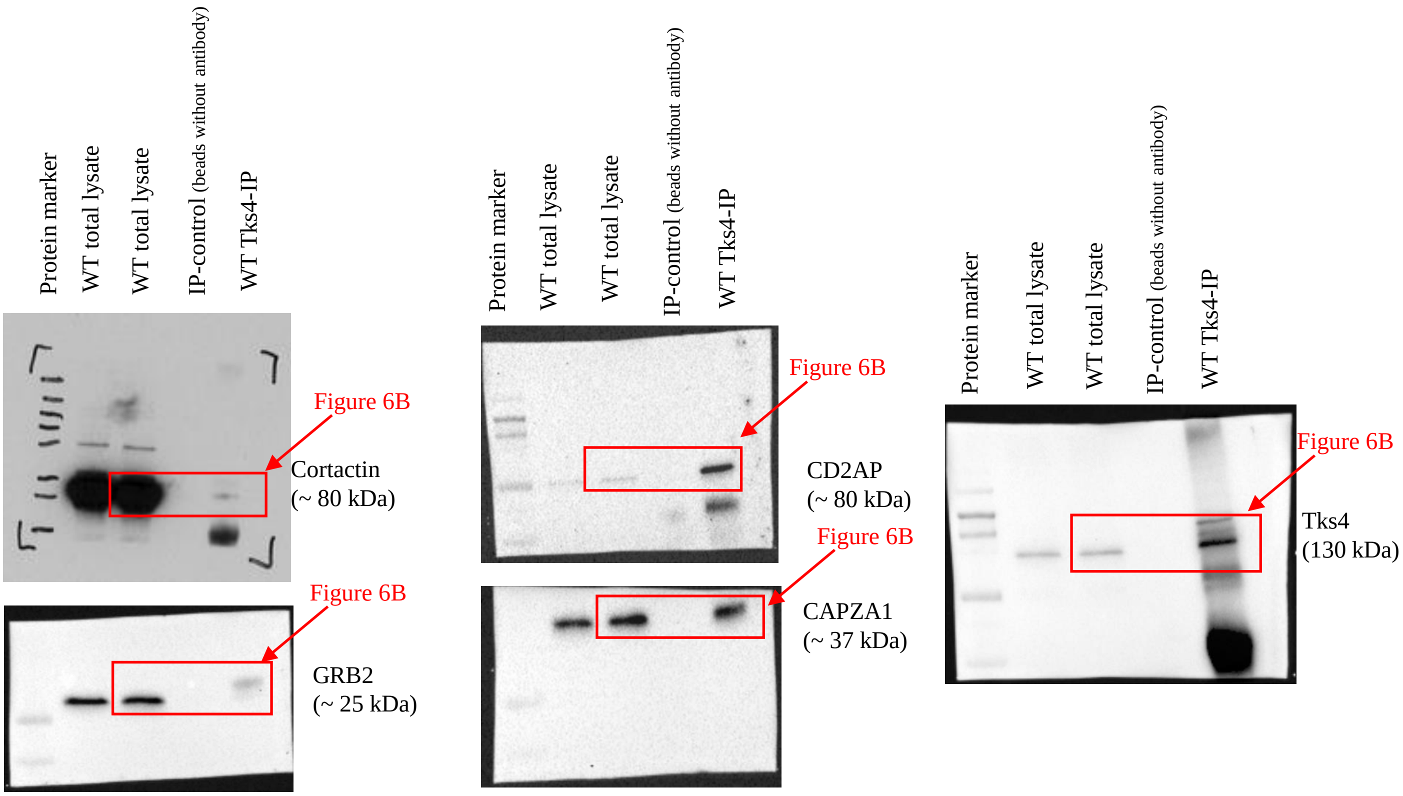

Supplementary Figure 2 E

Full lenght blots and repeated experiments for Figure 1 B

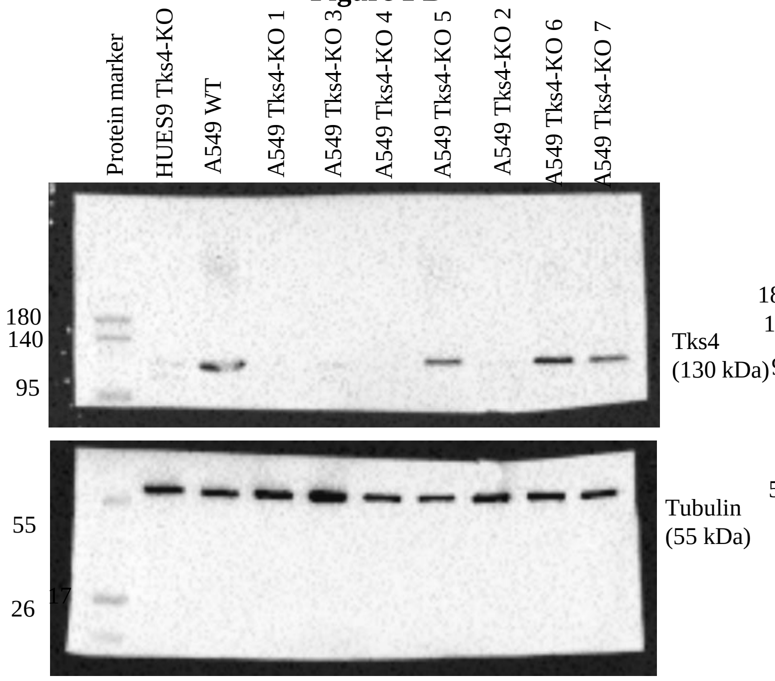

Full lenght blots and repeated experiments for Figure 2 A

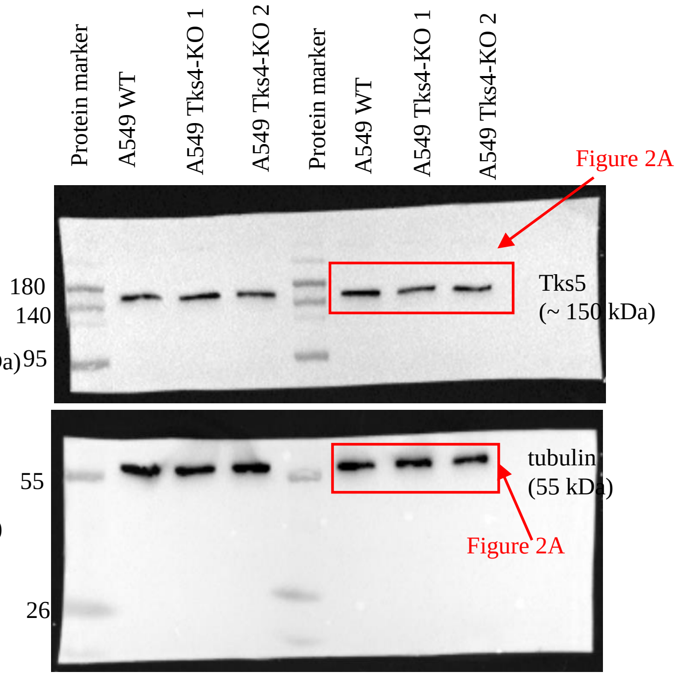

Full lenght blots and repeated experiments for Figure 2 B : A549 WT, Tks4-KO1, Tks4-KO2 – Cortactin WB

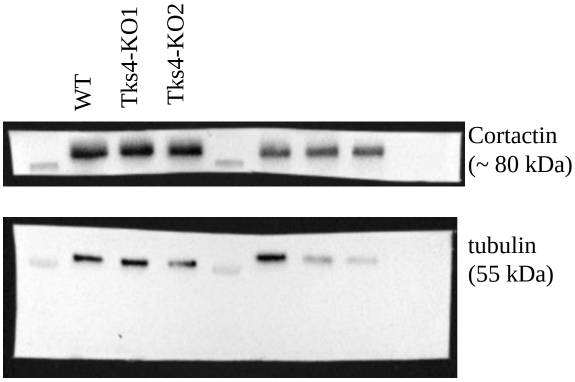

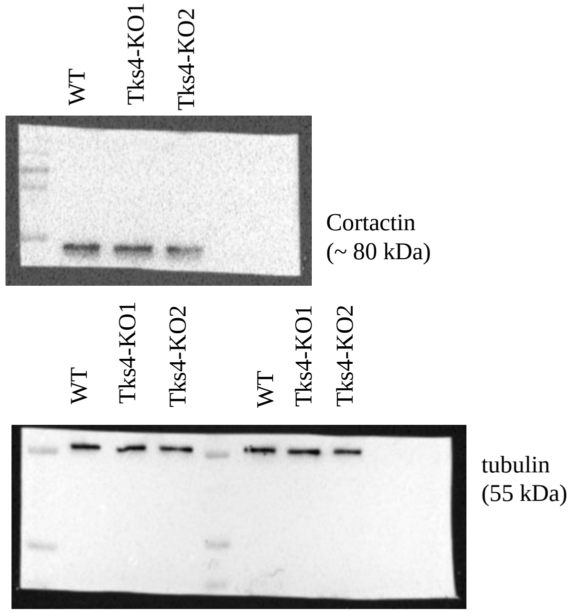

Supplementary Figure 2 F

Full lenght blots and repeated experiments for 4 B-C-D and 8B

Stain-free gel

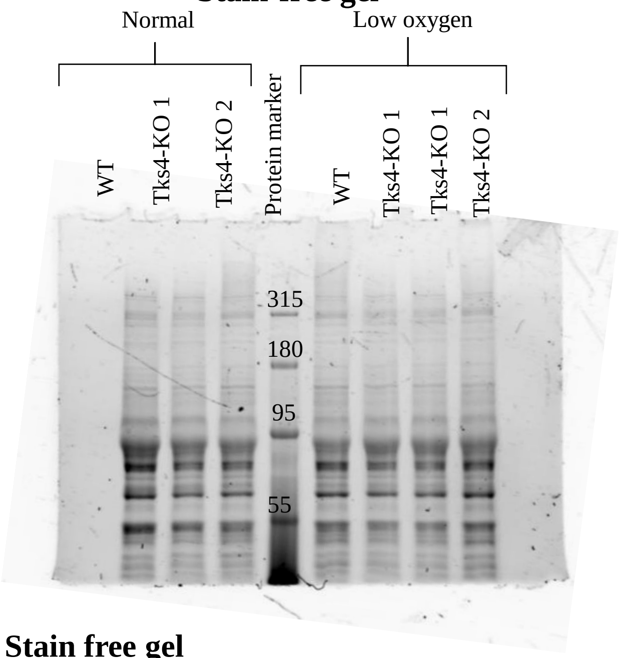

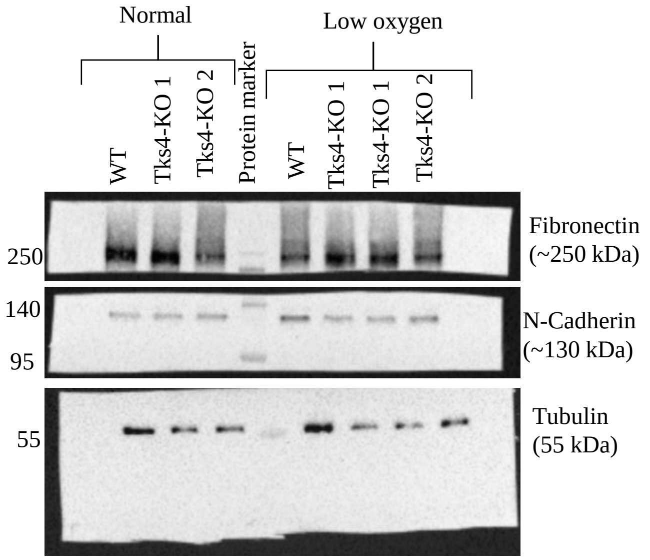

Stain free gel

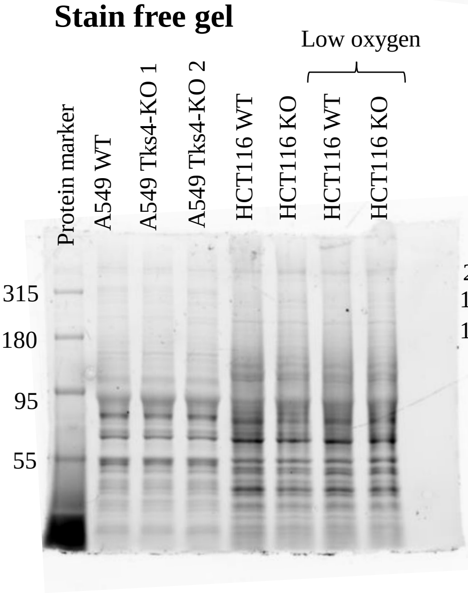

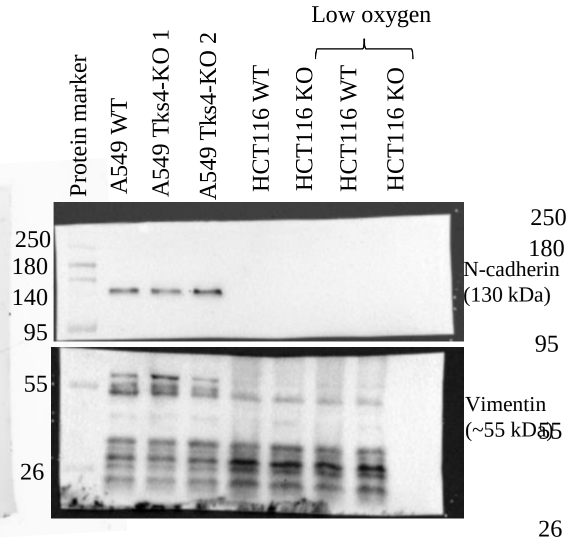

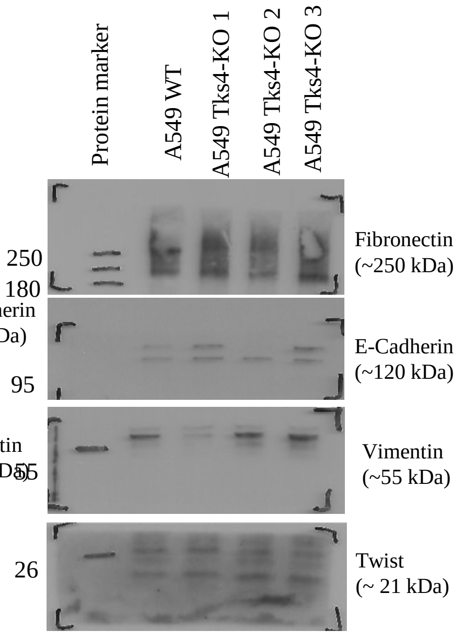

Stain free gel

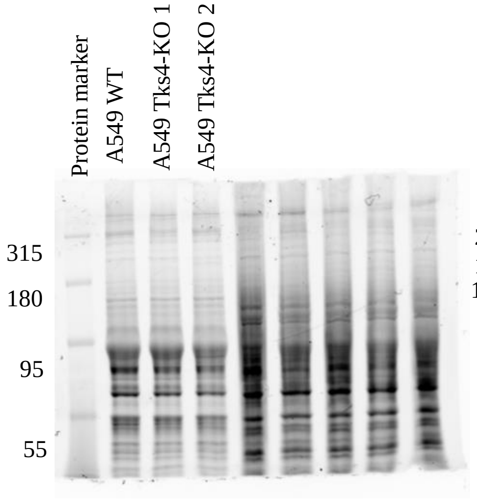

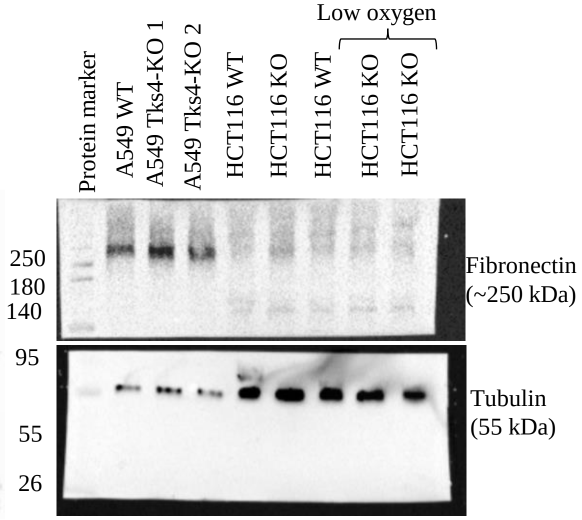

Supplementary Figure 2 G

Full lenght blots and repeated experiments for Figure 3 B-C-D

Stain free gel

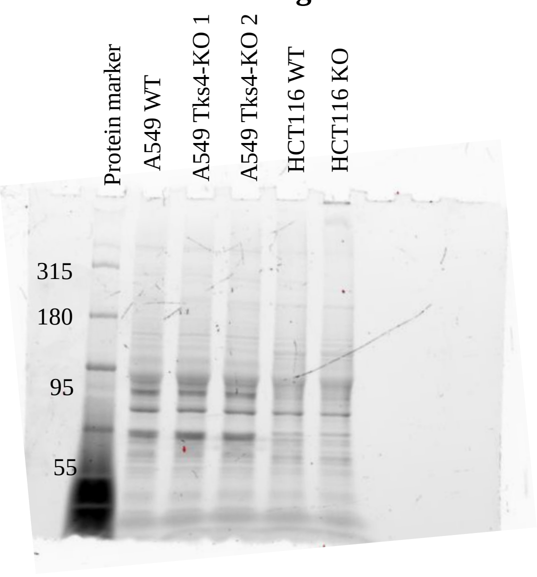

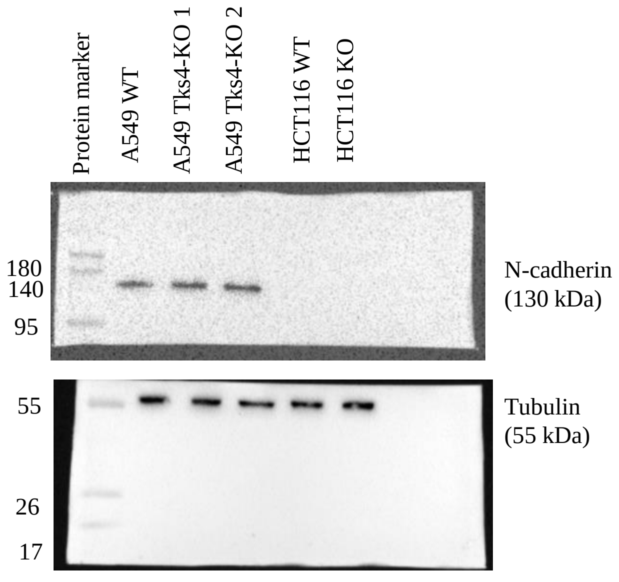

Stain free gel

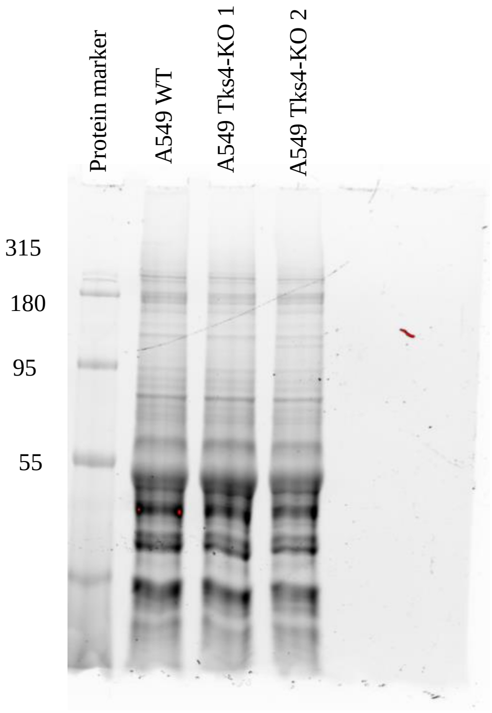

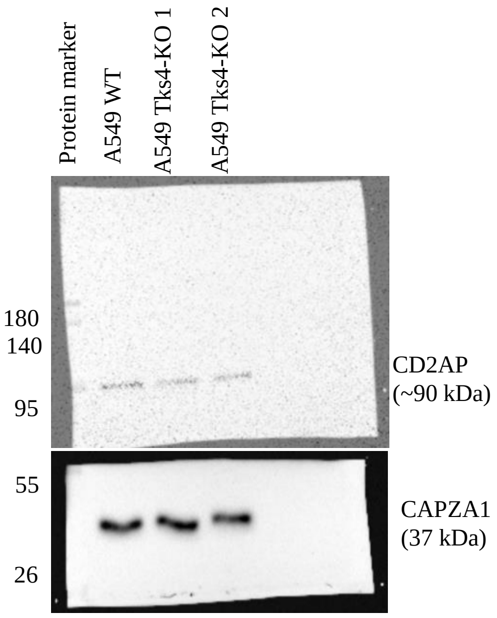

Supplementary Figure 2 H

Full lenght blots and repeated experiments for Figure 4-B-C-D and 8B

Stain free gel

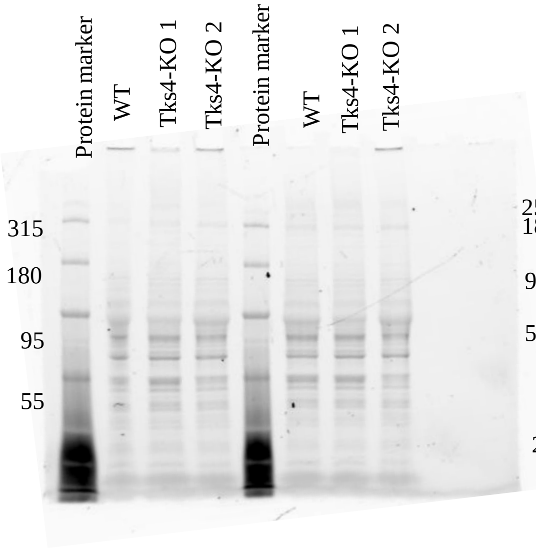

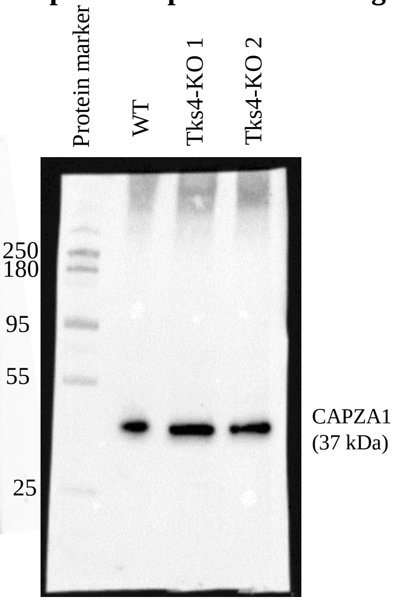

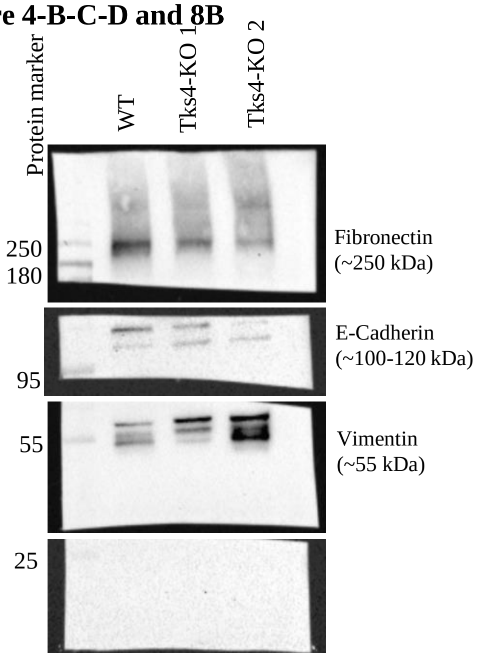

Stain free gel

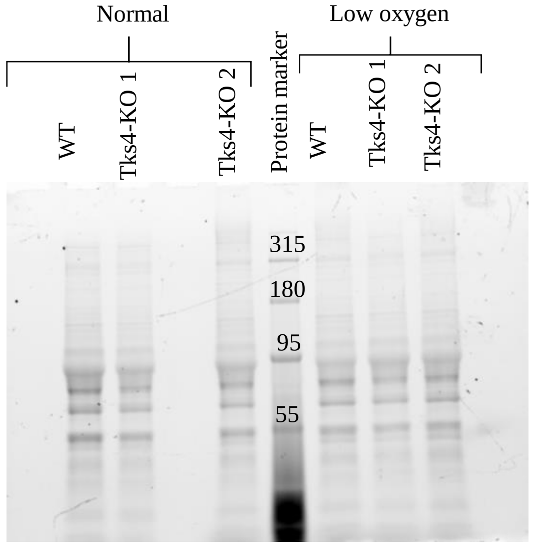

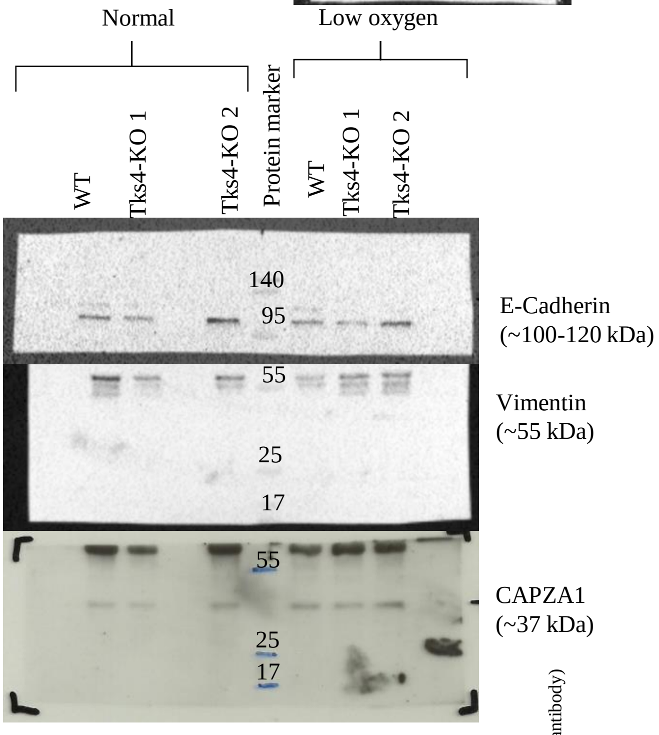

Full lenght blots and repeated experiments for Figure 6 B- A549 Tks4 IP-CAPZA1 WB

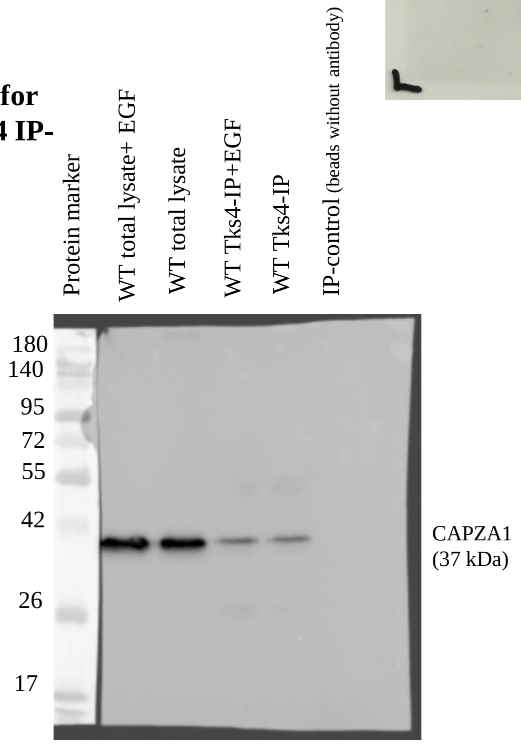

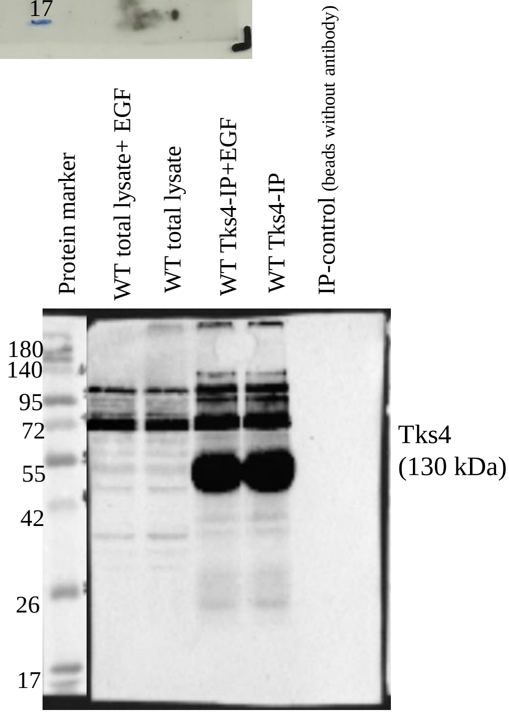

Supplemetary Figure 3

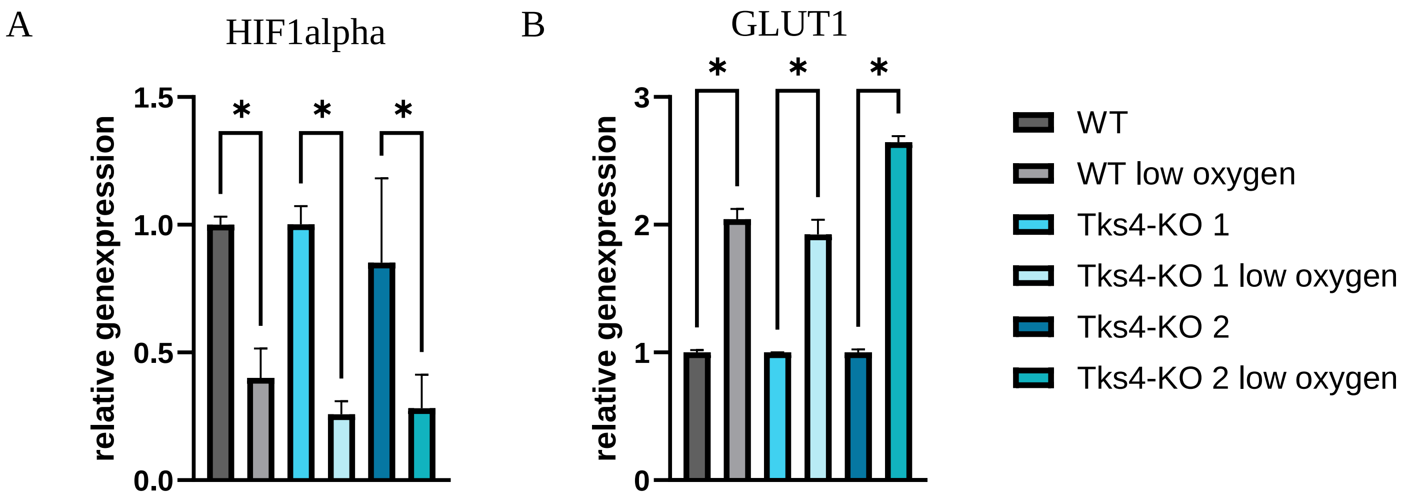

Supplemetary table 1: List of all used RT-qPCR reagents

RT-qPCR reagents (Taqman probes, primers, etc.)	catalog number
FIRST STRAND CDNA SYNT. KIT FOR RT-PCR	Meck, Roche: 11483188001
Direct-zol™ RNA Miniprep Kit	Zymo Research: R2052
TRIzol Reagent	ambion, Life Technologies: 15596026
GAPDH Taqman probe	Thermofisher Scientific:<br>Assay ID: Hs04420632_g1
E-Cadherin forward primer	sequence: TGCCCAGAAAATGAAAAAGG
E-Cadherin reverse primer	sequence:<br>GTGTATGTGGCAATGCGTTC
TaqMan™ Fast Advanced Master Mix	Thermofisher Scientific: 4444557
Power SYBR™ Green PCR Master Mix	Thermofisher Scientific: 4368577
TWIST1 Taqman probe	Thermofisher Scientific, Assay ID:<br>Hs04989912_s1
FN1 Taqman probe	Thermofisher Scientific, Assay ID:<br>Hs01549976_m1
SNAI1 Taqman probe	Thermofisher Scientific, Assay ID:<br>Hs00195591_m1
SNAI2 Taqman probe	Thermofisher Scientific, Assay ID:<br>Hs00161904_m1
GLUT1/ SLC2A1 Taqman probe	Thermofisher Scientific, Assay ID:<br>Hs00892681_m1
Vimentin forward primer	sequence: TACAGGAAGCTGCTGGAAGG
Vimentin reverse primer	sequence: ACCAGAGGGAGTGAATCCAG
N-cadherin forward primer	sequence: TGACAATGACCCCACAGCTC
N-cadherin reverse primer	sequence: GTCCTGCTCACCACCACTAC
Zeb1 forward primer	sequence: GGGAGGAGCAGTGAAAGAGA
Zeb1 reverse primer	sequence: TTTCTTGCCCTTCCTTTCTG
GAPDH forward primer	sequence: TGCACCACCAACTGCTTAGC
GAPDH reverse primer	sequence: GGCATGGACTGTGGTCATGAG

Supplemetary Table 2: List of all applied antibodies, dyes with dilutions

Antibodies, dyes	catalog number	dilution
Vimentin (rabbit polyclonal)	Abcam: ab137321	WB: 1000x,<br>ICC: 100x
E-Cadherin (rabbit polyclonal)	Abcam: ab15148	WB: 500x
Fibronectin (rabbit polyclonal)	Abcam: ab45688	WB: 2000x,<br>ICC:100x
N-Cadherin (3B9, mouse-monoclonal)	Thermofisher Scientific: 333900	WB: 500x,<br>ICC: 100x
alpha-Tubulin (mouse monoclonal)	Merck, Sigma-Aldrich: T6199	WB: 1000x
Phalloidin, CF 543	Biotium: 00043	ICC: 40x
CAPZA1 (H-9, mouse monoclonal)	Santa Cruz: sc-374302	WB: 500x<br>ICC: 50x<br>PLA: 50x
CAPZA1 (A2E7,mouse monoclonal)	Invitrogen: MA5-36109	WB: 500x
Tks4 (rabbit polyclonal)	(50)*	WB: 1000x,<br>ICC: 1000 x,<br>PLA: 100x<br>IP: 10 ul/sample
Cortactin (H-191, rabbit polyclonal)	Santa Cruz: sc-11408	ICC: 100x

Cortactin (H-5, mouse monoclonal)	Santa Cruz: sc-55579	WB: 1000x
Tks5 (SH3PXD2A, rabbit polyclonal)	proteintech: 18976-1-AP	WB: 500x<br>ICC: 25x
CD2AP (5F8, mouse monoclonal)	Invitrogen: MA5-33009	WB: 1000x
GRB2 (3F2, mouse monoclonal)	Sigma-Aldrich: 05-372	WB: 1000x
Anti-Mouse IgG (whole molecule)–Peroxidase antibody produced in sheep	Merck, Sigma-Aldrich: A6782	WB: 1000x
RABBIT IGG HRP-LINKED WHOLE AB (DONKEY)	avantor by vwr: NA934	WB: 1000x
DAPI	Thermofisher Scientific: 62248	ICC: 1000x
PROTEIN A SEPHAROSE 4 FAST FLOW	Merck, Sigma-Aldrich: P9424	IP: 50 ul beads/sample
Goat-Anti-Mouse antibody, Alexa Fluor 488	Thermo Fisher Scientific: A-11029	ICC: 1000x
Goat anti-Rabbit antibody, Alexa Fluor 488	Thermo Fisher Scientific A-11008	ICC: 1000x
Goat-Anti-Rabbit antibody, Alexa Fluor 546	Thermo Fisher Scientific: A11035	ICC: 1000x
HIF-1-ALPHA (EPR16897, rabbit monoclonal)	Abcam: ab179483	WB: 500x

*Lányi, Á. et al. (2011) 'The Homolog of the Five SH3-Domain Protein (HOFI/SH3PXD2B) Regulates Lamellipodia Formation and Cell Spreading', PLOS ONE, 6(8), p. e23653. doi: 10.1371/JOURNAL.PONE.0023653.

Supplemetary table 3: TissueScan Lung Cancer cDNA Array III (Origene, Cat.: HLRT103) data

well position in array	gender	age	appearance	sample diagnosis from pathology verification	minimum STAGE group	result: $2^{-\Delta\Delta Ct}$
C01	Female	69	Normal	Within normal limits	Not Applicable	1,202584
C02	Male	63	Normal	Within normal limits	Not Applicable	0,773505
C03	Male	72	Normal	Within normal limits	Not Applicable	0,992193
C04	Female	64	Normal	Within normal limits	Not Applicable	1,037881
C05	Female	66	Normal	Within normal limits	Not Applicable	0,904898
C06	Male	45	Normal	Within normal limits	Not Applicable	0,817249
C07	Male	78	Normal	Within normal limits	Not Applicable	1,375502
C08	Female	72	Normal	Within normal limits	Not Applicable	1,026272
C09	Male	69	Tumor	Carcinoma of lung, squamous cell	IA	0,456448
C10	Female	41	Tumor	Carcinoma of lung, large cell	IA	0,250672
C11	Female	61	Tumor	Adenocarcinoma of lung	IA	0,28112
C12	Female	68	Tumor	Carcinoma of lung, bronchioloalveolar, non-mucinous	IA	0,842831
D01	Female	34	Tumor	Adenocarcinoma of lung	IA	1,262933
D02	Male	67	Tumor	Adenocarcinoma of lung	IA	0,450399
D03	Female	71	Tumor	Carcinoma of lung, squamous cell	IB	0,188854

D04	Male	74	Tumor	Carcinoma of lung, squamous cell	IB	0,115397
D05	Male	77	Tumor	Carcinoma of lung, squamous cell	IB	0,21377
D06	Female	74	Tumor	Adenocarcinoma of lung, bronchioloalveolar	IB	0,682937
D07	Female	49	Tumor	Carcinoma of lung, neuroendocrine	IB	0,286001
D08	Male	80	Tumor	Carcinoma of lung, squamous cell	IB	0,421481
D09	Not Specified	71	Tumor	Adenocarcinoma of lung	IIA	0,164536
D10	Male	63	Tumor	Carcinoma of lung, small cell, metastatic	IIA	0,227609
D11	Male	69	Tumor	Carcinoma of lung, squamous cell	IIA	0,441119
D12	Male	72	Tumor	Adenocarcinoma of lung	IIB	0,640254
E01	Male	61	Tumor	Carcinoma of lung, squamous cell	IIB	0,168581
E02	Male	55	Tumor	Adenocarcinoma of lung	IIB	0,297345
E03	Male	55	Tumor	Carcinoma of lung, squamous cell	IIB	0,659688
E04	Female	79	Tumor	Adenocarcinoma of lung	IIB	0,409387
E05	Female	66	Tumor	Adenocarcinoma of lung	IIB	0,197045
E06	Male	65	Tumor	Carcinoma of lung, squamous cell	IIB	0,366078
E07	Male	90	Tumor	Carcinoma of lung, squamous cell	IIB	0,092435
E08	Male	74	Tumor	Carcinoma of lung, squamous cell	IIIA	0,300184
E09	Female	71	Tumor	Carcinoma of lung, adenosquamous	IIIA	0,416219
E10	Male	69	Tumor	Carcinoma of lung, large cell, neuroendocrine	IIIA	0,391308
E11	Male	68	Tumor	Carcinoma of lung, small cell	IIIA	0,273858
E12	Male	75	Tumor	Adenocarcinoma of lung	IIIA	0,290345
F01	Male	81	Tumor	Carcinoma of lung, sarcomatoid	IIIB	0,506136
F02	Female	64	Tumor	Adenocarcinoma of lung, bronchioloalveolar	IIIB	0,632765

F03	Female	56	Tumor	Carcinoma of lung, adenosquamous	IIIB	0,318392
F04	Male	44	Tumor	Adenocarcinoma of lung, bronchioloalveolar	IIIB	0,598067
F05	Male	51	Tumor	Carcinoma of lung, squamous cell	IIIB	0,153541
F06	Female	46	Tumor	Adenocarcinoma of lung	IV	0,563833
F07	Male	61	Tumor	Carcinoma of lung, non-small cell, metastatic	IV	0,064946
F08	Female	64	Tumor	Carcinoma of lung, non-small cell, metastatic	IV	0,145303
F09	Female	63	Tumor	Carcinoma of lung, squamous cell, metastatic	IV	0,096689
F10	Male	72	Tumor	Adenocarcinoma of lung	IV	0,130062
F11	Male	77	Tumor	Adenocarcinoma of lung, metastatic	IV	0,202263
F12	Male	65	Tumor	Adenocarcinoma of lung, metastatic	IV	0,32084

Supplementary table 4. Tks4 immunoprecipitation (IP)–mass spectrometry (MS) analysis in five cell lines (A549, HCT116, MCF7, HPAC, N87). (Control samples are treated the same way as the anti-Tks4-IP sample but without the usage of anti-Tks4 antibody.) (Initial version of raw dataset is deposited in preprint repository bioRxiv, accession number: 10.1101/2023.01.13.523903)

cell line:					**unique peptide count**	
**A549**	Identified Proteins	Accession Number	Alternate ID	MW	Anti-Tks4-IP sample	control sample
1	Plectin OS=Homo sapiens OX=9606 GN=PLEC PE=1 SV=3	Q15149	PLEC	532 kDa	95	0
2	Albumin OS=Homo sapiens OX=9606 GN=ALB PE=1 SV=2	P02768 (+2)	ALB	69 kDa	7	0
3	SH3 and PX domain-containing protein 2B OS=Homo sapiens OX=9606 GN=SH3PXD2B PE=1 SV=3	A1X283	SH3PXD2B	102 kDa	19	0
4	Tryptophan--tRNA ligase, cytoplasmic OS=Homo sapiens OX=9606 GN=WARS1 PE=1 SV=2	P23381	WARS1	53 kDa	14	0
5	Cell division cycle and apoptosis regulator protein 1 OS=Homo sapiens OX=9606 GN=CCAR1 PE=1 SV=2	Q8IX12	CCAR1	133 kDa	17	0
6	Alpha-amylase 1B OS=Homo sapiens OX=9606 GN=AMY1B PE=1 SV=1	P0DTE7 (+2)	AMY1B	58 kDa	5	3
7	Heat shock cognate 71 kDa protein OS=Homo sapiens OX=9606 GN=HSPA8 PE=1 SV=1	P11142	HSPA8	71 kDa	16	0
8	Complement C4-A OS=Homo sapiens OX=9606 GN=C4A PE=1 SV=2	P0C0L4	C4A	193 kDa	5	0
9	CD2-associated protein OS=Homo sapiens OX=9606 GN=CD2AP PE=1 SV=1	Q9Y5K6	CD2AP	71 kDa	14	0

10	Kinesin-1 heavy chain OS=Homo sapiens OX=9606 GN=KIF5B PE=1 SV=1	P33176	KIF5B	110 kDa	6	0
11	Heat shock 70 kDa protein 1A OS=Homo sapiens OX=9606 GN=HSPA1A PE=1 SV=1	P0DMV8 (+1)	HSPA1A	70 kDa	6	0
12	F-actin-capping protein subunit alpha-1 OS=Homo sapiens OX=9606 GN=CAPZA1 PE=1 SV=3	P52907	CAPZA1	33 kDa	4	0
13	Cytoplasmic dynein 1 heavy chain 1 OS=Homo sapiens OX=9606 GN=DYNC1H1 PE=1 SV=5	Q14204	DYNC1H1	532 kDa	10	0
14	Sorcin OS=Homo sapiens OX=9606 GN=SRI PE=1 SV=1	P30626	SRI	22 kDa	7	0
15	F-actin-capping protein subunit beta OS=Homo sapiens OX=9606 GN=CAPZB PE=1 SV=4	P47756	CAPZB	31 kDa	5	0
16	E3 ubiquitin-protein ligase TRIM21 OS=Homo sapiens OX=9606 GN=TRIM21 PE=1 SV=1	P19474	TRIM21	54 kDa	7	0
17	F-actin-capping protein subunit alpha-2 OS=Homo sapiens OX=9606 GN=CAPZA2 PE=1 SV=3	P47755	CAPZA2	33 kDa	4	0
18	60S ribosomal protein L11 OS=Homo sapiens OX=9606 GN=RPL11 PE=1 SV=2	P62913	RPL11	20 kDa	4	0
19	Nucleoside diphosphate kinase, mitochondrial OS=Homo sapiens OX=9606 GN=NME4 PE=1 SV=1	O00746	NME4	21 kDa	5	0
20	40S ribosomal protein S2 OS=Homo sapiens OX=9606 GN=RPS2 PE=1 SV=2	P15880	RPS2	31 kDa	4	0
21	Endoplasmic reticulum chaperone BiP OS=Homo sapiens OX=9606 GN=HSPA5 PE=1 SV=2	P11021	HSPA5	72 kDa	4	0
22	Actin, cytoplasmic 1 OS=Homo sapiens OX=9606 GN=ACTB PE=1 SV=1	P60709 (+1)	ACTB	42 kDa	0	8
23	Protein-L-isoaspartate(D-aspartate) O-methyltransferase OS=Homo sapiens OX=9606 GN=PCMT1 PE=1 SV=4	P22061	PCMT1	25 kDa	0	3
24	Dermcidin OS=Homo sapiens OX=9606 GN=DCD PE=1 SV=2	P81605	DCD	11 kDa	0	3

cell line:					**unique peptide count**	
**HCT116**	**Identified Proteins**	Accession Number	Alternate ID	MW	Anti-Tks4-IP sample	control sample
1	SH3 and PX domain-containing protein 2B OS=Homo sapiens OX=9606 GN=SH3PXD2B PE=1 SV=3	SPD2B_HUMAN	SH3PXD2B	102 kDa	16	0
2	Plectin OS=Homo sapiens OX=9606 GN=PLEC PE=1 SV=3	PLEC_HUMAN	PLEC	532 kDa	116	1
3	CD2-associated protein OS=Homo sapiens OX=9606 GN=CD2AP PE=1 SV=1	CD2AP_HUMAN	CD2AP	71 kDa	23	0
4	Cell division cycle and apoptosis regulator protein 1 OS=Homo sapiens OX=9606 GN=CCAR1 PE=1 SV=2	CCAR1_HUMAN	CCAR1	133 kDa	32	0
5	F-actin-capping protein subunit alpha-1 OS=Homo sapiens OX=9606 GN=CAPZA1 PE=1 SV=3	CAZA1_HUMAN	CAPZA1	33 kDa	9	1
6	Tryptophan--tRNA ligase, cytoplasmic OS=Homo sapiens OX=9606 GN=WARS1 PE=1 SV=2	SYWC_HUMAN	WARS1	53 kDa	10	0
7	Kinesin-1 heavy chain OS=Homo sapiens OX=9606 GN=KIF5B PE=1 SV=1	KINH_HUMAN	KIF5B	110 kDa	15	0
8	F-actin-capping protein subunit beta OS=Homo sapiens OX=9606 GN=CAPZB PE=1 SV=4	CAPZB_HUMAN	CAPZB	31 kDa	8	0
9	SH3 domain-binding protein 1 OS=Homo sapiens OX=9606 GN=SH3BP1 PE=1 SV=3	3BP1_HUMAN	SH3BP1	76 kDa	4	0
10	SWISS-PROT:Q3Y5Z3 (Bos taurus) Adiponectin precursor	Q3Y5Z3 (+1)		26 kDa	5	0
11	SH3 domain-containing kinase-binding protein 1 OS=Homo sapiens OX=9606 GN=SH3KBP1 PE=1 SV=2	SH3K1_HUMAN	SH3KBP1	73 kDa	9	0
12	Cytoplasmic dynein 1 heavy chain 1 OS=Homo sapiens OX=9606 GN=DYNC1H1 PE=1 SV=5	DYHC1_HUMAN	DYNC1H1	532 kDa	3	0
13	F-actin-capping protein subunit alpha-2 OS=Homo sapiens OX=9606 GN=CAPZA2 PE=1 SV=3	CAZA2_HUMAN	CAPZA2	33 kDa	3	0
14	Probable ATP-dependent RNA helicase DDX17 OS=Homo sapiens OX=9606 GN=DDX17 PE=1 SV=2	DDX17_HUMAN	DDX17	80 kDa	3	1
15	Polyadenylate-binding protein 4 OS=Homo sapiens OX=9606 GN=PABPC4 PE=1 SV=1	PABP4_HUMAN	PABPC4	71 kDa	4	1

16	Nuclear fragile X mental retardation-interacting protein 2 OS=Homo sapiens OX=9606 GN=NUFIP2 PE=1 SV=1	NUFP2_HUMAN	NUFIP2	76 kDa	3	1
17	ATPase family AAA domain-containing protein 3A OS=Homo sapiens OX=9606 GN=ATAD3A PE=1 SV=2	ATD3A_HUMAN	ATAD3A	71 kDa	4	0
18	60S ribosomal protein L13 OS=Homo sapiens OX=9606 GN=RPL13 PE=1 SV=4	RL13_HUMAN	RPL13	24 kDa	5	2
19	Spermatogenesis-associated serine-rich protein 2 OS=Homo sapiens OX=9606 GN=SPATS2 PE=1 SV=1	SPAS2_HUMAN	SPATS2	60 kDa	4	0
20	Microtubule-associated protein 4 OS=Homo sapiens OX=9606 GN=MAP4 PE=1 SV=3	MAP4_HUMAN	MAP4	121 kDa	6	0
21	60S ribosomal protein L6 OS=Homo sapiens OX=9606 GN=RPL6 PE=1 SV=3	RL6_HUMAN	RPL6	33 kDa	3	0
22	Fibronectin OS=Homo sapiens OX=9606 GN=FN1 PE=1 SV=5	FINC_HUMAN	FN1	272 kDa	6	0

cell line:					**unique peptide count**	
**MCF7**	**Identified Proteins**	Accession Number	Alternate ID	MW	Anti-Tks4-IP sample	control sample
1	SH3 and PX domain-containing protein 2B OS=Homo sapiens OX=9606 GN=SH3PXD2B PE=1 SV=3	SPD2B_HUMAN	SH3PXD2B	102 kDa	23	1
2	Heterogeneous nuclear ribonucleoproteins A2/B1 OS=Homo sapiens OX=9606 GN=HNRNPA2B1 PE=1 SV=2	ROA2_HUMAN	HNRNPA2B1	37 kDa	3	0
3	Heterogeneous nuclear ribonucleoprotein A1 OS=Homo sapiens OX=9606 GN=HNRNPA1 PE=1 SV=5	ROA1_HUMAN	HNRNPA1	39 kDa	3	0
4	60 kDa heat shock protein, mitochondrial OS=Homo sapiens OX=9606 GN=HSPD1 PE=1 SV=2	CH60_HUMAN	HSPD1	61 kDa	3	0
5	Pyruvate kinase PKM OS=Homo sapiens OX=9606 GN=PKM PE=1 SV=4	KPYM_HUMAN	PKM	58 kDa	4	0
6	Tubulin alpha-1A chain OS=Homo sapiens OX=9606 GN=TUBA1A PE=1 SV=1	TBA1A_HUMAN	TUBA1A	50 kDa	3	0
7	Nucleophosmin OS=Homo sapiens OX=9606 GN=NPM1 PE=1 SV=2	NPM_HUMAN	NPM1	33 kDa	4	0
8	Calmodulin-like protein 5 OS=Homo sapiens OX=9606 GN=CALML5 PE=1 SV=2	CALL5_HUMAN	CALML5	16 kDa	5	2
9	Plectin OS=Homo sapiens OX=9606 GN=PLEC PE=1 SV=3	PLEC_HUMAN	PLEC	532 kDa	119	0
10	Cell division cycle and apoptosis regulator protein 1 OS=Homo sapiens OX=9606 GN=CCAR1 PE=1 SV=2	CCAR1_HUMAN	CCAR1	133 kDa	27	0
11	CD2-associated protein OS=Homo sapiens OX=9606 GN=CD2AP PE=1 SV=1	CD2AP_HUMAN	CD2AP	71 kDa	23	0
12	Heat shock cognate 71 kDa protein OS=Homo sapiens OX=9606 GN=HSPA8 PE=1 SV=1	HSP7C_HUMAN	HSPA8	71 kDa	11	3
13	Tryptophan--tRNA ligase, cytoplasmic OS=Homo sapiens OX=9606 GN=WARS1 PE=1 SV=2	SYWC_HUMAN	WARS1	53 kDa	6	0
14	F-actin-capping protein subunit alpha-1 OS=Homo sapiens OX=9606 GN=CAPZA1 PE=1 SV=3	CAZA1_HUMAN	CAPZA1	33 kDa	4	0
15	Kinesin-1 heavy chain OS=Homo sapiens OX=9606 GN=KIF5B PE=1 SV=1	KINH_HUMAN	KIF5B	110 kDa	18	0
16	F-actin-capping protein subunit beta OS=Homo sapiens OX=9606 GN=CAPZB PE=1 SV=4	CAPZB_HUMAN	CAPZB	31 kDa	7	0
17	SWISS-PROT:Q2UVX4 (Bos taurus) Complement C3 precursor	Q2UVX4 (+1)		187 kDa	5	0
18	Heat shock 70 kDa protein 1A OS=Homo sapiens OX=9606 GN=HSPA1A PE=1 SV=1	HS71A_HUMAN (+1)	HSPA1A	70 kDa	4	1
19	Microtubule-associated protein 4 OS=Homo sapiens OX=9606 GN=MAP4 PE=1 SV=3	MAP4_HUMAN	MAP4	121 kDa	14	0
20	Filamin-A OS=Homo sapiens OX=9606 GN=FLNA PE=1 SV=4	FLNA_HUMAN	FLNA	281 kDa	7	0
21	Cytoplasmic dynein 1 heavy chain 1 OS=Homo sapiens OX=9606 GN=DYNC1H1 PE=1 SV=5	DYHC1_HUMAN	DYNC1H1	532 kDa	4	1
22	Calmodulin-like protein 5 OS=Homo sapiens OX=9606 GN=CALML5 PE=1 SV=2	CALL5_HUMAN	CALML5	16 kDa	5	2
23	SH3 domain-containing kinase-binding protein 1 OS=Homo sapiens OX=9606 GN=SH3KBP1 PE=1 SV=2	SH3K1_HUMAN	SH3KBP1	73 kDa	3	0

24	Galectin-3 OS=Homo sapiens OX=9606 GN=LGALS3 PE=1 SV=5	LEG3_HUMAN	LGALS3	26 kDa	5	0
25	Genetic suppressor element 1 OS=Homo sapiens OX=9606 GN=GSE1 PE=1 SV=3	GSE1_HUMAN	GSE1	136 kDa	8	0
26	Glyceraldehyde-3-phosphate dehydrogenase OS=Homo sapiens OX=9606 GN=GAPDH PE=1 SV=3	G3P_HUMAN	GAPDH	36 kDa	3	0
27	Desmoglein-1 OS=Homo sapiens OX=9606 GN=DSG1 PE=1 SV=2	DSG1_HUMAN	DSG1	114 kDa	4	2
28	ATPase family AAA domain-containing protein 3A OS=Homo sapiens OX=9606 GN=ATAD3A PE=1 SV=2	ATD3A_HUMAN	ATAD3A	71 kDa	1	0
29	Kinesin light chain 1 OS=Homo sapiens OX=9606 GN=KLC1 PE=1 SV=2	KLC1_HUMAN	KLC1	65 kDa	3	0
30	Lysine-specific histone demethylase 1A OS=Homo sapiens OX=9606 GN=KDM1A PE=1 SV=2	KDM1A_HUMAN	KDM1A	93 kDa	4	0
31	Desmoplakin OS=Homo sapiens OX=9606 GN=DSP PE=1 SV=3	DESP_HUMAN	DSP	332 kDa	5	0
32	sp|Q9Y5Y9|SCNAA_HUMAN	SCNAA_HUMAN-DECOY		?	3	0

cell line:					**unique peptide count**	
**HPAC**	**Identified Proteins**	Accession Number	Alternate ID	MW	Anti-Tks4-IP sample	control sample
1	SH3 and PX domain-containing protein 2B OS=Homo sapiens OX=9606 GN=SH3PXD2B PE=1 SV=3	SPD2B_HUMAN	SH3PXD2B	102 kDa	4	0
2	Heat shock cognate 71 kDa protein OS=Homo sapiens OX=9606 GN=HSPA8 PE=1 SV=1	HSP7C_HUMAN	HSPA8	71 kDa	20	2
3	Nucleolin OS=Homo sapiens OX=9606 GN=NCL PE=1 SV=3	NUCL_HUMAN	NCL	77 kDa	11	1
4	Probable ATP-dependent RNA helicase DDX5 OS=Homo sapiens OX=9606 GN=DDX5 PE=1 SV=1	DDX5_HUMAN	DDX5	69 kDa	8	1
5	Endoplasmic reticulum chaperone BiP OS=Homo sapiens OX=9606 GN=HSPA5 PE=1 SV=2	BIP_HUMAN	HSPA5	72 kDa	12	1
6	Heat shock 70 kDa protein 1A OS=Homo sapiens OX=9606 GN=HSPA1A PE=1 SV=1	HS71A_HUMAN (+1)	HSPA1A	70 kDa	6	1
7	Splicing factor, proline- and glutamine-rich OS=Homo sapiens OX=9606 GN=SFPQ PE=1 SV=2	SFPQ_HUMAN	SFPQ	76 kDa	7	2
8	Lamina-associated polypeptide 2, isoform alpha OS=Homo sapiens OX=9606 GN=TMPO PE=1 SV=2	LAP2A_HUMAN	TMPO	75 kDa	17	2
9	Heterogeneous nuclear ribonucleoprotein U OS=Homo sapiens OX=9606 GN=HNRNPU PE=1 SV=6	HNRPU_HUMAN	HNRNPU	91 kDa	6	2
10	Polyadenylate-binding protein 1 OS=Homo sapiens OX=9606 GN=PABPC1 PE=1 SV=2	PABP1_HUMAN	PABPC1	71 kDa	7	0
11	Heterogeneous nuclear ribonucleoprotein K OS=Homo sapiens OX=9606 GN=HNRNPK PE=1 SV=1	HNRPK_HUMAN	HNRNPK	51 kDa	7	1
12	Heterogeneous nuclear ribonucleoprotein Q OS=Homo sapiens OX=9606 GN=SYNCRIP PE=1 SV=2	HNRPQ_HUMAN	SYNCRIP	70 kDa	4	1
13	40S ribosomal protein S4, X isoform OS=Homo sapiens OX=9606 GN=RPS4X PE=1 SV=2	RS4X_HUMAN	RPS4X	30 kDa	6	1
14	X-ray repair cross-complementing protein 6 OS=Homo sapiens OX=9606 GN=XRCC6 PE=1 SV=2	XRCC6_HUMAN	XRCC6	70 kDa	3	0
15	Heat shock protein HSP 90-alpha OS=Homo sapiens OX=9606 GN=HSP90AA1 PE=1 SV=5	HS90A_HUMAN	HSP90AA1	85 kDa	4	1
16	FACT complex subunit SSRP1 OS=Homo sapiens OX=9606 GN=SSRP1 PE=1 SV=1	SSRP1_HUMAN	SSRP1	81 kDa	3	0
17	Cell division cycle and apoptosis regulator protein 1 OS=Homo sapiens OX=9606 GN=CCAR1 PE=1 SV=2	CCAR1_HUMAN	CCAR1	133 kDa	33	0
18	CD2-associated protein OS=Homo sapiens OX=9606 GN=CD2AP PE=1 SV=1	CD2AP_HUMAN	CD2AP	71 kDa	31	0
19	Tryptophan--tRNA ligase, cytoplasmic OS=Homo sapiens OX=9606 GN=WARS1 PE=1 SV=2	SYWC_HUMAN	WARS1	53 kDa	17	0
20	F-actin-capping protein subunit alpha-1 OS=Homo sapiens OX=9606 GN=CAPZA1 PE=1 SV=3	CAZA1_HUMAN	CAPZA1	33 kDa	12	1

21	Kinesin-1 heavy chain OS=Homo sapiens OX=9606 GN=KIF5B PE=1 SV=1	KINH_HUMAN	KIF5B	110 kDa	12	0
22	F-actin-capping protein subunit beta OS=Homo sapiens OX=9606 GN=CAPZB PE=1 SV=4	CAPZB_HUMAN	CAPZB	31 kDa	12	0
23	Hemoglobin subunit beta OS=Homo sapiens OX=9606 GN=HBB PE=1 SV=2	HBB_HUMAN	HBB	16 kDa	10	0
24	Hemoglobin subunit alpha OS=Homo sapiens OX=9606 GN=HBA1 PE=1 SV=2	HBA_HUMAN	HBA1	15 kDa	6	0
25	Histone H3.2 OS=Homo sapiens OX=9606 GN=H3C15 PE=1 SV=3	H32_HUMAN	H3C15	15 kDa	10	2
26	60S ribosomal protein L23a OS=Homo sapiens OX=9606 GN=RPL23A PE=1 SV=1	RL23A_HUMAN	RPL23A	18 kDa	4	1
27	Heterogeneous nuclear ribonucleoproteins C1/C2 OS=Homo sapiens OX=9606 GN=HNRNPC PE=1 SV=4	HNRPC_HUMAN	HNRNPC	34 kDa	6	2
28	Microtubule-associated protein 4 OS=Homo sapiens OX=9606 GN=MAP4 PE=1 SV=3	MAP4_HUMAN	MAP4	121 kDa	6	0
29	Nucleophosmin OS=Homo sapiens OX=9606 GN=NPM1 PE=1 SV=2	NPM_HUMAN	NPM1	33 kDa	3	2
30	Matrin-3 OS=Homo sapiens OX=9606 GN=MATR3 PE=1 SV=2	MATR3_HUMAN	MATR3	95 kDa	8	2
31	40S ribosomal protein S7 OS=Homo sapiens OX=9606 GN=RPS7 PE=1 SV=1	RS7_HUMAN	RPS7	22 kDa	7	0
32	60S ribosomal protein L9 OS=Homo sapiens OX=9606 GN=RPL9 PE=1 SV=1	RL9_HUMAN	RPL9	22 kDa	3	1
33	F-actin-capping protein subunit alpha-2 OS=Homo sapiens OX=9606 GN=CAPZA2 PE=1 SV=3	CAZA2_HUMAN	CAPZA2	33 kDa	6	0
34	Core histone macro-H2A.1 OS=Homo sapiens OX=9606 GN=MACROH2A1 PE=1 SV=4	H2AY_HUMAN	MACROH2A1	40 kDa	4	1
35	Cytoplasmic dynein 1 heavy chain 1 OS=Homo sapiens OX=9606 GN=DYNC1H1 PE=1 SV=5	DYHC1_HUMAN	DYNC1H1	532 kDa	8	0
36	Annexin A2 OS=Homo sapiens OX=9606 GN=ANXA2 PE=1 SV=2	ANXA2_HUMAN	ANXA2	39 kDa	12	2
37	SH3 domain-containing kinase-binding protein 1 OS=Homo sapiens OX=9606 GN=SH3KBP1 PE=1 SV=2	SH3K1_HUMAN	SH3KBP1	73 kDa	9	0
38	Brain-specific angiogenesis inhibitor 1-associated protein 2-like protein 1 OS=Homo sapiens OX=9606 GN=BAIAP2L1 PE=1 SV=2	BI2L1_HUMAN	BAIAP2L1	57 kDa	11	0
39	Poly(U)-binding-splicing factor PUF60 OS=Homo sapiens OX=9606 GN=PUF60 PE=1 SV=1	PUF60_HUMAN	PUF60	60 kDa	4	0
40	40S ribosomal protein S11 OS=Homo sapiens OX=9606 GN=RPS11 PE=1 SV=3	RS11_HUMAN	RPS11	18 kDa	7	0
41	Complement C4-A OS=Homo sapiens OX=9606 GN=C4A PE=1 SV=2	CO4A_HUMAN	C4A	193 kDa	8	0
42	RNA-binding motif protein, X chromosome OS=Homo sapiens OX=9606 GN=RBMX PE=1 SV=3	RBMX_HUMAN	RBMX	42 kDa	4	1
43	60S acidic ribosomal protein P0 OS=Homo sapiens OX=9606 GN=RPLP0 PE=1 SV=1	RLA0_HUMAN	RPLP0	34 kDa	4	1
44	Sorcin OS=Homo sapiens OX=9606 GN=SRI PE=1 SV=1	SORCN_HUMAN	SRI	22 kDa	5	0
45	Regulation of nuclear pre-mRNA domain-containing protein 1B OS=Homo sapiens OX=9606 GN=RPRD1B PE=1 SV=1	RPR1B_HUMAN	RPRD1B	37 kDa	7	0
46	SH3 domain-binding protein 1 OS=Homo sapiens OX=9606 GN=SH3BP1 PE=1 SV=3	3BP1_HUMAN	SH3BP1	76 kDa	8	0
47	FACT complex subunit SPT16 OS=Homo sapiens OX=9606 GN=SUPT16H PE=1 SV=1	SP16H_HUMAN	SUPT16H	120 kDa	3	2
48	60S ribosomal protein L11 OS=Homo sapiens OX=9606 GN=RPL11 PE=1 SV=2	RL11_HUMAN	RPL11	20 kDa	5	0
49	60S ribosomal protein L12 OS=Homo sapiens OX=9606 GN=RPL12 PE=1 SV=1	RL12_HUMAN	RPL12	18 kDa	4	0
50	Poly [ADP-ribose] polymerase 1 OS=Homo sapiens OX=9606 GN=PARP1 PE=1 SV=4	PARP1_HUMAN	PARP1	113 kDa	6	1
51	60S ribosomal protein L10 OS=Homo sapiens OX=9606 GN=RPL10 PE=1 SV=4	RL10_HUMAN	RPL10	25 kDa	5	0
52	Histone H2B type 1-O OS=Homo sapiens OX=9606 GN=H2BC17 PE=1 SV=3	H2B1O_HUMAN	H2BC17	14 kDa	4	0
53	40S ribosomal protein S18 OS=Homo sapiens OX=9606 GN=RPS18 PE=1 SV=3	RS18_HUMAN	RPS18	18 kDa	4	0
54	Heterogeneous nuclear ribonucleoprotein H3 OS=Homo sapiens OX=9606 GN=HNRNPH3 PE=1 SV=2	HNRH3_HUMAN	HNRNPH3	37 kDa	3	1
55	RNA-binding protein 25 OS=Homo sapiens OX=9606 GN=RBM25 PE=1 SV=3	RBM25_HUMAN	RBM25	100 kDa	4	0
56	RNA-binding protein 39 OS=Homo sapiens OX=9606 GN=RBM39 PE=1 SV=2	RBM39_HUMAN	RBM39	59 kDa	6	0
57	Peroxiredoxin-1 OS=Homo sapiens OX=9606 GN=PRDX1 PE=1 SV=1	PRDX1_HUMAN	PRDX1	22 kDa	4	0
58	40S ribosomal protein S10 OS=Homo sapiens OX=9606 GN=RPS10 PE=1 SV=1	RS10_HUMAN	RPS10	19 kDa	3	0

59	ATPase family AAA domain-containing protein 3A OS=Homo sapiens OX=9606 GN=ATAD3A PE=1 SV=2	ATD3A_HUMAN	ATAD3A	71 kDa	4	0
60	Fibronectin OS=Homo sapiens OX=9606 GN=FN1 PE=1 SV=5	FINC_HUMAN	FN1	272 kDa	6	0
61	Alpha-2-macroglobulin OS=Homo sapiens OX=9606 GN=A2M PE=1 SV=3	A2MG_HUMAN	A2M	163 kDa	3	0
62	Heterogeneous nuclear ribonucleoprotein F OS=Homo sapiens OX=9606 GN=HNRNPF PE=1 SV=3	HNRPF_HUMAN	HNRNPF	46 kDa	3	0
63	60S ribosomal protein L18 OS=Homo sapiens OX=9606 GN=RPL18 PE=1 SV=2	RL18_HUMAN	RPL18	22 kDa	4	0
64	Kinesin light chain 1 OS=Homo sapiens OX=9606 GN=KLC1 PE=1 SV=2	KLC1_HUMAN	KLC1	65 kDa	4	0
65	Carcinoembryonic antigen-related cell adhesion molecule 5 OS=Homo sapiens OX=9606 GN=CEACAM5 PE=1 SV=3	CEAM5_HUMAN	CEACAM5	77 kDa	4	0
66	60S ribosomal protein L22 OS=Homo sapiens OX=9606 GN=RPL22 PE=1 SV=2	RL22_HUMAN	RPL22	15 kDa	4	0
67	60S ribosomal protein L23 OS=Homo sapiens OX=9606 GN=RPL23 PE=1 SV=1	RL23_HUMAN	RPL23	15 kDa	4	0
68	ELAV-like protein 1 OS=Homo sapiens OX=9606 GN=ELAVL1 PE=1 SV=2	ELAV1_HUMAN	ELAVL1	36 kDa	3	0
69	60S ribosomal protein L31 OS=Homo sapiens OX=9606 GN=RPL31 PE=1 SV=1	RL31_HUMAN	RPL31	14 kDa	5	0
70	Heterogeneous nuclear ribonucleoprotein R OS=Homo sapiens OX=9606 GN=HNRNPR PE=1 SV=1	HNRPR_HUMAN	HNRNPR	71 kDa	3	0
71	Single-stranded DNA-binding protein, mitochondrial OS=Homo sapiens OX=9606 GN=SSBP1 PE=1 SV=1	SSBP_HUMAN	SSBP1	17 kDa	5	0
72	Lamin-B1 OS=Homo sapiens OX=9606 GN=LMNB1 PE=1 SV=2	LMNB1_HUMAN	LMNB1	66 kDa	4	0
73	60S ribosomal protein L18a OS=Homo sapiens OX=9606 GN=RPL18A PE=1 SV=2	RL18A_HUMAN	RPL18A	21 kDa	4	0
74	REST corepressor 1 OS=Homo sapiens OX=9606 GN=RCOR1 PE=1 SV=2	RCOR1_HUMAN	RCOR1	53 kDa	3	0
75	Laminin subunit alpha-3 OS=Homo sapiens OX=9606 GN=LAMA3 PE=1 SV=2	LAMA3_HUMAN	LAMA3	367 kDa	4	0
76	40S ribosomal protein S14 OS=Homo sapiens OX=9606 GN=RPS14 PE=1 SV=3	RS14_HUMAN	RPS14	16 kDa	4	0
77	Optineurin OS=Homo sapiens OX=9606 GN=OPTN PE=1 SV=3	OPTN_HUMAN	OPTN	66 kDa	3	0
78	DNA topoisomerase 2-alpha OS=Homo sapiens OX=9606 GN=TOP2A PE=1 SV=3	TOP2A_HUMAN	TOP2A	174 kDa	3	0
79	Protein-glutamine gamma-glutamyltransferase 2 OS=Homo sapiens OX=9606 GN=TGM2 PE=1 SV=2	TGM2_HUMAN	TGM2	77 kDa	5	0
80	E3 ubiquitin-protein ligase TRIM21 OS=Homo sapiens OX=9606 GN=TRIM21 PE=1 SV=1	RO52_HUMAN	TRIM21	54 kDa	4	0
81	Coiled-coil domain-containing protein 7 OS=Homo sapiens OX=9606 GN=CCDC7 PE=1 SV=3	CCDC7_HUMAN	CCDC7	157 kDa	4	0
82	Epiplakin OS=Homo sapiens OX=9606 GN=EPPK1 PE=1 SV=3	EPIPL_HUMAN	EPPK1	556 kDa	3	0
83	Acylamino-acid-releasing enzyme OS=Homo sapiens OX=9606 GN=APEH PE=1 SV=4	ACPH_HUMAN	APEH	81 kDa	3	0
84	Sorbin and SH3 domain-containing protein 2 OS=Homo sapiens OX=9606 GN=SORBS2 PE=1 SV=3	SRBS2_HUMAN	SORBS2	124 kDa	3	0
85	Leucine-rich PPR motif-containing protein, mitochondrial OS=Homo sapiens OX=9606 GN=LRPPRC PE=1 SV=3	LPPRC_HUMAN	LRPPRC	158 kDa	4	0
86	60S ribosomal protein L35 OS=Homo sapiens OX=9606 GN=RPL35 PE=1 SV=2	RL35_HUMAN	RPL35	15 kDa	3	0
87	40S ribosomal protein S13 OS=Homo sapiens OX=9606 GN=RPS13 PE=1 SV=2	RS13_HUMAN	RPS13	17 kDa	3	0
88	Golgin subfamily A member 4 OS=Homo sapiens OX=9606 GN=GOLGA4 PE=1 SV=1	GOGA4_HUMAN	GOLGA4	261 kDa	3	0
89	60S ribosomal protein L24 OS=Homo sapiens OX=9606 GN=RPL24 PE=1 SV=1	RL24_HUMAN	RPL24	18 kDa	3	0

cell line:					**unique peptide count**	
**N87**	**Identified Proteins**	Accession Number	Alternate ID	MW	Anti-Tks4-IP sample	control sample

1	Actin, cytoplasmic 2 OS=Homo sapiens OX=9606 GN=ACTG1 PE=1 SV=1	P63261	ACTG1	42 kDa	14	0
2	Albumin OS=Homo sapiens OX=9606 GN=ALB PE=1 SV=2	P02768 (+2)	ALB	69 kDa	32	0
3	Alpha-actinin-4 OS=Homo sapiens OX=9606 GN=ACTN4 PE=1 SV=2	O43707	ACTN4	105 kDa	4	0
4	Alpha-amylase 1B OS=Homo sapiens OX=9606 GN=AMY1B PE=1 SV=1	P0DTE7 (+2)	AMY1B	58 kDa	6	4
5	CD2-associated protein OS=Homo sapiens OX=9606 GN=CD2AP PE=1 SV=1	Q9Y5K6	CD2AP	71 kDa	9	0
6	Cell division cycle and apoptosis regulator protein 1 OS=Homo sapiens OX=9606 GN=CCAR1 PE=1 SV=2	Q8IX12	CCAR1	133 kDa	8	0
7	Core histone macro-H2A.1 OS=Homo sapiens OX=9606 GN=MACROH2A1 PE=1 SV=4	O75367	MACROH2A1	40 kDa	4	0
8	Desmoplakin OS=Homo sapiens OX=9606 GN=DSP PE=1 SV=3	P15924	DSP	332 kDa	3	0
9	Endoplasmic reticulum chaperone BiP OS=Homo sapiens OX=9606 GN=HSPA5 PE=1 SV=2	P11021	HSPA5	72 kDa	3	0
10	F-actin-capping protein subunit alpha-1 OS=Homo sapiens OX=9606 GN=CAPZA1 PE=1 SV=3	P52907	CAPZA1	33 kDa	4	0
11	Fibronectin OS=Homo sapiens OX=9606 GN=FN1 PE=1 SV=5	P02751	FN1	272 kDa	5	0
12	Heat shock cognate 71 kDa protein OS=Homo sapiens OX=9606 GN=HSPA8 PE=1 SV=1	P11142	HSPA8	71 kDa	12	5
13	Heterogeneous nuclear ribonucleoprotein K OS=Homo sapiens OX=9606 GN=HNRNPK PE=1 SV=1	P61978	HNRNPK	51 kDa	3	0
14	Heterogeneous nuclear ribonucleoproteins A2/B1 OS=Homo sapiens OX=9606 GN=HNRNPA2B1 PE=1 SV=2	P22626	HNRNPA2B1	37 kDa	5	0
15	Histone H2A type 1-B/E OS=Homo sapiens OX=9606 GN=H2AC4 PE=1 SV=2	P04908 (+2)	H2AC4	14 kDa	4	0
16	Histone H2B type 1-D OS=Homo sapiens OX=9606 GN=H2BC5 PE=1 SV=2	P58876 (+6)	H2BC5	14 kDa	13	0
17	Histone H3.1 OS=Homo sapiens OX=9606 GN=H3C1 PE=1 SV=2	P68431	H3C1	15 kDa	5	0
18	Histone H4 OS=Homo sapiens OX=9606 GN=H4C1 PE=1 SV=2	P62805	H4C1	11 kDa	6	0
19	Hornerin OS=Homo sapiens OX=9606 GN=HRNR PE=1 SV=2	Q86YZ3 (+1)	HRNR	282 kDa	5	0
20	Immunoglobulin heavy constant gamma 1 OS=Homo sapiens OX=9606 GN=IGHG1 PE=1 SV=1	P01857 (+1)	IGHG1	36 kDa	6	0
21	Junction plakoglobin OS=Homo sapiens OX=9606 GN=JUP PE=1 SV=3	P14923	JUP	82 kDa	5	0
22	Keratin, type I cytoskeletal 14 OS=Homo sapiens OX=9606 GN=KRT14 PE=1 SV=4	P02533 (+1)	KRT14	52 kDa	3	0
23	Keratin, type I cytoskeletal 16 OS=Homo sapiens OX=9606 GN=KRT16 PE=1 SV=4	P08779 (+1)	KRT16	51 kDa	3	0
24	Keratin, type I cytoskeletal 18 OS=Homo sapiens OX=9606 GN=KRT18 PE=1 SV=2	P05783	KRT18	48 kDa	15	0
25	Nebulin OS=Homo sapiens OX=9606 GN=NEB PE=1 SV=5	P20929	NEB	773 kDa	3	0
26	Plectin OS=Homo sapiens OX=9606 GN=PLEC PE=1 SV=3	Q15149	PLEC	532 kDa	102	0
27	Polypyrimidine tract-binding protein 1 OS=Homo sapiens OX=9606 GN=PTBP1 PE=1 SV=1	P26599	PTBP1	57 kDa	3	0
28	Prelamin-A/C OS=Homo sapiens OX=9606 GN=LMNA PE=1 SV=1	P02545	LMNA	74 kDa	9	0
29	Probable ATP-dependent RNA helicase DDX5 OS=Homo sapiens OX=9606 GN=DDX5 PE=1 SV=1	P17844	DDX5	69 kDa	4	0
30	Serotransferrin OS=Homo sapiens OX=9606 GN=TF PE=1 SV=3	P02787	TF	77 kDa	3	0
31	SH3 and PX domain-containing protein 2B OS=Homo sapiens OX=9606 GN=SH3PXD2B PE=1 SV=3	A1X283	SH3PXD2B	102 kDa	5	0
32	Sorcin OS=Homo sapiens OX=9606 GN=SRI PE=1 SV=1	P30626	SRI	22 kDa	5	0
33	Stress-70 protein, mitochondrial OS=Homo sapiens OX=9606 GN=HSPA9 PE=1 SV=2	P38646	HSPA9	74 kDa	3	0
34	Titin OS=Homo sapiens OX=9606 GN=TTN PE=1 SV=4	Q8WZ42	TTN	3816 kDa	11	0
35	Transketolase OS=Homo sapiens OX=9606 GN=TKT PE=1 SV=3	P29401	TKT	68 kDa	3	0
36	Tryptophan--tRNA ligase, cytoplasmic OS=Homo sapiens OX=9606 GN=WARS1 PE=1 SV=2	P23381	WARS1	53 kDa	7	0
37	Actin, cytoplasmic 1 OS=Homo sapiens OX=9606 GN=ACTB PE=1 SV=1	P60709 (+1)	ACTB	42 kDa	0	4
38	Transformation/transcription domain-associated protein OS=Homo sapiens OX=9606 GN=TRRAP PE=1 SV=3	Q9Y4A5	TRRAP	438 kDa	0	3

Supplementary table 5: List of all validated protein-protein interactions within Tks4 signallome based on literature search used for the generation of Figure 5.

Tks4 partner molecules:	Partner molecules interacting within the network	Method used for the validation of the protein-protein interaction
**SRC (Proto-oncogene tyrosine-protein kinase Src)**	WASL/N-WASP	enzymatic study assay(1)
	Cortactin	in vitro and in vivo assays(2)<br>anti bait coimmunoprecipitation assay(3)<br>pull down assay(4)
	GRB2	affinity chromatography technology assay(5,6)<br>in vitro and in vivo assays(7)
	DNM2	affinity chromatography technology assay(8)<br>surface plasmon resonance assay(9)
	ADAM15	phage display assay(10)<br>pull down assay(11)<br>affinity chromatography technology assay(12)
	SH3KBP1/CIN85	affinity chromatography technology assay(13,14)<br>anti bait coimmunoprecipitation assay(15)
	NOXA1	in vitro assay(16)
	Tks4	Co-immunoprecipitation(17), Co-expression(18)<br>GST pull-down (17)<br>Fluorescence-polarization assay(3)<br>Duolink proximity ligation assay(3)
**Cortactin (Src substrate cortactin)**	WASL	far western blotting assay(19)<br>affinity chromatography technology assay(20)<br>anti tag coimmunoprecipitation assay(21)<br>fluorescence microscopy assay(22)

	DNM2	molecular sieving assay(23)<br>anti tag coimmunoprecipitation assay(21,24)<br>in vitro and in vivo assays(25)
	GRB2	anti tag coimmunoprecipitation assay(21)<br>in vitro assay(26)
	Tks4	Colocalization and co-immunoprecipitation(27,28)
**CAPZA1 (F-actin-capping protein subunit alpha-1)**	SH3KBP1/CIN85	anti bait/tag coimmunoprecipitation assay(15,29,30)<br>in vitro assay(31)
	CD2AP	pull down assay(31)<br>affinity chromatography technology assay(32)<br>anti bait/tag coimmunoprecipitation assay(15,30)
	CAPZB	crystal structure: (PDB entry 6f1u, 6f3a, 6f38, 6f1t)(33)<br>biochemical assay(34)
	Tks4	prediction analysis, mass spectrometry, immunoprecipitation, Duolink proximity ligation assay (this study)
**CAPZB (F-actin-capping subunit beta)**	Tks4	prediction analysis, mass spectrometry (this study)
**ADAM15 (Disintegrin and metalloproteinase domain-containing protein 15)**	GRB2	filter binding assay(35)<br>affinity chromatography technology assay(12)<br>pull down assay(11)

	Tks4	GST-pull down assay(36)
**DNM2 (Dynamin-2)**	SH3KBP1/CIN85	pull down assay(37)<br>affinity chromatography technology assay(38)<br>biochemical assay(34)
	GRB2	surface plasmon resonance assay(9,39)<br>anti tag coimmunoprecipitation assay(21)<br>barcode fusion genetics two hybrid assay(40)
	Tks4	GST-pull down assay(41)
**GRB2 (Growth factor receptor-bound protein 2)**	SH3KBP1	pull down assay(42,43)
	WASL	pull down assay(44)<br>anti tag coimmunoprecipitation assay(21)<br>barcode fusion genetics two hybrid assay(40)<br>in vitro and in vivo assays(45)
	WIPF3/CR16	anti tag coimmunoprecipitation assay(21)
	Tks4	Affinity purification–selected reaction monitoring mass spectrometry(21)
**WASL (Actin nucleation-promoting factor WASL)**	WIPF3/CR16	anti tag coimmunoprecipitation assay(21,30)
	Tks4	GST-pull down assay(41)

**SH3KBP1 (SH3 domain-containing kinase-binding protein 1)**	CD2AP	affinity chromatography technology assay(46,47)<br>anti bait/tag coimmunoprecipitation assay(15,30)
	CAPZB	affinity chromatography technology assay(29)<br>anti tag coimmunoprecipitation assay(30)
	Tks4	GST-pull down assay(48)
**CD2AP (CD2-associated protein)**	CAPZB	affinity chromatography technology assay(32)<br>anti tag coimmunoprecipitation assay(30)
	Tks4	mass spectrometry, Co-immunoprecipitation, Duolink proximity ligation assay, peptide array(49)
**WIPF3/CR16 (WAS/WASL-interacting protein family member 3)**	Tks4	GST-pull down assay(41)
**NOXA1 (NADPH oxidase activator 1)**	Tks4	Co-immunoprecipitation, GST pull-down assay(16)

1. Park SJ, Suetsugu S, Takenawa T. Interaction of HSP90 to N-WASP leads to activation and protection from proteasome-dependent degradation. EMBO J [Internet]. 2005 [cited 2023 Jun 1];24:1557–70. Available from: https://www.embopress.org/doi/10.1038/sj.emboj.7600586
2. Huang C, Liu J, Haudenschild CC, Zhan X. The Role of Tyrosine Phosphorylation of Cortactin in the Locomotion of Endothelial Cells*. 1998 [cited 2023 Jun 1]; Available from: http://www.jbc.org
3. Dülk M, Szeder B, Glatz G, Merö BL, Koprivanacz K, Kudlik G, et al. EGF Regulates the Interaction of Tks4 with Src through Its SH2 and SH3 Domains. Biochemistry [Internet]. 2018 Jul 17 [cited 2021 May 12];57(28):4186–96. Available from: https://pubmed.ncbi.nlm.nih.gov/29928795/
4. Han T, Jiang S, Zheng H, Yin Q, Xie M, Little MR, et al. Interplay between c-Src and the APC/C co-activator Cdh1 regulates mammary tumorigenesis. Nat Commun [Internet]. 2019 Dec 1 [cited 2023 Jun 1];10(1). Available from: /pmc/articles/PMC6697746/
5. Wan KF, Sambi BS, Tate R, Waters C, Pyne NJ. The inhibitory γ subunit of the type 6 retinal cGMP phosphodiesterase functions to link c-Src and G-protein-coupled receptor kinase 2 in a signaling unit that regulates p42/p44 mitogen-activated protein kinase by epidermal growth factor. J Biol Chem [Internet]. 2003;278(20):18658–63. Available from: http://dx.doi.org/10.1074/jbc.M212103200
6. Saci A, Liu WQ, Vidal M, Garbay C, Rendu F, Bachelot-Loza C. Differential effect of the inhibition of Grb2-SH3 interactions in platelet activation induced by thrombin and by Fc receptor engagement. Biochem J. 2002;363(3):717–25.
7. Jones DA, Benjamin CW. Phosphorylation of growth factor receptor binding protein-2 by pp60(c- src) tyrosine kinase. Arch Biochem Biophys. 1997;337(2):143–8.
8. Wang Y, Cao H, Chen J, McNiven MA. A direct interaction between the large GTPase dynamin-2 and FAK regulates focal adhesion dynamics in response to active Src. Mol Biol Cell. 2011;22(9):1529–38.
9. Solomaha E, Szeto FL, Yousef MA, Palfrey HC. Kinetics of Src homology 3 domain association with the proline-rich domain of dynamins: Specificity, occlusion, and the effects of phosphorylation. J Biol Chem [Internet]. 2005;280(24):23147–56. Available from: http://dx.doi.org/10.1074/jbc.M501745200
10. Kärkkäinen S, Hiipakka M, Wang J-H, Kleino I, Vähä-Jaakkola M, Renkema GH, et al. Identification of preferred protein

interactions by phage-display of the human Src homology-3 proteome. EMBO Rep. 2006;7(2):186–91.
11. Poghosyan Z, Robbins SM, Houslay MD, Webster A, Murphy G, Edwards DR. Phosphorylation-dependent interactions between ADAM15 cytoplasmic domain and Src family protein-tyrosine kinases. J Biol Chem [Internet]. 2002;277(7):4999–5007. Available from: http://dx.doi.org/10.1074/jbc.M107430200
12. Zhong JL, Poghosyan Z, Pennington CJ, Scott X, Handsley MM, Warn A, et al. Distinct functions of natural ADAM-15 cytoplasmic domain variants in human mammary carcinoma. Mol Cancer Res. 2008;6(3):383–94.
13. Narita T, Nishimura T, Yoshizaki K, Taniyama T. CIN85 associates with TNF receptor 1 via Src and modulates TNF-α-induced apoptosis. Exp Cell Res. 2005;304(1):256–64.
14. Schroeder B, Srivatsan S, Shaw A, Billadeau D, McNiven MA. CIN85 phosphorylation is essential for EGFR ubiquitination and sorting into multivesicular bodies. Mol Biol Cell. 2012;23(18):3602–11.
15. Kourtidis A, Necela B, Lin WH, Lu R, Feathers RW, Asmann YW, et al. Cadherin complexes recruit mRNAs and RISC to regulate epithelial cell signaling. J Cell Biol [Internet]. 2017 Oct 1 [cited 2023 Jun 1];216(10):3073–85. Available from: https://pubmed.ncbi.nlm.nih.gov/28877994/
16. Emerman AB, Zhang Z-R, Chakrabarti O, Hegde RS. c-Src-mediated phosphorylation of NOXA1 and Tks induces the reactive oxygen species (ROS)-dependent formation of functional invadopodia in human colon cancer cells. Mol Biol Cell. 2010;21(24):4325–37.
17. Bögel G, Gujdár A, Geiszt M, Lányi Á, Fekete A, Sipeki S, et al. Frank-ter Haar Syndrome Protein Tks4 Regulates Epidermal Growth Factor-dependent Cell Migration. J Biol Chem. 2012 Sep 7;287(37):31321–9.
18. Buschman MD, Bromann PA, Cejudo-Martin P, Wen F, Pass I, Courtneidge SA. The novel adaptor protein Tks4 (SH3PXD2B) is required for functional podosome formation. Mol Biol Cell. 2009 Mar;20(5):1302–11.
19. Mizutani K, Miki H, Takenawa T, Maruta H, Takenawa T, He H, et al. Essential role of neural Wiskott-Aldrich syndrome protein in podosome formation and degradation of extracellular matrix in src-transformed fibroblasts. Cancer Res. 2002;62(3):669–74.
20. Lynch DK, Winata SC, Lyons RJ, Hughes WE, Lehrbach GM, Wasinger V, et al. A cortactin-CD2-associated protein (CD2AP) complex provides a novel link between epidermal growth factor receptor endocytosis and the actin cytoskeleton. J Biol Chem [Internet]. 2003;278(24):21805–13. Available from: http://dx.doi.org/10.1074/jbc.M211407200
21. Bisson N, James DA, Ivosev G, Tate SA, Bonner R, Taylor L, et al. Selected reaction monitoring mass spectrometry reveals the dynamics of signaling through the GRB2 adaptor. Nat Biotechnol [Internet]. 2011;29(7):653–8. Available from: https://doi.org/10.1038/nbt.1905
22. Grassart A, Dujeancourt A, Lazarow PB, Dautry-Varsat A, Sauvonnet N. Clathrin-independent endocytosis used by the IL-2 receptor is regulated by Rac1, Pak1 and Pak2. EMBO Rep. 2008;9(4):356–62.
23. Hashimoto S, Hirose M, Hashimoto A, Morishige M, Yamada A, Hosaka H, et al. Targeting AMAP1 and cortactin binding bearing an atypical src homology 3/proline interface for prevention of breast cancer invasion and metastasis. Proc Natl Acad Sci U S A. 2006;103(18):7036–41.
24. Lee JH, Han JH, Kim H, Park SM, Joe EH, Jou I. Parkinson's disease-associated LRRK2-G2019S mutant acts through regulation of SERCA activity to control ER stress in astrocytes. Acta Neuropathol Commun. 2019;7(1):68.
25. Schafer DA, Weed SA, Binns D, Karginov A V., Parsons JT, Cooper JA. Dynamin2 and cortactin regulate actin assembly and filament organization. Curr Biol. 2002;12(21):1852–7.
26. Crostella L, Lidder S, Williams R, Skouteris GG. Hepatocyte Growth Factor/Scatter Factor-induces phosphorylation of cortactin in A431 cells in a Src kinase-independent manner. Oncogene [Internet]. 2001;20(28):3735–45. Available from: https://doi.org/10.1038/sj.onc.1204474
27. Lányi Á, Baráth M, Péterfi Z, Bőgel G, Orient A, Simon T, et al. The Homolog of the Five SH3-Domain Protein (HOFI/SH3PXD2B) Regulates Lamellipodia Formation and Cell Spreading. Csermely P, editor. PLoS One [Internet]. 2011 Aug 23;6(8):e23653. Available from: https://dx.plos.org/10.1371/journal.pone.0023653
28. Leong HS, Robertson AE, Stoletov K, Leith SJ, Chin CA, Chien AE, et al. Invadopodia Are Required for Cancer Cell Extravasation and Are a Therapeutic Target for Metastasis. Cell Rep [Internet]. 2014;8(5):1558–70. Available from: http://dx.doi.org/10.1016/j.celrep.2014.07.050
29. Bior BK, Ballif BA. Dab1 stabilizes its interaction with Cin85 by suppressing Cin85 phosphorylation at serine 587. FEBS Lett [Internet]. 2013;587(1):60–6. Available from: http://dx.doi.org/10.1016/j.febslet.2012.10.051
30. Huttlin EL, Bruckner RJ, Paulo JA, Cannon JR, Ting L, Baltier K, et al. Architecture of the human interactome defines protein communities and disease networks. Nature [Internet]. 2017;545(7655):505–9. Available from: http://dx.doi.org/10.1038/nature22366
31. Hutchingst NJ, Clarkson N, Chalkley R, Barclay AN, Brown MH. Linking the T cell surface protein CD2 to the actin-capping protein CAPZ via CMS and CIN85. J Biol Chem [Internet]. 2003;278(25):22396–403. Available from: http://dx.doi.org/10.1074/jbc.M302540200
32. Bao M, Hanabuchi S, Facchinetti V, Du Q, Bover L, Plumas J, et al. CD2AP/SHIP1 Complex Positively Regulates Plasmacytoid Dendritic Cell Receptor Signaling by Inhibiting the E3 Ubiquitin Ligase Cbl. J Immunol. 2012;189(2):786–92.
33. Urnavicius L, Lau CK, Elshenawy MM, Morales-Rios E, Motz C, Yildiz A, et al. Cryo-EM shows how dynactin recruits two dyneins for faster movement. Nature. 2018;554(7691):202–6.
34. Wan C, Borgeson B, Phanse S, Tu F, Drew K, Clark G, et al. Panorama of ancient metazoan macromolecular complexes. Nature. 2015;525(7569):339–44.
35. Kärkkäinen S, Hiipakka M, Wang JH, Kleino I, Vähä-Jaakkola M, Renkema GH, et al. Identification of preferred protein interactions by phage-display of the human Src homology-3 proteome. EMBO Rep. 2006;7(2):186–91.
36. Ae MM, Thedens DR, Bo AE, Ae C, Ae BSH, Yin Q, et al. The podosomal-adaptor protein SH3PXD2B is essential for normal postnatal development. Available from: http://mousemutant.jax.org/index.html
37. Schroeder B, Weller SG, Chen J, Billadeau D, McNiven MA. A Dyn2-CIN85 complex mediates degradative traffic of the EGFR by regulation of late endosomal budding. EMBO J [Internet]. 2010;29(18):3039–53. Available from: http://dx.doi.org/10.1038/emboj.2010.190
38. Havrylov S, Rzhepetskyy Y, Malinowska A, Drobot L, Redowicz MJ. Proteins recruited by SH3 domains of Ruk/CIN85 adaptor identified by LC-MS/MS. Proteome Sci. 2009;7.
39. Elhamdani A, Azizi F, Solomaha E, Palfrey HC, Artalejo CR. Two mechanistically distinct forms of endocytosis in adrenal chromaffin cells: Differential effects of SH3 domains and amphiphysin antagonism. FEBS Lett. 2006;580(13):3263–9.
40. Yachie N, Petsalaki E, Mellor JC, Weile J, Jacob Y, Verby M, et al. Pooled-matrix protein interaction screens using Barcode Fusion Genetics. Mol Syst Biol. 2016;12(4):863.
41. Kropyvko S V. New partners of TKS4 scaffold protein. Biopolym Cell. 2015;31(5):395–401.
42. Gout I, Middleton G, Adu J, Ninkina NN, Drobot LB, Filonenko V, et al. Negative regulation of PI 3-kinase by Ruk, a novel adaptor protein. EMBO J. 2000;19(15):4015–25.

43. Borinstein SC, Hyatt MA, Sykes VW, Straub RE, Lipkowitz S, Boulter J, et al. SETA is a multifunctional adapter protein with three SH3 domains that binds Grb2, Cbl, and the novel SB1 proteins. Cell Signal. 2000;12(11–12):769–79.
44. Carlier MF, Nioche P, Broutin-L'hermite I, Boujemaa R, Le Clainche C, Egile C, et al. GRB2 links signaling to actin assembly by enhancing interaction of neural wiskott-aldrich syndrome protein (N-WASp) with actin-related protein (ARP2/3) complex. J Biol Chem [Internet]. 2000;275(29):21946–52. Available from: http://dx.doi.org/10.1074/jbc.M000687200
45. Ramesh N, Antón IM, And NM-Q, Geha RS. Waltzing with WASP. Trends Cell Biol [Internet]. 9. Available from: https://search.proquest.com/docview/229488101?accountid=27308
46. Tossidou I, Teng B, Drobot L, Meyer-Schwesinger C, Worthmann K, Haller H, et al. CIN85/RukL is a novel binding partner of nephrin and podocin and mediates slit diaphragm turnover in podocytes. J Biol Chem. 2010;285(33):25285–95.
47. Gaidos G, Soni S, Oswald DJ, Toselli PA, Kirsch KH. Structure and function analysis of the CMS/CIN85 protein family identifies actin-bundling properties and heterotypic-complex formation. J Cell Sci. 2007;120(14):2366–77.
48. Bazalii A V., Samoylenko AA, Petukhov DM, Rynditch A V., Redowicz MJ, Drobot LB. Interaction between adaptor proteins Ruk/CIN85 and Tks4 in normal and tumor cells of different tissue origins. Biopolym Cell. 2015;30(1):37–41.
49. Kurilla A, László L, Takács T, Tilajka Á, Lukács L, Novák J, et al. Exploring the Interdependence of TKS4 and CD2AP: Implications for EMT Process and Early Detection in Colon Cancer. bioRxiv. 2023;
50. Lányi Á, Baráth M, Péterfi Z, Bögel G, Orient A, Simon T, et al. The Homolog of the Five SH3-Domain Protein (HOFI/SH3PXD2B) Regulates Lamellipodia Formation and Cell Spreading. PLoS One [Internet]. 2011 Aug 23 [cited 2022 Jun 3];6(8):e23653. Available from: https://journals.plos.org/plosone/article?id=10.1371/journal.pone.0023653
